# Concatenating Suzuki Arylation and Buchwald–Hartwig Amination by A Sequentially Pd‐Catalyzed One‐Pot Process—Consecutive Three‐Component Synthesis of *C*,*N*‐Diarylated Heterocycles

**DOI:** 10.1002/chem.202003837

**Published:** 2020-10-19

**Authors:** Laura Mayer, Regina Kohlbecher, Thomas J. J. Müller

**Affiliations:** ^1^ Institut für Organische Chemie und Makromolekulare Chemie Heinrich-Heine-Universität Düsseldorf Universitätsstraße 1 40225 Düsseldorf Germany

**Keywords:** cross-coupling, heterocycles, multicomponent reactions, one-pot reactions, sequential palladium catalysis

## Abstract

The concatenation of Suzuki coupling and Buchwald‐Hartwig amination in a consecutive multicomponent reaction opens a concise, modular and efficient one‐pot approach to diversely functionalized heterocycles, as exemplified for 3,10‐diaryl 10*H*‐phenothiazines, 3,9‐diaryl 9*H*‐carbazoles, and 1,5‐diaryl 1*H*‐indoles, in high yields starting from simple staring materials. Moreover, this one‐pot reaction is a sequentially palladium‐catalyzed process that does not require additional catalyst loading after the first coupling step.

## Graphical abstract

**Catch two birds with one pot**! A sequentially Pd‐catalyzed Suzuki Buchwald–Hartwig process allows the efficient consecutive three‐component synthesis of *C*,*N*‐diarylated heterocycles.

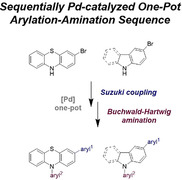

Pd‐catalyzed carbon–carbon and carbon–nitrogen bond formations by Suzuki arylation and Buchwald–Hartwig amination currently represent the most versatile and powerful synthetic tools for providing complex molecules in fundamental and applied research due to the easy availability of starting materials, the simplicity and generality of the methods, and the broad tolerance.^[1]^ Continuous tuning of ligands and precatalysts has set the stage for rich applications in the preparation of pharmaceuticals, agrochemicals, as well as advanced multifunctional materials.^[2]^ Among numerous *C*,*N*‐bis(hetero)‐arylated heterocycles, di(hetero)arylated indoles and carbazoles are interesting targets as privileged scaffolds in anticancer research (Figure 1). While 2,3‐bisarylmaleimides bearing *N*‐aryl indole moieties were identified as potent and selective inhibitors of protein kinases C (PKC),^[3]^ a simple *N*‐(*ortho*‐anisyl)‐3‐biphenylindole shows lower micromolar inhibition of the hedgehog signaling pathway,^[4]^ which represents a novel therapeutic principle in the treatment of certain cancers. In addition, *N*‐phenylindolylanilines were found to inhibit the proliferation of HCT‐116 human colon carcinoma cells at nanomolar concentrations.^[5]^ Non‐canonical DNA i‐motif selective ligands based on *N*‐aryl‐3‐triazolyl carbazole have just recently been shown to modulate the transcription of cellular oncogenes.^[6]^

**Figure 1:**
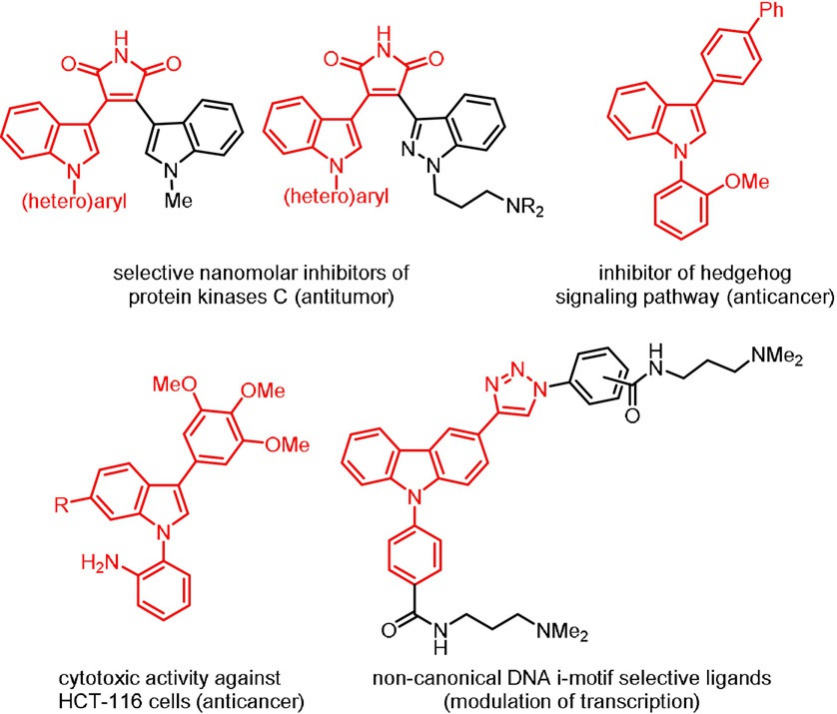
*C*,*N*‐Di(hetero)arylated indoles and carbazoles with considerable anticancer activity.

The contemporary increasing relevance of sustainability and environmental awareness has posed the challenge of high catalyst efficiency and efficacy. Therefore, the catalyst economical use of a single metal catalyst for several organometallic catalytic processes in a single reaction vessel as a one‐pot sequence enables simultaneously operational simplicity, generation of molecular complexity as well as saving of resources.^[7]^ Implementation of one catalyst for two or more mechanistically related reactions leads to sequential or tandem catalysis.^[8]^ Indeed, by the nature of the sequences distinctions are made between metal‐catalyzed domino, tandem or cascade processes.^[9]^ Based upon the concept of sequentially metal‐catalyzed processes employing simple starting materials structural complexity is quickly reached for diversity‐oriented synthesis of functional molecules.^[10]^ Identifying suitable catalysts for concatenation of different reactions in a one‐pot fashion as well as determination of matching reaction conditions remains a major challenge in devising highly practical sequentially catalyzed processes.[^7c^, ^11^] Herein, we communicate our first findings on a novel sequentially Pd‐catalyzed consecutive multicomponent synthesis of *C*,*N*‐diaryl substituted heterocycles, for example, phenothiazines, carbazoles and indoles.

Conceptually, this novel one‐pot approach relies on the Pd‐catalyzed Suzuki coupling of NH‐bearing heteroaryl bromides with (hetero)arylboronic acids or esters, where the catalyst source en route catalyzes a Buchwald‐Hartwig amination without further catalyst addition. Based upon the general interest in diversely substituted phenothiazines potent versatile donor units in diverse organic electronics^[12]^ for application in organic light‐emitting diodes,^[13]^ organic photovoltaics,^[14]^ and as photoredox catalysts^[15]^ we were inspired to devise a consecutive three‐component synthesis of 3,10‐diaryl 10*H*‐phenothiazines by sequential arylation with boronates and arylhalides. According to our findings *N*‐unsubstituted 3‐bromo phenothiazines uneventfully undergo Suzuki arylations with aryl boronic acids in boiling aqueous DME or 1,4‐dioxane.^[16]^

For preventing undesired side reactions, such as homocoupling in the first reaction step, we reasoned that Suzuki coupling should be performed prior to Buchwald–Hartwig amination. By choosing cesium fluoride as a weak base for the Suzuki step, deprotonation of the NH‐bond and the resulting homocoupling of the 3‐bromo‐10*H*‐phenothiazine (**1**) can be efficiently excluded. For Buchwald‐Hartwig reactions alkoxide bases are common^[17]^ for deprotonation of the aryl‐palladium‐amine complex.^[18]^ In particular, NaO*t*Bu has been shown to be appropriate for primary and secondary aliphatic and aromatic amines and phenothiazines.^[19]^ As a corollary, water‐free conditions for the Suzuki coupling must be ensured since trace amounts of water will convert any strong base into relatively weak hydroxides, reducing the efficiency in the Buchwald‐Hartwig reaction of secondary amines.^[18]^

After a thorough optimization of this novel sequentially Pd‐catalyzed one‐pot arylation‐amination process (for details see Supporting Information), 3‐bromo‐10*H*‐phenothiazine (**1**) and arylboronic acids and esters **2** can be transformed in the presence of catalytic amounts of Pd(dba)_2_ and [*t*Bu_3_PH]BF_4_ and cesium fluoride as a base at 120 °C for 16 h, with subsequent addition of aryl bromide **3** and NaO*t*Bu to give 3,10‐diaryl 10*H*‐phenothiazines **4** in moderate to very good yields (Scheme 1).

**Scheme 1:**
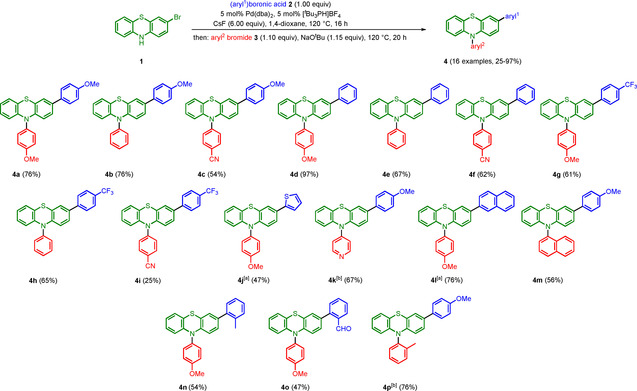
Sequentially Pd‐catalyzed arylation‐amination consecutive three‐component synthesis of 3,10‐diaryl 10*H*‐phenothiazines **4** (^[a]^ aryl^1^‐B(pin) was used.^[b]^ aryl^2^ iodide was used.).

The scope of arylboronic acids **2** in the arylation step is reasonably broad ranging from electron‐donating to electron‐withdrawing groups. Likewise, aryl bromides **3** bearing electron‐withdrawing to electron‐donating groups are equally well tolerated in the concluding amination step. Besides heterocyclic also sterically demanding substituents can be successfully employed.

Encouraged by the straightforward one‐pot synthesis of 3,10‐diaryl 10*H*‐phenothiazines, *N*‐aryl indoles and carbazoles as privileged heterocycles in hole transport molecules for organic light‐emitting diodes (OLEDs) and as biologically active compounds represent attractive targets.[^13a^, ^20^] Especially, aryl carbazoles have recently received great interest as materials in organic electroluminescence devices.^[21]^ To our surprise and to the best of our knowledge, a direct, universal and high yielding one‐pot access to 3,9‐diaryl 9*H*‐carbazoles starting from 3‐bromo‐9*H*‐carbazole (**5**) has not been established yet.

Employing NaO*t*Bu as a base in the amination step only leads to trace amounts of products of the targeted products. However, applying Watanabe's conditions for the arylation of azoles with potassium carbonate as a base^[20]^ set the stage for concatening Suzuki and Buchwald‐Hartwig coupling. With this modification in hand, starting from 3‐bromo 9*H*‐carbazole (**5**) or 3‐bromo 1*H*‐indole (**6**) the consecutive three‐component coupling‐amination synthesis of 3,9‐diaryl 9*H*‐carbazoles **7** and 1,5‐diaryl 1*H*‐indoles **8** was illustrated in 20 preparative examples, furnishing the target compounds in moderate to very good yields (Scheme 2). As for the one‐pot synthesis of 3,10‐diaryl 10*H*‐phenothiazines **4** (Scheme 1) various electronically diverse arylboronic acid (ester) **2** and aryl bromides **3** were well tolerated also for the one‐pot syntheses of systems **7** and **8**. Likewise, heterocyclic and sterically demanding substituents can be successfully introduced via this sequence.

**Scheme 2:**
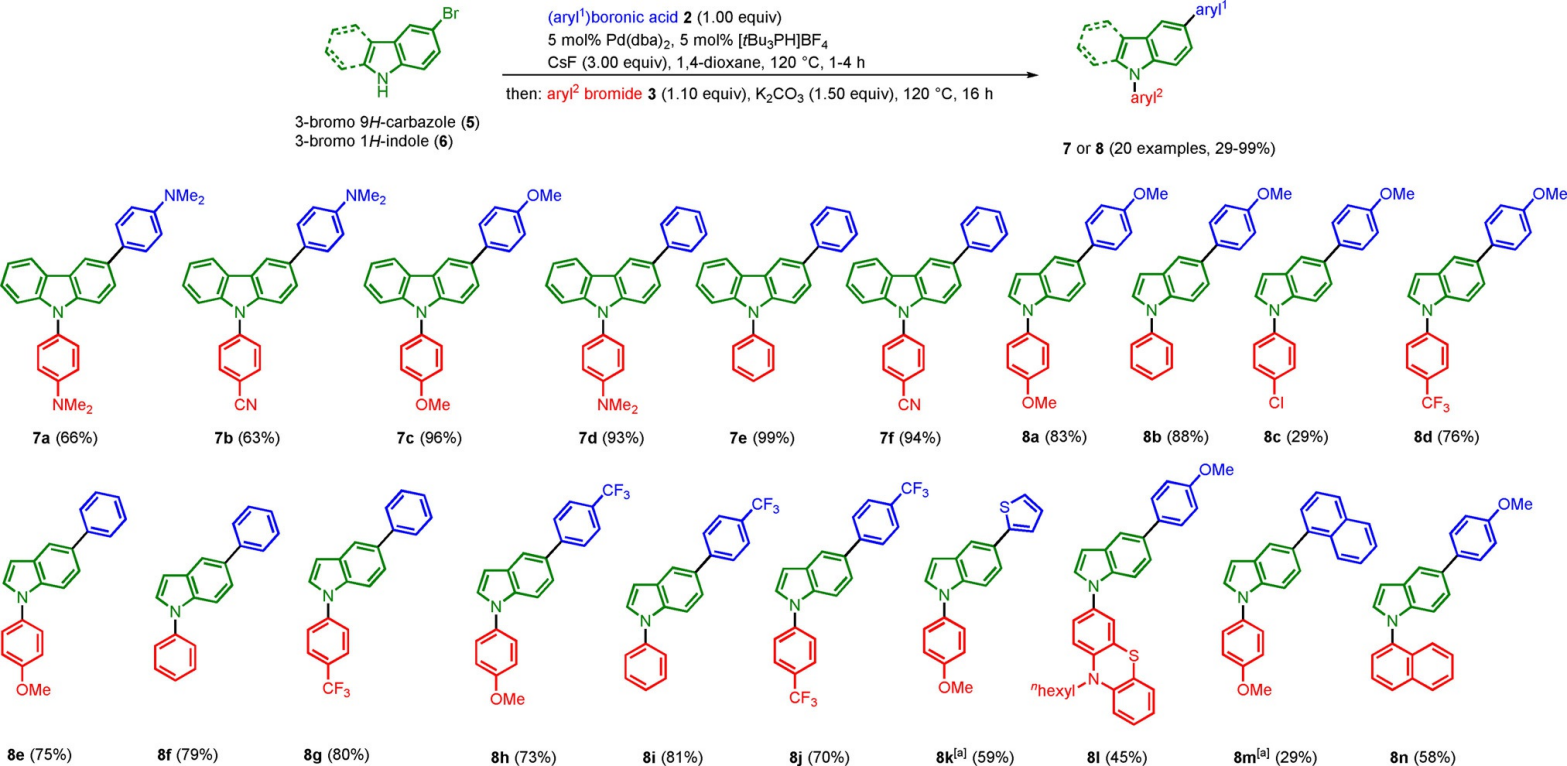
Sequentially Pd‐catalyzed arylation‐amination consecutive three‐component synthesis of 3,9‐diaryl 9*H*‐carbazoles **7** and 1,5‐diaryl 1*H*‐indoles **8**. (^[a]^ aryl^1^‐B(pin) was used.).

In summary, we have successfully developed and disclosed a novel, highly practical, and diversity‐oriented access to *C*,*N*‐biarylfunctionalized heterocycles based upon a sequentially Pd‐catalyzed arylation–amination sequence. A remarkable feature of this novel one‐pot process is the sequential use of the palladium catalyst for both the Suzuki arylation and the Buchwald–Hartwig amination without further catalyst addition. In this illustration the targeted diarylated phenothiazines, carbazoles, and indoles are obtained in good yields with a high tolerance of functional groups. Bearing in mind the huge pool of commercially available or easily accessible starting materials, this methodology can be most favorably applied on a broad scale for preparing libraries of potentially interesting biologically active and/or functional materials. Based upon our interest in tunable phenothiazines systematic studies on structure‐property relationships and the electronic structure of 3,10‐diaryl 10*H*‐phenothiazines are currently underway.

## Experimental Section

### Suzuki Buchwald–Hartwig one‐pot synthesis of 10‐(4‐methoxyphenyl)‐3‐phenyl‐10*H*‐phenothiazine (4 d) (typical procedure)

Under argon in a Schlenk tube with a magnetic stir bar 3‐bromo‐10*H*‐phenothiazine (**1**) (138 mg, 0.500 mmol), phenylboronic acid (**2 c**) (61.0 mg, 0.500 mmol), Pd(dba)_2_ (14 mg, 5.0 mol %), tri‐*tert*‐butylphosphane tetrafluoroborate (8 mg, 5.0 mol %) and cesium fluoride (455 mg, 3.00 mmol) were dissolved in dry 1,4‐dioxane (3 mL). After purging with argon for 5 min, the reaction mixture was stirred at 120 °C (oil bath temperature) for 16 h. After cooling to room temp, 1‐bromo‐4‐methoxybenzene (**3 b**) (103 mg, 0.550 mmol) and sodium *tert*‐butoxide (55.0 mg, 0.575 mmol) were added and the reaction mixture was purged with argon for 5 min. Then, the reaction mixture was stirred at 120 °C (oil bath temperature) for 20 h. After cooling to room temp aqueous work up and extraction with dichloromethane the residue was purified by flash chromatography (*n*‐hexane/acetone 50:1) on silica gel to give compound **4 d** (185 mg, 0.485 mmol, 97 %) as yellow crystals.

M.p. 169 °C. ^1^H NMR (600 MHz, [D_6_]acetone): *δ* 3.93 (s, 3 H), 6.22 (dd, ^3^ *J*=8.3 Hz, ^4^ *J*=1.2 Hz, 1 H), 6.27 (d, ^3^ *J*=8.6 Hz, 1 H), 6.83 (td, ^3^ *J*=7.5 Hz, ^4^ *J*=1.3 Hz, 1 H), 6.91 (ddd, ^3^ *J*=8.3 Hz, 7.4 Hz, ^4^ *J*=1.6 Hz, 1 H), 7.03 (dd, ^3^ *J*=7.5 Hz, ^4^ *J*=1.6 Hz, 1 H), 7.19 (dd, ^3^ *J*=8.5 Hz, ^4^ *J*=2.2 Hz, 1 H), 7.23–7.27 (m, 2 H), 7.27–7.32 (m, 2 H), 7.36–7.42 (m, 4 H), 7.57 (dd, ^3^ *J*=8.4 Hz, ^4^ *J*=1.3 Hz, 2 H). ^13^C NMR (150 MHz, [D_6_]acetone): *δ* 55.9 (CH_3_), 116.6 (CH), 116.9 (CH), 117.0 (CH), 120.1 (C_quat)_, 120.9 (C_quat_), 123.3 (CH), 125.4 (CH), 126.3 (CH), 126.9 (CH), 127.4 (CH), 127.9 (CH), 127.9 (CH), 129.7 (CH), 133.0 (CH), 133.9 (C_quat_), 136.0 (C_quat_), 140.4 (C_quat_), 144.8 (C_quat_), 145.3 (C_quat_), 160.6 (C_quat_). EI MS (70 eV): *m*/*z* (%): 382 (26), 381 ([M]^+^, 100), 366 ([C_24_H_16_NOS]^+^, 15), 274 ([C_18_H_12_NS]^+^, 13), 272 (10), 191 (11), 152 (10). Anal. calcd for C_25_H_19_NOS [381.5]: C 78.71, H 5.02, N 3.67, S 8.40; Found: C 78.51, H 5.06, N 3.53, S 8.69.

### Suzuki Buchwald–Hartwig one‐pot synthesis of 3,9‐bis(4‐methoxyphenyl)‐9*H*‐carbazole (7 c) (typical procedure)

Under argon in a Schlenk tube with a magnetic stir bar 3‐bromo‐9*H*‐carbazole (**5**) (123 mg, 0.500 mmol), (4‐methoxyphenyl)boronic acid (**2 b**) (76.0 mg, 0.500 mmol), Pd(dba)_2_ (14 mg, 5.0 mol %), tri‐*tert*‐butylphosphane tetrafluoroborate (8 mg, 5.0 mol %) and cesium fluoride (227 mg, 1.50 mmol) were dissolved in dry 1,4‐dioxane (3 mL). After purging with argon for 5 min, the reaction mixture was stirred at 120 °C (oil bath temperature) for 4 h. After cooling to room temp, 1‐bromo‐4‐methoxybenzene (**3 b**) (103 mg, 0.550 mmol) and potassium carbonate (207 mg, 1.50 mmol) were added and the reaction mixture was purged with argon for 5 min. Then, the reaction mixture was stirred at 120 °C (oil bath temperature) for 20 h. After cooling to room temp aqueous work up and extraction with dichloromethane the residue was purified by flash chromatography (*n*‐hexane/acetone 50:1) on silica gel to give compound **7 c** (182 mg, 0.479 mmol, 96 %) as colorless crystals.

M.p. 122 °C. ^1^H NMR (600 MHz, [D_6_]acetone): *δ* 3.70 (s, 3 H), 3.79 (s, 3 H), 6.86–6.93 (m, 2 H), 7.06–7.10 (m, 2 H), 7.11–7.15 (m, 1 H), 7.19 (dd, ^3^ *J*=14.9 Hz, 8.3 Hz, 2 H), 7.27 (ddd, ^3^ *J*=8.2 Hz, 7.0 Hz, ^4^ *J*=1.2 Hz, 1 H), 7.34–7.41 (m, 2 H), 7.51 (dd, ^3^ *J*=8.5 Hz, ^4^ *J*=1.8 Hz, 1 H), 7.53–7.56 (m, 2 H), 8.14 (d, ^3^ *J*=7.8 Hz, 1 H), 8.30 (d, ^4^ *J*=1.8 Hz, 1 H). ^13^C NMR (150 MHz, [D_6_]acetone): *δ* 55.6 (CH_3_), 56.0 (CH_3_), 110.5 (CH), 110.7 (CH), 115.1 (CH), 116.1 (CH), 118.9 (CH), 120.6 (CH), 121.3 (CH), 124.1 (C_quat_), 124.6 (C_quat_), 125.8 (CH), 127.0 (CH), 128.8 (CH), 129.3 (CH), 130.9 (C_quat_), 133.8 (C_quat_), 135.1 (C_quat_), 141.3 (C_quat_), 142.6 (C_quat_), 159.8 (C_quat_), 160.1 (C_quat_). EI MS (70 eV): *m*/*z* (%): 380 (27), 379 ([M]^+^, 100), 365 (15), 364 ([C_25_H_18_NO_2_]^+^, 59), 293 (10), 292 (14), 190 (20), 168 (10), 146 (14). Anal. calcd for C_26_H_21_NO_2_ [379.5]: C 82.30, H 5.58, N 3.69; Found: C 82.06, H 5.49, N 3.52.

### Suzuki Buchwald–Hartwig one‐pot synthesis of 5‐(4‐methoxyphenyl)‐1‐phenyl‐1*H*‐indole (8 b) (typical procedure)

Under argon in a Schlenk tube with a magnetic stir bar 5‐bromo‐1*H*‐indole (**6**) (98.0 mg, 0.500 mmol), (4‐methoxyphenyl)boronic acid (**2 b**) (0.500 mmol), Pd(dba)_2_ (14 mg, 5.0 mol %), tri‐*tert*‐butylphosphane tetrafluoroborate (8 mg, 5.0 mol %) and cesium fluoride (227 mg, 1.50 mmol) were dissolved in dry 1,4‐dioxane (3 mL). After purging with argon for 5 min, the reaction mixture was stirred at 120 °C (oil bath temperature) for 1 h. After cooling to room temp, bromobenzene (**3 c**) (86.0 mg, 0.550 mmol) and potassium carbonate (207 mg, 1.50 mmol) and the reaction mixture was purged with argon for 5 min. Then, the reaction mixture was stirred at 120 °C (oil bath temperature) for 20 h. After cooling to room temp aqueous work up and extraction with dichloromethane the residue was purified by flash chromatography (*n*‐hexane/acetone 20:1) on silica gel and by recrystallization from *n*‐hexane to give compound **8 b** (132 mg, 0.441 mmol, 88 %) as colorless crystals.

M.p. 130 °C. ^1^H NMR (600 MHz, [D_6_]acetone): *δ* 3.84 (s, 3 H), 6.74 (d, ^3^ *J*=3.1 Hz, 1 H), 7.02 (d, ^3^ *J*=8.5 Hz, 2 H), 7.42 (t, ^3^ *J*=6.7 Hz, 1 H), 7.47 (td, ^3^ *J*=8.6 Hz, 1 H), 7.56 (d, ^3^ *J*=3.2 Hz, 1 H), 7.58–7.65 (m, 7 H), 7.87 (d, ^4^ *J*=1.7 Hz, 1 H). ^13^C NMR (150 MHz, [D_6_]acetone): *δ* 55.6 (CH_3_), 104.8 (CH), 111.5 (CH), 115.0 (CH), 119.6 (CH), 122.5 (CH), 124.8 (CH), 127.3 (CH), 128.8 (CH), 129.49 (CH), 130.2 (CH), 131.2 (C_quat_), 134.3 (C_quat_), 135.4 (C_quat_), 135.8 (C_quat_), 140.7 (C_quat_), 159.7 (C_quat_). EI MS (70 eV): *m*/*z* (%): 300 (23), 299 ([M]^+^, 100), 285 (15), 284 ([C_20_H_14_NO]^+^, 67), 256 ([C_19_H_14_N]^+^, 25), 254 (10), 152 (11), 150 (18). Anal. calcd for C_21_H_17_NO [299.4]: C 84.25, H 5.72, N 4.68; Found: C 84.37, H 5.80, N 4.66.

## Conflict of interest

The authors declare no conflict of interest.

## Supporting information

As a service to our authors and readers, this journal provides supporting information supplied by the authors. Such materials are peer reviewed and may be re‐organized for online delivery, but are not copy‐edited or typeset. Technical support issues arising from supporting information (other than missing files) should be addressed to the authors.

Supplementary
