# Supplementary material for: Concatenating Suzuki Arylation and Buchwald–Hartwig Amination by A Sequentially Pd‐Catalyzed One‐Pot Process—Consecutive Three‐Component Synthesis of C,N‐Diarylated Heterocycles
Source: Chemistry. 2020 Oct 19;26(66):15130–4. doi: 10.1002/chem.202003837 (PMC7756286; doi:10.1002/chem.202003837)
Supplement: Supplementary file 1 — Supplementary [file CHEM-26-15130-s001.pdf]

Chemistry–A European Journal

Supporting Information

Concatenating Suzuki Arylation and Buchwald–Hartwig Amination by A Sequentially Pd-Catalyzed One-Pot Process—Consecutive Three-Component Synthesis of *C,N*-Diarylated Heterocycles

Laura Mayer, Regina Kohlbecher, and Thomas J. J. Müller*[a]

Supporting Information

Table of contents

1. General considerations 2

2. Optimization 3

3. General Procedures for the One-pot coupling-amination-sequence 5

General procedure 1 (GP1) for the synthesis of the 3,10-diaryl 10*H*-phenothiazines **4** 5

Spectroscopic data of 3,10-diaryl phenothiazines **4** 9

General procedure 2 (GP2) for the synthesis of the 3,9-diaryl 9*H*-carbazoles **7** 17

Spectroscopic data of 3,9-diaryl carbazoles **7** 19

General procedure 3 (GP3) for the synthesis of the 1,5-diaryl 1*H*-indoles **8** 22

Spectroscopic data of 1,5-diaryl indoles **8** 26

4. NMR spectra 33

4.1 ^{1}H and ^{13}C NMR spectra of 3,10-diaryl phenothiazines **4** 33

4.2 ^{1}H and ^{13}C NMR spectra of 3,9-diaryl carbazoles **7** 49

4.3 ^{1}H and ^{13}C NMR spectra of 1,5-diaryl indoles **8** 55

Literature 70

1. General considerations

All reactions were carried out in oven dried Schlenk glassware using septa and syringes under nitrogen atmosphere. 3-bromo-10*H*-phenothiazine **1**,[1] 3-bromo-9*H*-carbazole **5**,[2] 4,4,5,5-tetramethyl-2-(thiophen-2-yl)-1,3,2-dioxaborolane **2e**,[3] 4,4,5,5-tetramethyl-2-(naphthalen-1-yl)-1,3,2-dioxaborolane **2f**,[4] 3-Bromo-10-hexyl-10*H*-phenothiazine **3i**,[5] were known compounds and were synthesized according to literature procedures. The other reagents and catalyst were purchased reagent-grade and used without purification. Dry solvents were dried by a solvent purification system.

The purification of the products was performed on silica gel 60 M (0.04-0.063 mm) from MACHEREY-NAGEL GmbH & Co. KG using flash technique under pressure of 2 bar. The crude mixtures were absorbed on Celite® 545 form Carl Roth GmbH & Co. KG before chromatographic purification.

The reaction progress was monitored qualitatively using TLC Silica gel 60 F234 aluminum sheets obtained from MACHEREY-NAGEL GmbH & Co. KG. The spots were detected with UV light at 254 and 366 nm.

^{1}H, ^{13}C and 135-DEPT ^{13}C NMR spectra were recorded on Bruker AVIII-300 and AVIII-600. Acetone-d_6 and CD_2Cl_2 were used as deuterated solvents. The resonances of the solvents were locked as internal standard (acetone-d_6: ^{1}H δ 2.05, ^{13}C δ 29.84, 206.26; CD_2Cl_2: ^{1}H δ 5.32, ^{13}C δ 54.00). The multiplicities of the signals were abbreviated as follows: s: singlet; d: doublet; t: triplet; q: quartet; dd: doublet of doublet; td: triplet of doublets; ddd: doublet of doublet of doublets; m: multiplet. The type of carbon atoms was determined on the basis of 135-DEPT ^{13}C NMR spectra. For the description of the ^{13}C NMR spectra primary carbon atoms are abbreviated with CH_3, secondary carbon atoms with CH_2, tertiary carbon atoms with CH and quaternary carbon atoms with C_{quat}.

EI mass spectra were measured on Finnigan MAT TSQ 7000.

IR spectra were obtained on Shimadzu IR Affinity-1 which works with the attenuated total reflection (ATR) method. The intensity of signals is abbreviated as follows: s (strong), m (medium), w (weak).

The melting points (uncorrected) were measured on Büchi Melting Point B-540.

Combustion analyses were carried out on Perkin Elmer Series II Analyser 2400 in the micro analytical laboratory of the Institut für Pharmazeutische und Medizinische Chemie der Heinrich-Heine-Universität Düsseldorf.

2. Optimization

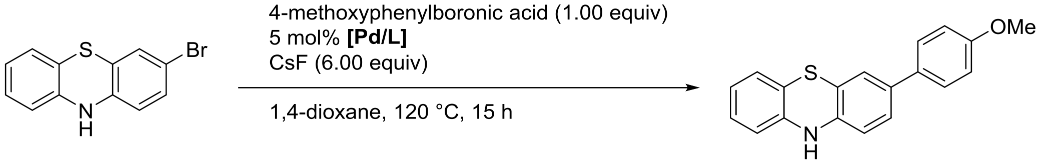

Table S1: Optimization of the catalyst system for the synthesis of 3-(4-methoxyphenyl)-10*H*-phenothiazine.

entry	[Pd]	ligand	yield [%][a]
1	$Pd(PPh_3)_4$		75
2	$PdCl_2$	dppf	69
3	$PdCl_2$	$[^tBu_3PH]BF_4$	45
4	$PdCl_2$	SPhos	31
5	$PdCl_2$	PCy_3	37
6	$Pd(dba)_2$	dppf	75
7	$Pd(dba)_2$	$[^tBu_3PH]BF_4$	71
**8**	**$Pd(dba)_2$**	**SPhos**	**91**
9[b]	$Pd(dba)_2$	SPhos	72
10	$Pd(OAc)_2$	$[^tBu_3PH]BF_4$	33

[a] Yields after flash chromatography on silica gel. [b] 3.00 equiv of CsF were used.

Table S2: Optimization of the catalyst system for the synthesis of 3,10-Bis(4-methoxyphenyl)-10*H*-phenothiazine.

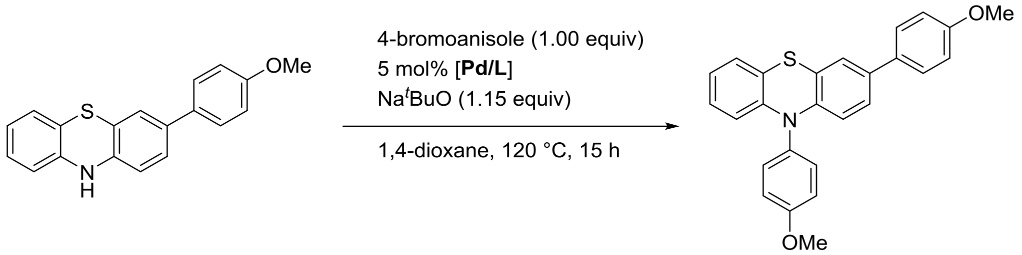

entry	[Pd]	ligand	yield [%][a]
1	$Pd(PPh_3)_4$		42
2	$Pd(dba)_2$	Dppf	24
**3**	**$Pd(dba)_2$**	**$[^tBu_3PH]BF_4$**	**78**
**4**	**$Pd(dba)_2$**	**SPhos**	**78**

[a] Yields after flash chromatography on silica gel.

Table S3: Optimization of the coupling-amination-sequence.

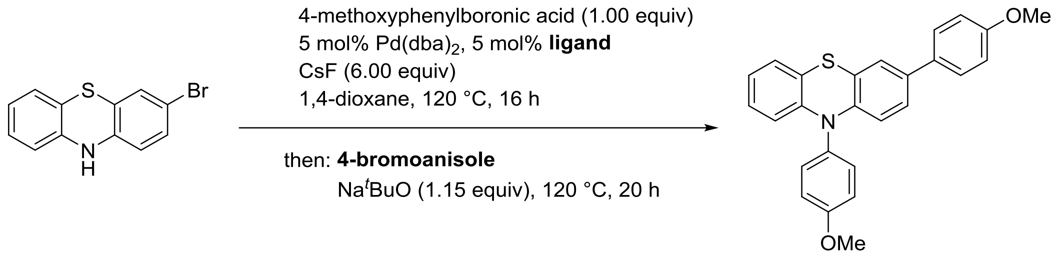

entry	ligand	4-bromoanisole [eq.]	yield [%][a]
1	SPhos	1.0	56
**2**	**SPhos**	**1.1**	**76**
**3**	**[^{t}Bu$_3$PH]BF$_4$**	**1.1**	**76**

[a] Yields after flash chromatography on silica gel.

3. General Procedures for the One-pot coupling-amination-sequence

General procedure 1 (GP1) for the synthesis of the 3,10-diaryl 10*H*-phenothiazines 4

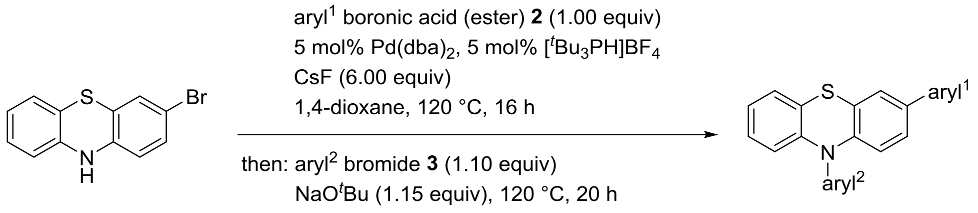

1 **4**

Under argon in a Schlenk tube with magnetic stir bar 3-bromo-10*H*-phenothiazine (**1**) (138 mg, 0.500 mmol), aryl boronic acid or ester **2** (0.500 mmol), Pd(dba)$_2$ (14 mg, 5.0 mol%), tri-*tert*-butylphosphane tetrafluoroborate (8 mg, 5.0 mol%) and cesium fluoride (455 mg, 3.00 mmol) were dissolved in dry 1,4-dioxane (3 mL). The solution was degassed with argon for 5 min. Then, the reaction mixture was stirred at 120 °C (oil bath temperature) for 16 h. After cooling to room temperature, aryl bromide **3** (0.550 mmol) and sodium *tert*-butoxide (55.0 mg, 0.575 mmol) were added and the reaction mixture was degassed with argon for 5 min. Then, the reaction mixture was stirred at 120 °C (oil bath temperature) for 20 h. After cooling to room temp deionized water (50 mL), saturated Na_2SO_3 solution (15 mL) and dichloromethane (50 mL) were successively added. The aqueous phase was extracted with dichloromethane (3 x 10 mL). The combined organic phases were dried with anhydrous magnesium sulfate and the solvents were removed in vacuo. The residue was purified by flash chromatography on silica gel and by recrystallization from *n*-hexane/acetone to give compound **4** as a solid.

Table S4: Experimental details for the synthesis of 3,10-diaryl 10*H*-phenothiazines **4**.

entry	arylboronic acid or ester **2**	aryl bromide **3**	yield of product **4**[a]
**1**	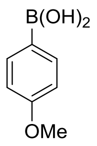 **2b**, 76 mg	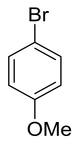 **3b**, 103 mg	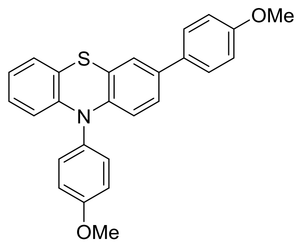 **4a**, 157 mg (76%)
**2**	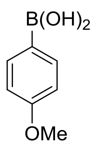 **2b**, 76 mg	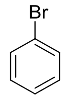 **3c**, 86 mg	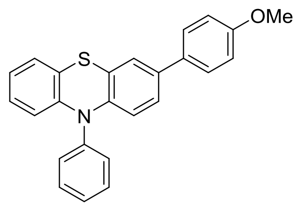 **4b**, 144 mg (76%)

[a] Yields after flash chromatography on silica gel.

Table S4: Experimental details for the synthesis of 3,10-diaryl 10*H*-phenothiazines **4**.

entry	arylboronic acid or ester **2**	aryl bromide **3**	yield of product **4**
**3**	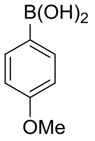 **2b**, 76 mg	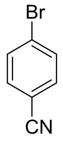 **3f**, 100 mg	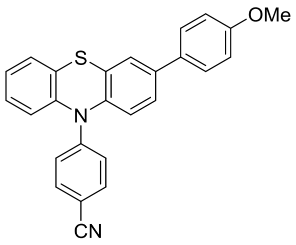 **4c**, 93 mg (54%)
**4**	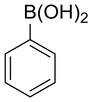 **2c**, 61 mg	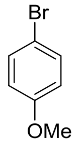 **3b**, 103 mg	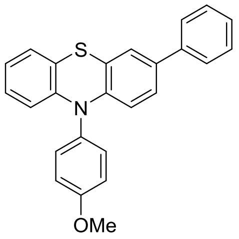 **4d**, 185 mg (97%)
**5**	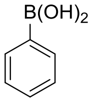 **2c**, 61 mg	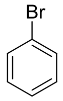 **3c**, 86 mg	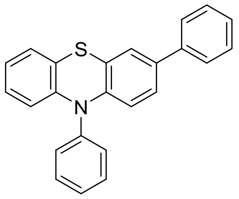 **4e**, 118 mg (67%)
**6**	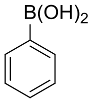 **2c**, 61 mg	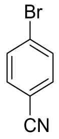 **3e**, 100 mg	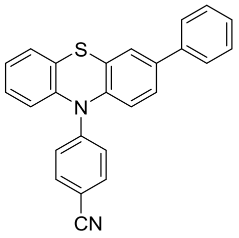 **4f**, 117 mg (62%)
**7**	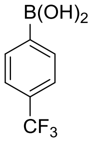 **2d**, 95 mg	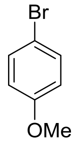 **3b**, 103 mg	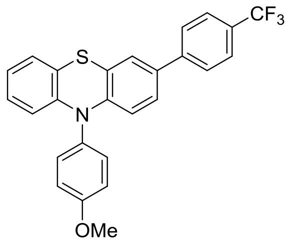 **4g**, 138 mg (61%)
**8**	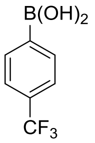 **2d**, 95 mg	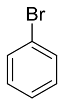 **3c**, 86 mg	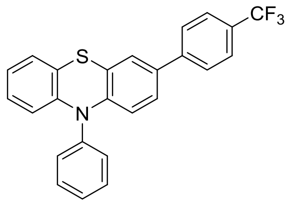 **4h**, 137 mg (65%)

[a] Yields after flash chromatography on silica gel.

Table S4: Experimental details for the synthesis of 3,10-diaryl 10*H*-phenothiazines **4**.

entry	arylboronic acid or ester **2**	aryl bromide **3**	yield of product **4**
**9**	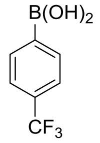 **2d**, 95 mg	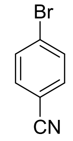 **3f**, 100 mg	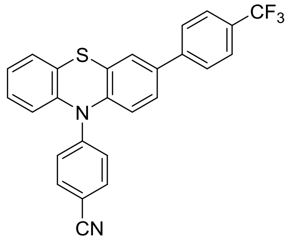 **4i**, 105 mg (47%)
**10**	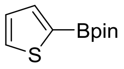 **2e**, 105 mg	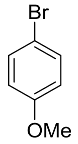 **3b**, 103 mg	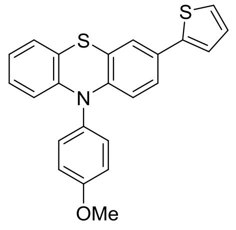 **4j**, 72 mg (37%)
**11**	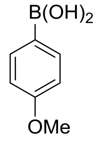 **2b**, 76 mg	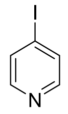 **3g**, 113 mg	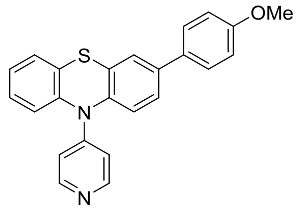 **4k**, 128 mg (67%)
**12**	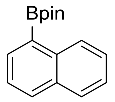 **2f**, 127 mg	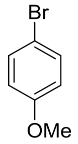 **3b**, 103 mg	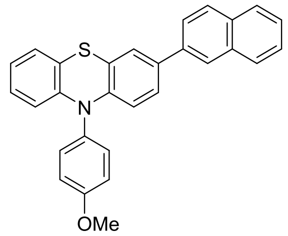 **4l**, 164 mg (76%)
**13**	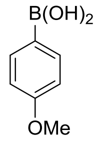 **2b**, 76 mg	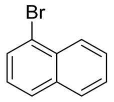 **3h**, 114 mg	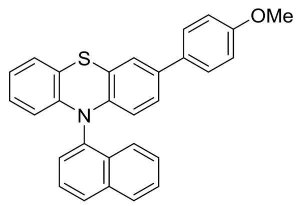 **4m**, 121 mg (56%)
**14**	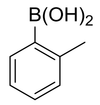 **2g**, 68 mg	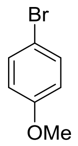 **3b**, 103 mg	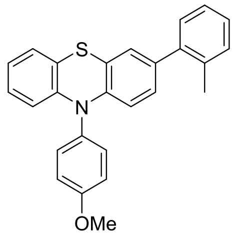

4n, 107 mg (37%)

[a] Yields after flash chromatography on silica gel.

Table S4: Experimental details for the synthesis of 3,10-diaryl 10*H*-phenothiazines **4**.

entry	arylboronic acid or ester **2**	aryl bromide **3**	yield of product **4**
**15**	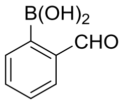 **2h**, 75 mg	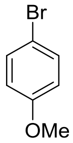 **3b**, 103 mg	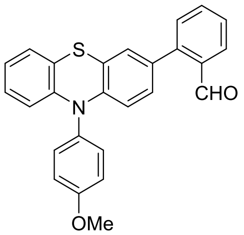 **4o**, 105 mg (47%)
**16**	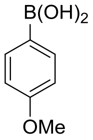 **2b**, 76 mg	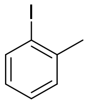 **3b**, 120 mg	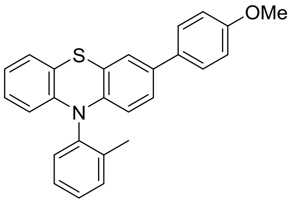 **4p**, 164 mg (76%)

[a] Yields after flash chromatography on silica gel.

Spectroscopic data of 3,10-diaryl phenothiazines 4

3,10-Bis(4-methoxyphenyl)-10*H*-phenothiazine (4a)

The synthesis was performed by GP1. After chromatography on silica gel (*n*-hexane/acetone 50:1) and recrystallization from *n*-hexane/acetone, **4a** (157 mg, 0.382 mmol, 76%) was isolated as yellow crystals. Mp 160 °C. ^{1}H NMR (300 MHz, acetone-d$_6$): δ 3.81 (s, 3 H), 3.92 (s, 3 H), 6.18-6.27 (m, 2 H), 6.79-6.93 (m, 2 H), 6.90-7.02 (m, 2 H), 7.00-7.05 (m, 1 H), 7.13 (dd, ^3J = 8.6 Hz, ^4J = 2.2 Hz, 1 H), 7.20 7.27 (m, 3 H), 7.31-7.43 (m, 2 H), 7.45-7.54 (m, 2 H). ^{13}C NMR (125 MHz, acetone-d$_6$): δ 55.6 (CH_3), 55.9 (CH_3), 115.1 (CH), 116.6 (CH), 116.9 (CH), 120.1 (C_{quat}), 120.9 (C_{quat}), 123.2 (CH), 124.9 (CH), 125.8 (CH), 127.3 (CH), 127.9 (CH), 128.0 (CH), 132.8 (C_{quat}), 133.0 (CH), 134.0 (C_{quat}), 135.9 (C_{quat}), 144.2 (C_{quat}), 145.4 (C_{quat}), 160.1 (C_{quat}), 160.5 (C_{quat}). EI MS (70 eV): *m*/*z* (%): 412 (28), 411 ([M]$^+$, 100), 396 ([$C_{25}H_{18}NO_2S$]$^+$, 28), 206 (24). IR: $\tilde{\nu}$ [cm^{-1}] = 3036 (w). 2953 (w), 2907 (w), 2833 (w), 1578 (w), 1508 (m), 1491 (m), 1460 (s), 1439 (m), 1425 (w), 1389 (w), 1308 (m), 1292 (m), 1283 (m), 1238 (s), 1184 (m), 1177 (m), 1165 (m), 1152 (w), 1130 (w), 1115 (m), 1080 (w), 1049 (m), 1024 (m), 1009 (w), 912 (w), 883 (w), 835 (s), 804 (s), 795 (m), 768 (w), 746 (s), 727 (m), 704 (w), 679 (w), 646 (m). Anal. calcd. for $C_{26}H_{21}NO_2S$ [411.5]: C 75.89, H 5.14, N 3.40, S 7.79; Found: C 75.82, H 5.06, N 3.43, S 7.77.

3-(4-Methoxyphenyl)-10-phenyl-10*H*-phenothiazine (4b)

The synthesis was performed by GP1. After chromatography on silica gel (*n*-hexane/acetone 50:1) and recrystallization from *n*-hexane/acetone, **4b** (144 mg, 0.377 mmol, 76%) was isolated as yellow crystals. Mp 209 °C. ^{1}H NMR (300 MHz, acetone-d$_6$): δ. 3.81 (s, 3 H), 6.20-6.28 (m, 2 H), 6.82-6.93 (m, 2 H), 6.94-7.00 (m, 2 H), 7.04-7.09 (m, 1 H), 7.15 (dd,^3J = 8.6, ^4J = 2.2 Hz, 1 H), 7.28 (d, ^4J = 2.1 Hz, 1 H), 7.42-7.53 (m, 4 H), 7.55-7.62 (m, 1 H), 7.66-7.75 (m, 2 H). ^{13}C NMR (75 MHz, acetone-d$_6$): δ 55.6 (CH_3), 115.1 (CH), 117.0 (CH), 117.3 (CH), 120.7 (C_{quat}), 121.4 (C_{quat}), 123.5 (CH), 125.1 (CH), 125.8 (CH), 127.5 (CH), 128.0 (CH), 128.1 (CH), 129.3 (CH), 131.6 (CH), 131.9 (CH), 132.8 (C_{quat}), 136.1 (C_{quat}), 141.9 (C_{quat}), 143.8 (C_{quat}), 145.0 (C_{quat}), 160.2(C_{quat}). EI MS (70 eV): *m*/*z* (%): 382 (25), 381 ([M]$^+$, 100), 366 ([$C_{24}H_{16}NOS$]$^+$, 27), 338 (10), 304 (14), 261 (14), 191 (19). IR: $\tilde{\nu}$ [cm^{-1}] = 3645 (w), 3069 (w), 3057 (w), 2990 (w), 2968 (w), 2893 (w), 2839 (w), 2818 (w), 1605 (w), 1591 (m), 1578 (m), 1518 (w), 1491 (m), 1460 (s), 1437 (m), 1422 (m), 1387 (m), 1362 (w), 1339 (w), 1304 (m), 1283 (m), 1254 (s), 1240 (s), 1153 (m), 1125 (w), 1113 (m), 1047 (s), 1022 (m), 1005 (m), 961 (w), 937 (w), 925 (w), 903 (w), 876 (m), 837 (m), 808 (s), 781 (w), 746 (s), 719 (m), 698 (m), 662 (m), 633 (s), 621 (m). Anal. calcd. for $C_{25}H_{19}NOS$ [381.5]: C 78.71, H 5.02, N 3.67, S 8.40, Found: C 78.49, H 5.11, N 3.53, S 8.35.

4-(3-(4-Methoxyphenyl)-10*H*-phenothiazin-10-yl)benzonitrile (4c)

The synthesis was performed by GP1. After chromatography on silica gel (*n*-hexane/acetone 50:1) and recrystallization from *n*-hexane/acetone, **4c** (110 mg, 0.270 mmol, 54%) was isolated as colorless crystals.

Mp 228 °C. ^{1}H NMR (500 MHz, acetone-d$_6$): δ 3.85 (s, 3 H), 7.01-7.05 (m, 2 H), 7.23-7.28 (m, 3 H), 7.29-7.34 (m, 2 H), 7.38 (ddd, ^3J = 8.0 Hz, 7.3 Hz, ^4J = 1.5 Hz, 1 H), 7.50 (dd, ^3J = 7.8 Hz, ^4J = 1.5 Hz, 1 H), 7.59 (dd, ^3J = 8.3 Hz, ^4J = 2.1 Hz, 1 H), 7.62-7.65 (m, 2 H), 7.67-7.71 (m, 3 H). ^{13}C NMR (125 MHz, acetone-d$_6$): δ 55.7 (CH$_3$), 106.1 (C$_{quat}$), 115.3 (CH), 119.6 (CH), 119.6 (C$_{quat}$), 126.1 (CH), 126.3 (CH), 126.6 (CH), 127.0 (CH), 127.0 (CH), 128.6 (CH), 128.8 (CH), 129.5 (CH), 132.3 (C$_{quat}$), 132.6 (C$_{quat}$), 132.9 (C$_{quat}$), 134.7 (CH), 139.7 (C$_{quat}$), 140.9 (C$_{quat}$), 142.4 (C$_{quat}$), 149.7 (C$_{quat}$), 160.8 (C$_{quat}$). EI MS (70 eV): *m*/*z* (%): 407 (28), 406 ([M]$^+$, 100), 391 ([$C_{25}H_{15}N_2OS$]$^+$, 24), 363 ([$C_{25}H_{17}NS$]$^+$, 10), 304 (15), 261 (17), 203 (16). IR: $\tilde{\nu}$ [cm^{-1}] = 3049 (w), 2992 (w), 2961 (w), 2930 (w), 2901 (w), 2837 (w), 1605 (m), 1591 (m), 1520 (w), 1503 (m), 1483 (m), 1466 (m), 1443 (m), 1418 (w), 1387 (w), 1362 (w), 1312 (m), 1290 (m), 1265 (m), 1248 (m), 1238 (m), 1184 (m), 1173 (m), 1126 (m), 1111 (m), 1074 (w), 1065 (w), 1045 (m), 1026 (m), 1007 (w), 984 (w), 943 (w), 920 (w), 897 (m), 866 (w), 847 (m), 820 (s), 808 (m), 781 (w), 768 (m), 756 (s), 721 (w), 700 (w), 681 (m), 667 (w). Anal. calcd. for $C_{26}H_{18}N_2OS$ [406.5]: C 76.82, H 4.46, N 6.89, S 7.89, Found: C 76.56, H 4.49, N 6.72, S 7.78.

10-(4-Methoxyphenyl)-3-phenyl-10*H*-phenothiazine (4d)

The synthesis was performed by GP1. After chromatography on silica gel (*n*-hexane/acetone 50:1), **4d** (185 mg, 0.485 mmol, 97%) was isolated as yellow crystals.

Mp 169 °C. ^{1}H NMR (600 MHz, acetone-d$_6$): δ 3.93 (s, 3 H), 6.22 (dd, ^3J = 8.3 Hz, ^4J = 1.2 Hz, 1 H), 6.27 (d, ^3J = 8.6 Hz, 1 H), 6.83 (td, ^3J = 7.5 Hz, ^4J = 1.3 Hz, 1 H), 6.91 (ddd, ^3J = 8.3 Hz, 7.4 Hz, ^4J = 1.6 Hz, 1 H), 7.03 (dd, ^3J = 7.5 Hz, ^4J = 1.6 Hz, 1 H), 7.19 (dd, ^3J = 8.5 Hz, ^4J = 2.2 Hz, 1 H), 7.23-7.27 (m, 2 H), 7.27-7.32 (m, 2 H), 7.36-7.42 (m, 4 H), 7.57 (dd, ^3J = 8.4 Hz, ^4J = 1.3 Hz, 2 H). ^{13}C NMR (150 MHz, acetone-d$_6$): 55.9 (CH$_3$), 116.6 (CH), 116.9 (CH), 117.0 (CH), 120.1 (C$_{quat}$), 120.9 (C$_{quat}$), 123.3 (CH), 125.4 (CH), 126.3 (CH), 126.9 (CH), 127.4 (CH), 127.9 (CH), 127.9 (CH), 129.7 (CH), 133.0 (CH), 133.9 (C$_{quat}$), 136.0 (C$_{quat}$), 140.4 (C$_{quat}$), 144.8 (C$_{quat}$), 145.3 (C$_{quat}$), 160.6 (C$_{quat}$). EI MS (70 eV): *m*/*z* (%): 382 (26), 381 ([M]$^+$, 100), 366 ([$C_{24}H_{16}NOS$]$^+$, 15), 274 ([$C_{18}H_{12}NS$]$^+$, 13), 272 (10), 191 (11), 152 (10). IR: $\tilde{\nu}$ [cm^{-1}] = 3010 (w), 2953 (w), 2930 (w), 2897 (w), 2833 (w), 1611 (w), 1574 (w), 1512 (m), 1485 (w), 1460 (s), 1437 (m), 1418 (w), 1389 (w), 1362 (w), 1302 (m), 1279 (m), 1242 (s), 1188 (w), 1169 (w), 1159 (w), 1128 (w), 1105 (w), 1076 (w), 1038 (m), 968 (w), 961 (w), 932 (w), 912 (w), 880 (w), 836 (m), 808 (w), 766 (s), 746 (s), 741 (m), 694 (m), 658 (w). Anal. calcd. for $C_{25}H_{19}NOS$ [381.5]: C 78.71, H 5.02, N 3.67, S 8.40; Found: C 78.51, H 5.06, N 3.53, S 8.69.

3,10-Diphenyl-10*H*-phenothiazine (4e)

The synthesis was performed by GP1. After chromatography on silica gel (*n*-hexane/acetone 50:1) and recrystallization from *n*-hexane/acetone, **4e** (118 mg, 0.338 mmol, 67%) was isolated as yellow crystals. Mp 196 °C. ^{1}H NMR (600 MHz, acetone-d$_6$): δ 6.86 (td, ^{3}J = 7.4 Hz, ^{4}J = 1.3 Hz, 1 H), 6.89-6.95 (m, 1 H), 7.07 (dd, ^{3}J = 7.5 Hz, ^{4}J = 1.6 Hz, 1 H), 7.20 (dd, ^{3}J = 8.5 Hz, ^{4}J = 2.2 Hz, 2 H), 7.28-7.32 (m, 1 H), 7.34 (d, ^{4}J = 2.2 Hz, 1 H), 7.40 (t, ^{3}J = 7.8 Hz, 2 H), 7.45- 7.50 (m, 2 H), 7.55-7.61 (m, 3 H), 7.72 (t, ^{3}J = 7.8 Hz, 2 H).^{13}C NMR (150 MHz, acetone-d$_6$): 117.0 (CH), 117.2 (CH), 120.5 (C$_{quat}$), 121.4 (C$_{quat}$), 123.6 (CH), 125.5 (CH), 126.3 (CH), 127.0 (CH), 127.5 (CH), 127.9 (CH), 128.0 (CH), 129.4 (CH), 129.7 (CH), 131.7 (CH), 131.9 (CH), 136.3 (C$_{quat}$), 140.4 (C$_{quat}$), 141.8 (C$_{quat}$), 144.4 (C$_{quat}$), 144.9 (C$_{quat}$). EI MS (70 eV): *m*/*z* (%): 352 (27), 351 ([M]$^+$, 100), 350 (20), 319 (18), 274 ([$C_{18}H_{12}NS$]$^+$, 22), 273 (13). IR: $\tilde{\nu}$ [cm^{-1}] = 3061 (w), 3030 (w), 3005 (w), 2951 (w), 1589 (w), 1574 (w), 1506 (w), 1489 (m), 1464 (s), 1439 (m), 1418 (w), 1389 (w), 1362 (w), 1306 (s), 1279 (m), 1256 (m), 1242 (m), 1223 (w), 1190 (w), 1157 (w), 1130 (w), 1070 (w), 1043 (w), 1022 (w), 966 (w), 918 (w), 901 (w), 887 (w), 843 (m), 822 (m), 808 (w), 768 (s), 750 (s), 723 (m), 689 (m), 673 (m), 660 (w), 635 (m). Anal. calcd. for $C_{24}H_{17}NS$ [351.5]: C 82.02, H 4.88, N 3.99, S 9.12; Found: C 82.30, H 4.91, N 3.75, S 9.15.

4-(3-Phenyl-10*H*-phenothiazin-10-yl)benzonitrile (4f)

The synthesis was performed by GP1. After chromatography on silica gel (*n*-hexane/acetone 50:1) and recrystallization from *n*-hexane/acetone, **4f** (117 mg, 0.311 mmol, 62%) was isolated as colorless crystals.

Mp 119 °C. ^{1}H NMR (500 MHz, acetone-d$_6$): δ 7.24 (dtd, ^{3}J = 7.8 Hz, ^{4}J = 3.7 Hz, 1.3 Hz, 2 H), 7.27-7.31 (m, 3 H), 7.33-7.40 (m, 2 H), 7.44-7.49 (m, 3 H), 7.61 (dd, ^{3}J = 8.3 Hz, ^{4}J = 2.1 Hz, 1 H), 7.66-7.70 (m, 2 H), 7.70-7.73 (m, 3 H).^{13}C NMR (125 MHz, acetone-d$_6$): 106.6 (C$_{quat}$), 119.5 (C$_{quat}$), 120.5 (CH), 125.5 (CH), 125.7 (CH), 126.8 (CH), 127.1 (CH), 127.4 (CH), 127.7 (CH), 128.6 (CH), 128.6 (CH), 129.4(CH) , 129.9 (CH), 131.5 (C$_{quat}$), 132.3 (C$_{quat}$), 134.8 (CH), 139.7(C$_{quat}$) , 140.3 (C$_{quat}$), 141.7 (C$_{quat}$), 142.4 (C$_{quat}$), 149.4 (C$_{quat}$). EI MS (70 eV): *m*/*z* (%): 377 (28), 376 ([M]$^+$, 100), 375 (22), 344 (15), 274 ([$C_{18}H_{12}NS$]$^+$, 27), 273 (16), 188 (11), 179 (12), 149 (21), 97 (14), 83 (13), 71 (18), 69 (17), 57 (28), 56 (16), 55 (17). IR: $\tilde{\nu}$ [cm^{-1}] = 3395 (w), 3374 (w), 3061 (w), 3049 (w), 3030 (w), 3011 (w), 2990 (w), 2957 (w), 1607 (w), 1591 (m), 1574 (w), 1551 (w), 1504 (m), 1479 (s), 1464 (m), 1441 (w), 1385 (w), 1313 (m), 1279 (w), 1263 (m), 1240 (w), 1175 (m), 1128 (w), 1076 (w), 1032 (w), 897 (w), 824 (s), 804 (w), 775 (w), 756 (s), 739 (m), 698 (m), 689 (m), 658 (w). Anal. calcd. for $C_{25}H_{16}N_2S$ [376.5]: C 79.76, H 4.28, N 7.44, S 8.52; Found: C 80.01, H 4.22, N 7.33, S 8.44.

10-(4-Methoxyphenyl)-3-(4-(trifluoromethyl)phenyl)-10*H*-phenothiazine (4g)

The synthesis was performed by GP1. After chromatography on silica gel (*n*-hexane/acetone 20:1) and recrystallization from *n*-hexane/acetone, **4g** (138 mg, 0.307 mmol, 61%) was isolated as yellow crystals. Mp 153 °C. ^{1}H NMR (600 MHz, acetone-d$_6$): δ 3.92 (s, 3 H), 6.22 (dd, ^3J = 8.2 Hz, ^4J = 1.1 Hz, 1 H), 6.29 (d, ^3J = 8.6 Hz, 1 H), 6.80-6.88 (m, 1 H), 6.91 (td, ^3J = 7.8 Hz, ^4J = 1.6 Hz, 1 H), 7.03 (dd, ^3J = 7.5 Hz, ^4J = 1.6 Hz, 1 H), 7.22-7.28 (m, 3 H), 7.33-7.42 (m, 3 H), 7.73 (d, ^3J = 8.2 Hz, 1 H), 7.79 (d, ^3J = 8.1 Hz, 1 H).^{13}C NMR (150 MHz, acetone-d$_6$): 56.0 (CH$_3$), 116.8 (CH), 116.9 (CH), 117.0 (CH), 119.9 (C$_{quat}$), 121.2 (C$_{quat}$), 123.6 (CH), 125.5 (C$_{quat}$, q, ^1J = 271.2 Hz), 125.7 (CH), 126.5 (CH, q, ^3J = 3.8 Hz), 126.7 (CH), 127.4 (CH), 127.5 (CH), 128.0 (CH), 129.1 (C$_{quat}$, q, ^2J = 32.1 Hz,), 132.9 (CH), 133.7 (C$_{quat}$), 134.1 (C$_{quat}$), 144.2 (C$_{quat}$), 145.0 (C$_{quat}$), 145.6 (C$_{quat}$), 160.6 (C$_{quat}$). EI MS (70 eV): *m*/*z* (%): 450 (27), 449 ([M]$^+$, 100), 434 ([$C_{25}H_{15}F_3NOS$]$^+$, 16), 342 ([$C_{19}H_{11}F_3NS$]$^+$, 17), 305 (17). IR: $\tilde{\nu}$ [cm^{-1}] = 3069 (w). 3051 (w), 2837 (w), 1607 (w), 1574 (w), 1510 (w), 1495 (w), 1464 (s), 1439 (m), 1325 (m), 1315 (s), 1298 (m), 1283 (m), 1261 (m), 1242 (m), 1192 (w), 1165 (m), 1121 (s), 1101 (m), 1070 (m), 1040 (m), 1013 (m), 961 (w), 932 (w), 916 (w), 885 (w), 837 (m), 824 (m), 812 (w), 756 (s), 739 (m), 716 (w), 629 (m), 610 (w). Anal. calcd. for $C_{26}H_{18}F_3NOS$ [449.5]: C 69.48, H 4.04, N 3.12, S 7.13; Found: C 69.24, H 4.04, N 3.07, S 7.28.

10-Phenyl-3-(4-(trifluoromethyl)phenyl)-10*H*-phenothiazine (4h)

The synthesis was performed by GP1. After chromatography on silica gel (*n*-hexane/acetone 20:1) and recrystallization from *n*-hexane/acetone, **4h** (137 mg, 0.327 mmol, 65%) was isolated as yellow crystals. Mp 125 °C. ^{1}H NMR (600 MHz, acetone-d$_6$): δ 6.20 (dd, ^3J = 8.3 Hz, ^4J = 1.3 Hz, 1 H), 6.28 (d, ^3J = 8.6 Hz, 1 H), 6.86 (td, ^3J = 7.4 Hz, ^4J = 1.3 Hz, 1 H), 6.89-6.94 (m, 1 H), 7.06 (dd, ^3J = 7.5 Hz, ^4J = 1.6 Hz, 1 H), 7.27 (dd, ^3J = 8.6 Hz, ^4J = 2.2 Hz, 1 H), 7.41 (d, ^4J = 2.2 Hz, 1 H), 7.45-7.49 (m, 2 H), 7.58-7.62 (m, 1 H), 7.70-7.75 (m, 4H), 7.80 (d, ^3J = 8.2 Hz, 2H).^{13}C NMR (150 MHz, acetone-d$_6$): 117.0 (CH), 117.1 (CH), 120.2 (C$_{quat}$), 121.5 (C$_{quat}$), 123.7 (CH), 125.5 (C$_{quat}$, q, ^1J = 271.0 Hz,), 125.8 (CH), 126.5 (CH, q, ^3J = 3.9 Hz), 126.7 (CH), 127.5 (CH), 127.5 (CH), 128.1 (CH), 129.1 (C$_{quat}$, q, ^2J = 32.1 Hz,), 129.6 (CH) 131.7 (CH), 132.0 (CH), 134.3 (C$_{quat}$), 141.6 (C$_{quat}$), 144.2 (C$_{quat}$), 144.7 (C$_{quat}$), 145.3 (C$_{quat}$). EI MS (70 eV): *m*/*z* (%): 420 (24), 419 ([M]$^+$, 100), 387 (18), 342 ([$C_{19}H_{11}F_3NS$]$^+$, 23), 275 (34), 274 ([$C_{18}H_{12}NS$]$^+$, 12), 273 ([$C_{18}H_{11}NS$]$^+$, 13), 243 (12), 241 (13). IR: $\tilde{\nu}$ [cm^{-1}] = 3061 (w). 3040 (w), 2955 (w), 1614 (w), 1591 (w), 1574 (w), 1495 (m), 1468 (s), 1439 (m), 1418 (w), 1387 (w), 1325 (s), 1312 (s), 1287 (m), 1260 (s), 1244 (m), 1159 (s), 1107 (s), 1070 (s), 1040 (m), 1015 (m), 1005 (w), 885 (w), 847 (s), 812 (s), 771 (m), 748 (s), 696 (s), 640 (m), 629 (m). Anal. calcd. for $C_{25}H_{16}F_3NS$ [419.5]: C 71.59, H 3.84, N 3.34, S 7.64; Found: C 71.73, H 4.02, N 3.20, S7.52.

4-(3-(4-(Trifluoromethyl)phenyl)-10*H*-phenothiazin-10-yl)benzonitrile (4i)

The synthesis was performed by GP1. After chromatography on silica gel (*n*-hexane/acetone 20:1) and recrystallization from *n*-hexane/acetone, **4i** (105 mg, 0.236 mmol, 47%) was isolated as yellow crystals. Mp 183 °C. ^{1}H NMR (600 MHz, acetone-d$_6$): δ 7.13 (dd, ^{3}J = 8.1 Hz, ^{4}J = 1.2 Hz, 1 H), 7.20-7.24 (m, 2 H), 7.32 (td, ^{3}J = 7.8 Hz, ^{4}J = 1.5 Hz, 1 H), 7.34-7.39 (m, 2 H), 7.44 (dd, ^{3}J = 7.7 Hz, ^{4}J = 1.5 Hz, 1 H), 7.65 (dd, ^{3}J = 8.4, ^{4}J = 2.2 Hz, 1 H), 7.76-7.83 (m, 5 H), 7.91 (d, ^{3}J = 8.1 Hz, 2 H).^{13}C NMR (150 MHz, acetone-d$_6$): 107.4 (C$_{quat}$), 119.4 (C$_{quat}$), 121.9 (CH), 124.5 (CH), 124.8 (CH), 126.3 (C$_{quat}$. q, ^{1}J = 271.3 Hz), 126.6 (CH), 126.7 (CH, q, ^{3}J = 3.9 Hz), 127.3 (CH), 127.6 (CH), 128.3 (CH), 128.6 (CH), 129.2 (CH), 129.8 (C$_{quat}$, q, ^{2}J = 32.2 Hz), 130.0 (C$_{quat}$), 131.3 (C$_{quat}$), 135.0 (CH), 137.5 (C$_{quat}$), 142.4 (C$_{quat}$), 142.7 (C$_{quat}$), 144.1 (C$_{quat}$), 148.7 (C$_{quat}$). EI MS (70 eV): *m*/*z* (%): 445 (23), 444 ([M]$^+$, 100), 412 (15), 342 ([$C_{19}H_{11}F_3NS$]$^+$, 37), 273 ([$C_{18}H_{11}NS$]$^+$, 13), 272 (11), 102 ([C_7H_4N]$^+$, 36), 75 (13). IR: $\tilde{\nu}$ [cm^{-1}] = 3044 (w). 1609 (m), 1591 (m), 1580 (w), 1506 (m), 1481 (m), 1464 (m), 1445 (w), 1435 (w), 1418 (w), 1381 (w), 1319 (s), 1314 (s), 1278 (m), 1263 (m), 1238 (w), 1177 (m), 1161 (m), 1123 (s), 1107 (s), 1068 (m), 1030 (w), 1015 (m), 968 (w), 947 (w), 920 (w), 893 (w), 881 (w), 864 (w), 847 (m), 822 (s), 764 (m), 750 (m), 737 (m), 727 (m), 700 (w), 692 (m), 667 (m), 650 (w), 631 (m). Anal. calcd. for $C_{26}H_{15}F_3N_2S$ [444.5]: C 70.26, H 3.40, N 6.30, S 7.21; Found: C 70.23, H 3.46, N 6.19, S 7.43.

10-(4-Methoxyphenyl)-3-(thiophen-2-yl)-10*H*-phenothiazine (4j)

The synthesis was performed by GP1. After chromatography on silica gel (*n*-hexane/acetone 20:1) and recrystallization from *n*-hexane/acetone, **4j** (72 mg, 0.186 mmol, 37%) was isolated as yellow crystals. Mp 161 °C. ^{1}H NMR (600 MHz, acetone-d$_6$): δ 3.92 (s, 3 H), 6.18-6.26 (m, 2H), 6.84 (td, ^{3}J = 7.4 Hz, ^{4}J = 1.3 Hz, 1 H), 6.91 (ddd, ^{3}J = 8.5 Hz, 7.4 Hz, ^{4}J = 1.6 Hz, 1 H), 7.03 (dd, ^{3}J = 7.6 Hz, ^{4}J = 1.6 Hz, 1 H), 7.06 (dd, ^{3}J = 5.1 Hz, 3.6 Hz, 1 H), 7.17 (dd, ^{3}J = 8.6 Hz, ^{4}J = 2.2 Hz, 1 H), 7.22-7.26 (m, 2 H), 7.29-7.33 (m, 2 H), 7.34-7.39 (m, 3 H). ^{13}C NMR (150 MHz, acetone-d$_6$): 55.9 (CH_3), 116.7 (CH), 116.9 (CH), 117.0 (CH), 119.8 (C$_{quat}$), 121.1 (C$_{quat}$), 123.3 (CH), 123.4 (CH), 124.2 (CH), 125.1 (CH), 125.3 (CH), 127.4 (CH), 128.0 (CH), 129.0 (CH), 129.8 (C$_{quat}$), 132.9 (CH), 133.8 (C$_{quat}$), 143.7 (C$_{quat}$), 144.8 (C$_{quat}$), 145.1 (C$_{quat}$), 160.6 (C$_{quat}$). EI MS (70 eV): *m*/*z* (%): 389 (12), 388 (26), 387 ([M]$^+$, 100), 372 ([$C_{22}H_{14}NOS_2$]$^+$, 10), 355 (11), 280 ([$C_{16}H_{10}NS_2$]$^+$, 20). (14), 200 (16). IR: $\tilde{\nu}$ [cm^{-1}] = 3051 (w), 2963 (w), 2930 (w), 2893 (w), 1607 (w), 1506 (w), 1489 (w), 1464 (m), 1456 (w), 1429 (m), 1398 (w), 1346 (w), 1310 (m), 1288(w), 1269 (w), 1242 (m), 1217 (w), 1192 (w), 1179 (w), 1165 (w), 1128 (w), 1101 (w), 1080 (w), 1028 (m), 1009 (w), 988 (w), 914 (w), 883 (w), 849 (w), 826 (w), 808 (s), 800 (w), 772 (w), 754 (m), 739 (s), 698 (s), 648 (w), 621 (w). Anal. calcd. for $C_{23}H_{17}NOS_2$ [387.5]: C 71.29, H 4.42, N 3.61, S 16.55; Found: C 71.32, H 4.54, N 3.54, S 16.28.

3-(4-Methoxyphenyl)-10-(pyridin-4-yl)-10*H*-phenothiazine (4k)

The synthesis was performed by GP1. After chromatography on silica gel (*n*-hexane/acetone 20:1) and precipitation from *n*-hexane, **4k** (128 mg, 0.335 mmol, 67%) was isolated as brown crystals. Mp 162 °C. ^{1}H NMR (300 MHz, acetone-d$_6$): δ 3.85 (s, 3 H), 6.89 (dd, ^{3}J = 4.8 Hz, ^{4}J = 1.6 Hz, 2 H), 7.01-7.08 (m, 2 H), 7.35 (td, ^{3}J = 7.5 Hz, ^{4}J = 1.5 Hz, 1 H), 7.48 (td, ^{3}J = 7.7 Hz, ^{4}J = 1.5 Hz, 1 H), 7.56-7.63 (m, 3 H), 7.63-7.66 (m, 1 H), 7.66-7.71 (m, 2 H), 7.78 (d, ^{4}J = 2.1 Hz, 1 H), 8.23-8.26 (m, 2 H). ^{13}C NMR (150 MHz, acetone-d$_6$): 55.8 (CH_3), 110.0 (CH), 115.4 (CH), 126.6 (CH), 127.4 (CH), 127.7 (CH), 128.2 (CH), 128.4 (CH), 128.6 (CH), 129.0 (CH), 129.8 (CH), 132.6 (C_{quat}), 135.1 (C_{quat}), 135.7 (C_{quat}), 139.8 (C_{quat}), 140.4 (C_{quat}), 141.4 (C_{quat}), 151.4 (CH), 152.6 (C_{quat}), 160.9 (C_{quat}). EI MS (70 eV): *m/z* (%): 383 (27), 382 ([M]$^+$, 100), 367 ([$C_{23}H_{15}N_2OS$]$^+$,24), 304 ([$C_{19}H_{14}NOS$]$^+$, 30), 289 ([$C_{18}H_{11}NOS$]$^+$, 10), 261 ([$C_{17}H_{11}NS$]$^+$, 31), 260 (14), 191 (12), 51 (13). IR: $\tilde{\nu}$ [cm^{-1}] = 3975 (w), 3798 (w), 3082 (w), 3009 (w), 2957 (w), 2932 (w), 2901 (w), 2833 (w), 1601 (m), 1577 (m), 1545 (w), 1520 (w), 1485 (m), 1464 (s), 1437 (m), 1423 (m), 1327 (m), 1312 (w), 1283 (m), 1265 (m), 1250 (m), 1225 (s), 1175 (m), 1163 (w), 1150 (w), 1130 (w), 1109 (w), 1076 (w), 1065 (w), 1040 (m), 1018 (m), 1009 (w), 989 (m), 953 (w), 926 (w), 880 (w), 806 (s), 758 (s), 743 (s), 723 (w), 789 (m), 687 (m), 656 (s), 631 (s), 621 (m). Anal. calcd. for $C_{24}H_{18}N_2OS$ [382.5]: C 75.37, H 4.74, N 7.32, S 8.38; Found: C 75.19, H 4.72, N 7.21, S 8.16.

10-(4-Methoxyphenyl)-3-(naphthalen-1-yl)-10*H*-phenothiazine (4l)

The synthesis was performed by GP1. After chromatography on silica gel (*n*-hexane/acetone 20:1) and recrystallization from *n*-hexane/acetone, **4l** (164 mg, 0.380 mmol, 76%) was isolated as yellow crystals. Mp 152 °C. ^{1}H NMR (300 MHz, acetone-d$_6$): δ 3.93 (s, 3 H), 6.23-6.29 (m, 1 H), 6.35 (d, ^{3}J = 8.4 Hz, 1 H), 6.85 (td, ^{3}J = 7.4 Hz, ^{4}J = 1.4 Hz, 1 H), 6.93 (ddd, ^{3}J = 8.2 Hz, 7.4 Hz, ^{4}J = 1.8 Hz, 1 H), 6.99-7.06 (m, 2 H), 7.13 (d, ^{4}J = 2.0 Hz, 1H), 7.22-7.30 (m, 2 H), 7.36-7.56 (m, 6 H), 7.87-7.97 (m, 3 H). ^{13}C NMR (75 MHz, acetone-d$_6$): δ 55.9 (CH_3), 116.5 (CH), 116.7 (CH), 117.0 (CH), 120.2 (C_{quat}), 120.6 (C_{quat}), 123.4 (CH), 126.4 (CH), 126.4 (CH), 126.7 (CH), 127.0 (CH), 127.4 (CH), 127.5 (CH), 128.0 (CH), 128.5 (CH), 128.5 (CH), 129.2 (CH), 129.5 (CH), 132.4 (C_{quat}), 133.1 (CH), 133.9 (C_{quat}), 134.9 (C_{quat}), 135.7(C_{quat}), 139.7 (C_{quat}), 144.8 (C_{quat}), 145.4 (C_{quat}), 160.6 (C_{quat}). EI MS (70 eV): *m/z* (%): 432 (29), 431 ([M]$^+$, 100), 324 ([$C_{22}H_{14}NS$]$^+$, 11), 322 (16), 291 (15), 290 (19), 202 (11). IR: $\tilde{\nu}$ [cm^{-1}] = 3059 (w), 2968 (w), 2930 (w), 2891 (w), 2827 (w), 1607 (w), 1576 (w), 1506 (m), 1497 (m), 1468 (s), 1456 (m), 1439 (m), 1391 (m), 1362 (w), 1315 (m), 1288 (m), 1238 (s), 1177 (w), 1163 (w), 1126 (w), 1103 (w), 1078 (m), 1028 (m), 1007 (w), 893 (w), 849 (w), 829 (m), 820 (m), 800 (m), 779 (s), 742 (s), 723 (w), 679 (w), 621 (w). Anal. calcd. for $C_{29}H_{21}NOS$ [431.6]: C 80.71, H 4.91, N 3.25, S 7.43; Found: C 80.51, H 5.16, N 3.16, S 7.23.

3-(4-Methoxyphenyl)-10-(naphthalen-1-yl)-10*H*-phenothiazine (4m)

The synthesis was performed by GP1. After chromatography on silica gel (*n*-hexane/acetone 20:1) and recrystallization from *n*-hexane/acetone, **4m** (121 mg, 0.280 mmol, 56%) was isolated as colorless crystals.

Mp 166 °C. ^{1}H NMR (300 MHz, acetone-d$_6$): δ 3.80 (s, 3 H), 6.04-6.13 (m, 2 H), 6.74-6.87 (m, 2 H), 6.92-6.98 (m, 2 H), 7.02 (dd, ^3J = 8.6 Hz, ^4J = 2.2 Hz, 1 H), 7.06-7.11 (m, 1 H), 7.30 (d, ^4J = 2.2 Hz, 1 H), 7.45-7.50 (m, 2 H), 7.51-7.65 (m, 2 H), 7.73-7.85 (m, 2 H), 8.08-8.19 (m, 3 H). ^{13}C NMR (75 MHz, acetone-d$_6$): δ 55.6 (CH_3), 115.1 (CH), 116.6 (CH), 117.0 (CH), 120.2 (C_{quat}), 120.9 (C_{quat}), 123.5 (CH), 124.0 (CH), 125.0 (CH), 125.9 (CH), 127.4 (CH), 127.6 (CH), 127.8 (CH), 128.0 (CH), 128.3 (CH), 129.8 (CH), 130.1 (CH), 130.8 (CH), 132.1 (C_{quat}), 132.7 (C_{quat}), 136.2 (C_{quat}), 136.8 (C_{quat}), 137.8 (C_{quat}), 143.4 (C_{quat}), 144.6 (C_{quat}), 160.2 (C_{quat}). EI MS (70 eV): *m/z* (%): 432 (28), 431 ([M]$^+$, 100), 416 ([$C_{28}H_{18}NOS$]$^+$, 21), 304 ([$C_{19}H_{14}NOS$]$^+$, 19), 288 ([$C_{18}H_{11}NOS$]$^+$, 12), 261 (37), 260 (27), 215 (18), 127 ([$C_{10}H_7$]$^+$, 60), 126 (17). IR: $\tilde{\nu}$ [cm^{-1}] = 3049 (w), 3020 (w), 1574 (w), 1489 (m), 1456 (s), 1436 (m), 1383 (w), 1292 (m), 1248 (m), 1234 (m), 1202 (w), 1175 (m), 1150 (w), 1128 (w), 1098 (w), 1047 (m), 1024 (m), 1007 (w), 905 (w), 880 (w), 824 (m), 800 (m), 781 (s), 764 (m), 750 (s), 741 (m), 719 (w), 708 (w), 681 (w), 654 (w), 627 (w). Anal. calcd. for $C_{29}H_{21}NOS$ [431.6]: C 80.71, H 4.91, N 3.25, S 7.43; Found: C 80.73, H 4.94, N 3.14, S 7.17.

10-(4-Methoxyphenyl)-3-(*o*-tolyl)-10*H*-phenothiazine (4n)

The synthesis was performed by GP1. After chromatography on silica gel (*n*-hexane/acetone 20:1) and recrystallization from *n*-hexane/acetone, **4n** (107 mg, 0.270 mmol, 54%) was isolated as colorless crystals.

Mp 194 °C. ^{1}H NMR (300 MHz, acetone-d$_6$): δ 2.24 (s, 3 H), 3.92 (s, 3 H), 6.16-6.32 (m, 3 H), 6.78-6.95 (m, 3 H), 6.98 (d, ^4J = 2.1 Hz, 1 H), 7.01-7.06 (m, 1 H), 7.10-7.33 (m, 6 H), 7.36-7.43 (m, 2 H). ^{13}C NMR (75 MHz, acetone-d$_6$): δ 20.6 (CH_3), 55.9 (CH_3), 116.3 (CH), 116.6 (CH), 117.0 (CH), 120.2 (C_{quat}), 120.3 (C_{quat}), 123.3 (CH), 126.7 (CH), 127.4 (CH), 127.7 (CH), 128.0 (CH), 128.1 (CH), 128.7 (CH), 130.3 (CH), 131.2 (CH), 133.1 (CH), 134.0 (C_{quat}), 135.9 (C_{quat}), 137.1 (C_{quat}), 141.4 (C_{quat}), 144.3 (C_{quat}), 145.5 (C_{quat}), 160.6 (C_{quat}). EI MS (70 eV): *m/z* (%): 396 (26), 395 ([M]$^+$, 100), 380 ([$C_{25}H_{18}NOS$]$^+$, 12), 305 (22), 273 ([$C_{18}H_{11}NS$]$^+$, 12), 254 (23), 197 ([$C_{12}H_7NS$]$^+$, 25). IR: $\tilde{\nu}$ [cm^{-1}] = 3061 (w), 3012 (w), 2955 (w), 2926 (w), 2835 (w), 1607 (w) 1574 (w), 1508 (m), 1464 (s), 1439 (m), 1418 (w), 1391 (w), 1306 (m), 1296 (m), 1238 (s), 1234 (m), 1190 (w), 1179 (w), 1163 (m), 1128 (w), 1119 (w), 1105 (m), 1078 (w), 1035 (m), 1010 (w), 970 (w), 914 (w), 891 (w), 843 (m), 822 (m), 797 (w), 752 (m), 721 (w), 704 (w), 656 (w). HR-MS (ESI) (m/z) calcd. for [$C_{26}H_{21}NO$]$^{\cdot+}$: 395.1344; Found: 395.1343. HPLC (acetone): 98% (RT = 7.2 min)

2-(10-(4-Methoxyphenyl)-10*H*-phenothiazin-3-yl)benzaldehyde (4o)

The synthesis was performed by GP1. After chromatography on silica gel (*n*-hexane/acetone 20:1) and recrystallization from *n*-hexane/acetone, **4o** (96 mg, 0.234 mmol, 47%) was isolated as yellow crystals. Mp 112 °C. ^{1}H NMR (600 MHz, acetone-d$_6$): δ 3.93 (s, 3 H), 6.24 (dd, ^3J = 8.3 Hz, ^4J = 1.2 Hz, 1 H), 6.32 (d, ^3J = 8.4 Hz, 1 H), 6.86 (td, ^3J = 7.5, ^4J = 1.3 Hz, 1 H), 6.91-6.97 (m, 2 H), 7.04 (dd, ^3J = 7.5, ^4J = 1.6 Hz, 1 H), 7.11 (d, ^4J = 2.2 Hz, 1 H), 7.21–7.28 (m, 2 H), 7.37-7.42 (m, 2 H), 7.46-7.49 (m, 1 H), 7.52 (tt, ^3J = 7.5 Hz, ^4J = 1.0 Hz, 1 H), 7.69 (td, ^3J = 7.6 Hz, ^4J = 1.5 Hz, 1H), 7.89-7.93 (m, 1 H), 10.00 (d, ^4J = 0.9 Hz, 1 H). ^{13}C NMR (150 MHz, acetone-d$_6$): δ 56.0 (CH$_3$), 116.3 (CH), 116.8 (CH), 117.0 (CH), 119.9 (C$_{quat}$), 120.9 (C$_{quat}$), 123.6 (CH), 127.4 (CH), 128.1 (CH), 128.2 (CH), 128.5 (CH), 128.5 (CH), 130.0 (CH), 131.5 (CH), 132.8 (C$_{quat}$), 133.0 (CH), 133.7 (C$_{quat}$), 134.5 (CH), 134.6 (C$_{quat}$), 145.2(C$_{quat}$), 145.3 (C$_{quat}$), 145.5 (C$_{quat}$), 160.6 (C$_{quat}$), 192.0 (C$_{quat}$). EI MS (70 eV): *m*/*z* (%): 410 (29), 409 ([M]$^+$, 100), 376 (22), 273 ([C$_{18}$H$_{11}$NS]$^+$, 12), 272 (11), 268 (15), 167 (11), 150 (14), 136 (12). IR: $\tilde{\nu}$ [cm^{-1}] = 3057 (w), 2953 (w), 1690 (m), 1605 (w), 1595 (w), 1574 (w), 1508 (s), 1460 (s), 1439 (m), 1387 (w), 1306 (m), 1244 (s), 1192 (m), 1183 (w), 1163 (w), 1128 (w), 1103 (w), 1080 (w), 1030 (m), 1011 (w), 829 (m), 762 (m), 745 (m), 640 (w). Anal. calcd. for $C_{26}H_{19}NO_2S$ [409.5]: C 76.26, H 4.68, N 3.42, S 7.83; Found: C 76.12, H 4.65, N 3.40, S 7.73.

3-(4-Methoxyphenyl)-10-(*o*-tolyl)-10*H*-phenothiazine (4p)

The synthesis was performed by GP1. After chromatography on silica gel (*n*-hexane/acetone 20:1) and recrystallization from *n*-hexane/acetone, **4o** (150 mg, 0.379 mmol, 76%) was isolated as yellow crystals. Mp 143 °C. ^{1}H NMR (600 MHz, acetone-d$_6$): δ 2.22 (s, 3 H), 3.81 (s, 3 H), 6.03 (d, ^3J = 8.2 Hz, 1 H), 6.06 (d, ^3J = 8.6 Hz, 1 H), 6.81 (dd, ^3J = 8.1 Hz, 6.7 Hz, 1 H), 6.87 (td, ^3J = 8.0 Hz, ^3J = 1.4 Hz, 1 H), 6.93-6.97 (m, 2 H), 6.99-7.01 (m, 1 H), 7.10 (dd, ^3J = 8.6 Hz, ^4J = 2.2 Hz, 1 H), 7.23 (d, ^4J = 2.2 Hz, 1 H), 7.37-7.42 (m, 1 H), 7.46-7.54 (m, 4 H), 7.55-7.59 (m, 1 H). ^{13}C NMR (150 MHz, acetone-d$_6$): δ 17.7 (CH$_3$), 55.6 (CH$_3$), 115.1 (CH), 115.8 (CH), 116.1 (CH), 119.5 (C$_{quat}$), 120.3 (C$_{quat}$), 123.3 (CH), 124.9 (CH), 125.9 (CH), 127.3, 128.0, 128.1, 129.3, 129.9, 132.4, 132.8, 133.4, 135.9, 139.0, 139.8, 142.3, 143.5, 160.1. EI MS (70 eV): *m*/*z* (%): 396 (27), 395 ([M]$^+$, 100), 380 ([C$_{25}$H$_{18}$NOS]$^+$, 26), 304 ([C$_{19}$H$_{14}$NOS]$^+$, 14), 289 ([C$_{18}$H$_{11}$NOS], 12), 261 (16), 197 ([C$_{12}$H$_7$NS]$^+$, 25). IR: $\tilde{\nu}$ [cm^{-1}] = 3055 (w), 3001 (w), 2947 (w), 2918 (w), 1603 (m), 1574 (m), 1491 (m), 1464 (s), 1437 (m), 1422 (w), 1383 (w), 1364 (w), 1306 (m), 1290 (m), 1254 (m), 1240 (s), 1204 (w), 1177 (m), 1161 (m), 1128 (w), 1113 (w), 1043 (m), 1024 (m), 989 (w), 955 (w), 916 (w), 889 (w), 816 (s), 802 (m), 779 (s), 748 (s), 733 (m), 658 (m), 632 (m). Anal. calcd. for $C_{26}H_{21}NOS$ [395.5]: C 78.96, H 5.35, N 3.54, S 8.11; Found: C 78.83, H 5.53, N 5.53, S 7.97.

General procedure 2 (GP2) for the synthesis of the 3,9-diaryl 9*H*-carbazoles 7

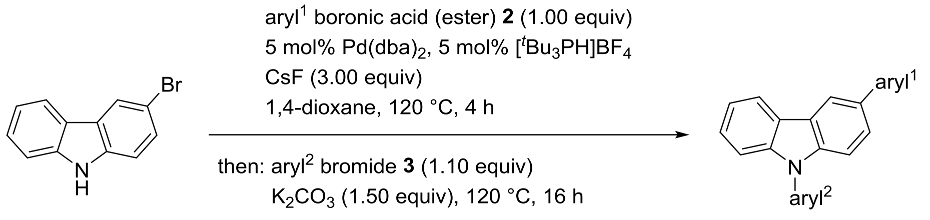

5 **7**

Under argon in a Schlenk tube with magnetic stir bar 3-bromo-9*H*-carbazole (**5**) (123 mg, 0.500 mmol), aryl boronic acid **2** (0.500 mmol), $Pd(dba)_2$ (14 mg, 5.0 mol%), tri-*tert*-butylphosphane tetrafluoroborate (8 mg, 5.0 mol%) and cesium fluoride (227 mg, 1.50 mmol) were dissolved in dry 1,4-dioxane (3 mL). The solution was degassed with argon for 5 min. Then, the reaction mixture was stirred at 120 °C (oil bath temperature) for 4 h. After cooling to room temperature, aryl bromide **3** (0.550 mmol) and potassium carbonate (207 mg, 1.50 mmol) were added and the reaction mixture was degassed with argon for 5 min. Then, the reaction mixture was stirred at 120 °C (oil bath temperature) for 20 h. After cooling to room temp deionized water (50 mL), saturated Na_2SO_3 solution (15 mL) and dichloromethane (50 mL) were successively added. The aqueous phase was extracted with dichloromethane (3 x 10 mL). The combined organic phases were dried with anhydrous magnesium sulfate and the solvents were removed in vacuo. The residue was purified by flash chromatography on silica gel and by recrystallization to give compound **7** as a solid.

Table S5: Experimental details for the synthesis of 3,9-diaryl 9*H*-carbazoles **7**.

entry	arylboronic acid **2**	aryl bromide **3**	yield of product **7**[a]
**1**	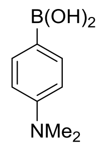 **2a**, 83 mg	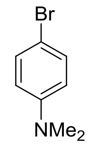 **3a**, 110 mg	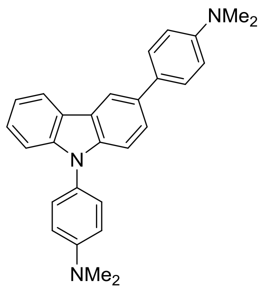 **7a**, 134 mg (66%)
**2**	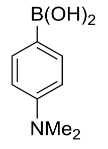 **2a**, 83 mg	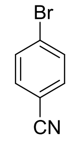 **3f**, 100 mg	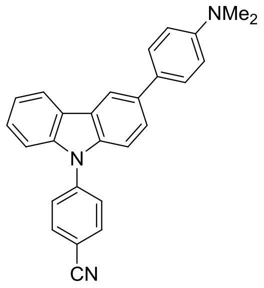 **7b**, 123 mg (63%)

[a] Yields after flash chromatography on silica gel.

Table S5: Experimental details for the synthesis of 3,9-diaryl 9*H*-carbazoles **7**.

entry	arylboronic acid **2**	aryl bromide **3**	yield of product **7**
**3**	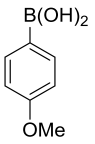<br>**2b**, 76 mg	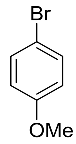<br>**3b**, 103 mg	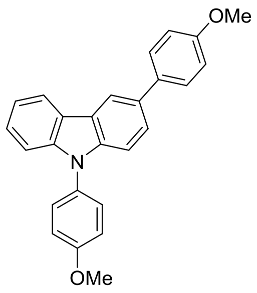<br>**7c**, 182 mg (96%)
**4**	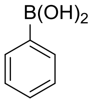<br>**2c**, 61 mg	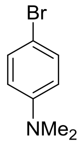<br>**3a**, 110 mg	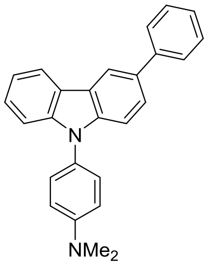<br>**7d**, 168 mg (93%)
**5**	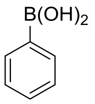<br>**2c**, 61 mg	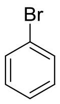<br>**3c**, 86 mg	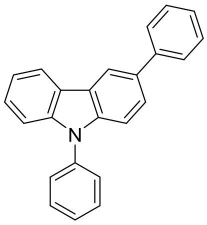<br>**7e**, 158 mg (99%)
**6**	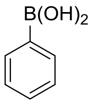<br>**2c**, 61 mg	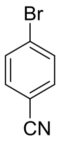<br>**3f**, 100 mg	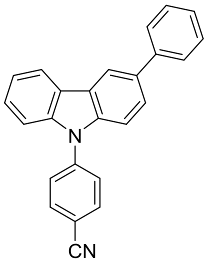<br>**7f**, 162 mg (94%)

[a] Yields after flash chromatography on silica gel.

Spectroscopic data of 3,9-diaryl carbazoles 7

4,4'-(9*H*-Carbazole-3,9-diyl)bis(*N*,*N*-dimethylaniline) (7a)

The synthesis was performed by GP2. After chromatography on silica gel (*n*-hexane/acetone 50:1) and recrystallization from *n*-hexane/acetone, **7a** (134 mg, 0.330 mmol, 66%) was isolated as colorless crystals.

Mp 165 °C. ^{1}H NMR (300 MHz, acetone-d$_6$): δ 2.98 (s, 6 H), 3.07 (s, 6 H), 6.82-6.91 (m, 2 H), 6.93-7.05 (m, 2 H), 7.20-7.34 (m, 3 H), 7.35-7.44 (m, 3 H), 7.58-7.68 (m, 3 H), 8.26 (ddd, ^3J = 7.8 Hz, ^4J = 1.3 Hz, 0.7 Hz, 1 H), 8.40 (dd, ^4J = 1.9 Hz, 0.7 Hz, 1 H). ^{13}C NMR (75 MHz, acetone-d$_6$): δ 40.7 (CH$_3$), 40.7 (CH$_3$), 110.6 (CH), 110.7 (CH), 113.8 (CH), 114.0 (CH), 118.2 (CH), 120.2 (CH), 121.2 (CH), 124.1 (C$_{quat}$), 124.4 (C$_{quat}$), 125.4 (CH), 126.7 (CH), 126.7 (C$_{quat}$), 128.3 (CH), 128.7 (CH), 130.8 (C$_{quat}$), 134.1 (C$_{quat}$), 141.3 (C$_{quat}$), 142.9 (C$_{quat}$), 150.6 (C$_{quat}$), 151.1 (C$_{quat}$). EI MS (70 eV): *m*/*z* (%): 406 (28), 405 ([M]$^+$, 100), 390 ([C$_{27}$H$_{24}$N$_3$]$^+$, 10), 389 (14), 203 (28), 202 (23), 194 (16), 180 (12). IR: $\tilde{\nu}$ [cm^{-1}] = 3042 (w), 3030 (w), 2913 (w), 2886 (w), 2849 (w), 1609 (m), 1522 (s), 1506 (m), 1476 (m), 1456 (m), 1441 (m), 1418 (w), 1348 (m), 1333 (m), 1315 (w), 1306 (w), 1275 (w), 1258 (m), 1233 (s), 1190 (m), 1165 (m), 1150 (w), 1128 (w), 1103 (w), 1063 (w), 1024 (w), 1009 (w), 995 (w), 939 (w), 907 (w), 887 (w), 827 (w), 808 (s), 766 (m), 754 (m), 745 (s), 725 (m), 704 (w), 648 (m). Anal. calcd. for C$_{28}$H$_{27}$N$_3$ [405.6]: C 82.93, H 6.71, N 10.36; Found: C 83.13, H 6.52, N 10.09.

4-(3-(4-(Dimethylamino)phenyl)-9*H*-carbazol-9-yl)benzonitrile (7b)

The synthesis was performed by GP2. After chromatography on silica gel (*n*-hexane/acetone 50:1) and recrystallization from *n*-hexane/acetone, **7b** (123 mg, 0.317 mmol, 63%) was isolated as colorless crystals.

Mp 170 °C. ^{1}H NMR (300 MHz, acetone-d$_6$): δ 2.98 (s, 6 H), 6.80-6.91 (m, 2 H), 7.33 (ddd, ^3J = 8.1 Hz, 7.1 Hz, ^4J = 1.1 Hz, 1 H), 7.46 (ddd, ^3J = 8.3 Hz, 7.0 Hz, ^4J = 1.3 Hz, 1 H), 7.50-7.57 (m, 2 H), 7.58-7.70 (m, 3 H), 7.85-7.95 (m, 2 H), 8.03-8.14 (m, 2 H), 8.30 (ddd, ^3J = 7.8 Hz, ^4J = 1.3 Hz, 0.7 Hz, 1 H), 8.42 (dd, ^3J = 1.9 Hz, 0.7 Hz, 1 H). ^{13}C NMR (75 MHz, acetone-d$_6$): δ 40.7 (CH$_3$), 110.6 (CH), 110.8 (CH), 111.2 (C$_{quat}$), 113.8 (CH), 118.4 (CH), 119.0 (C$_{quat}$), 121.5 (CH), 121.7 (CH), 125.1 (C$_{quat}$), 125.4 (C$_{quat}$), 125.8 (CH), 127.3 (CH), 128.0 (CH), 128.3 (CH), 130.1 (C$_{quat}$), 135.0 (CH), 135.5 (C$_{quat}$), 139.6 (C$_{quat}$), 141.2 (C$_{quat}$), 142.8 (C$_{quat}$), 150.8 (C$_{quat}$). EI MS (70 eV): *m*/*z* (%): 388 (25), 387 ([M]$^+$, 100), 372 ([C$_{26}$H$_{18}$N$_3$]$^+$, 11), 371 (12), 193 (34). IR: $\tilde{\nu}$ [cm^{-1}] = 2972 (w), 2886 (w), 2853 (w), 1599 (m), 1510 (m), 1476 (m), 1456 (s), 1445 (m), 1410 (w), 1354 (m), 1333 (m), 1315 (m), 1298 (w), 1275 (w), 1258 (w), 1229 (m), 1202 (w), 1184 (w), 1169 (m), 1155 (m), 1128 (w), 1109 (w), 1063 (m), 1028 (w), 1011 (w), 951 (w) 916 (w), 889 (w), 854 (w), 839 (m), 800 (s), 768 (m), 748 (s), 685 (w), 638 (m). Anal. calcd. for C$_{27}$H$_{21}$N$_3$ [387.5]: C 83.69, H 5.46, N 10.84; Found: C 83.88, H 5.40, N 10.64.

3,9-Bis(4-methoxyphenyl)-9*H*-carbazole (7c)

The synthesis was performed by GP2. After chromatography on silica gel (*n*-hexane/acetone 50:1), **7c** (182 mg, 0.479 mmol, 96%) was isolated as colorless crystals.
Mp 122 °C. ^{1}H NMR (600 MHz, acetone-d$_6$): δ 3.70 (s, 3 H), 3.79 (s, 3 H), 6.86-6.93 (m, 2 H), 7.06-7.10 (m, 2 H), 7.11-7.15 (m, 1 H), 7.19 (dd, ^3J = 14.9 Hz, 8.3 Hz, 2 H), 7.27 (ddd, ^3J = 8.2 Hz, 7.0 Hz, ^4J = 1.2 Hz, 1 H), 7.34-7.41 (m, 2 H), 7.51 (dd, ^3J = 8.5 Hz, ^4J = 1.8 Hz, 1 H), 7.53-7.56 (m, 2 H), 8.14 (d, ^3J = 7.8 Hz, 1 H), 8.30 (d, ^4J = 1.8 Hz, 1 H). ^{13}C NMR (150 MHz, acetone-d$_6$): δ 55.6 (CH_3), 56.0 (CH_3), 110.5 (CH), 110.7 (CH), 115.1 (CH), 116.1 (CH), 118.9 (CH), 120.6 (CH), 121.3 (CH), 124.1 (C_{quat}), 124.6 (C_{quat}), 125.8 (CH), 127.0 (CH), 128.8 (CH), 129.3 (CH), 130.9 (C_{quat}), 133.8 (C_{quat}), 135.1 (C_{quat}), 141.3 (C_{quat}), 142.6 (C_{quat}), 159.8 (C_{quat}), 160.1 (C_{quat}). EI MS (70 eV): *m*/*z* (%): 380 (27), 379 ([M]$^+$, 100), 365 (15), 364 ([$C_{25}H_{18}NO_2$]$^+$, 59), 293 (10), 292 (14), 190 (20), 168 (10), 146 (14). IR: $\tilde{\nu}$ [cm^{-1}] = 3011 (w), 2965 (w), 2949 (w), 2934 (w), 2903 (w), 2835 (w), 1601 (w), 1578 (w), 1510 (m), 1476 (m), 1456 (m), 1441 (m), 1398 (w), 1366 (w), 1335 (w), 1287 (m), 1227 (s), 1177 (m), 1155 (w), 1138 (w), 1103 (m), 1090 (w), 1030 (m), 1018 (m), 1001 (w), 951 (w), 934 (w), 910 (w), 876 (m), 851 (w), 831 (s), 814 (m), 795 (m), 766 (m), 746 (s), 723 (m), 714 (w), 700 (w), 660 (m), 629 (m). Anal. calcd. for $C_{26}H_{21}NO_2$ [379.5]: C 82.30, H 5.58, N 3.69; Found: C 82.06, H 5.49, N 3.52.

***N*,*N*-Dimethyl-4-(3-phenyl-9*H*-carbazol-9-yl)aniline (7d)**

The synthesis was performed by GP2. After chromatography on silica gel (*n*-hexane/acetone 50:1), **7d** (168 mg, 0.463 mmol, 93%) was isolated as colorless crystals.
Mp 87 °C. ^{1}H NMR (600 MHz, acetone-d$_6$): δ 3.07 (s, 6 H), 6.99 (d, ^3J = 8.7 Hz, 2 H), 7.25-7.29 (m, 1 H), 7.30-7.35 (m, 2 H), 7.36 (d, ^3J = 8.5 Hz, 1 H), 7.38-7.44 (m, 3 H), 7.47 (t, ^3J = 7.8 Hz, 2 H), 7.71 (dd, ^3J = 8.5 Hz, ^4J = 1.8 Hz, 1 H), 7.77 (dd, ^3J = 7.9 Hz, ^4J = 1.5 Hz, 2 H), 8.30 (d, ^3J = 7.8 Hz, 1H), 8.50 (d, ^3J = 1.8 Hz, 1 H). ^{13}C NMR (150 MHz, acetone-d$_6$): δ 40.6 (CH_3), 110.7 (CH), 110.9 (CH), 114.0 (CH), 119.4 (CH), 120.5 (CH), 121.3 (CH), 124.0 (C_{quat}), 124.4 (C_{quat}), 126.0 (CH), 126.5 (C_{quat}), 126.9 (CH), 127.3 (CH), 127.8 (CH), 128.7 (CH), 129.7 (CH), 133.6 (C_{quat}), 142.0 (C_{quat}), 142.8 (C_{quat}), 143.0 (C_{quat}), 151.1 (C_{quat}). EI MS (70 eV): *m*/*z* (%): 363 (29), 362 ([M]$^+$, 100), 346 ([$C_{25}H_{19}N_2$]$^+$, 10), 287 (12), 286 ([$C_{20}H_{17}N_2$]$^+$, 53), 243 (12), 242 (13), 241 ([$C_{18}H_{12}N$]$^+$, 20), 201 (11), 200 (10), 199 (10), 198 (16), 197 (55), 196 (53), 181 (50), 152 (15), 143 (12), 77 ([C_6H_5]$^+$, 12). IR: $\tilde{\nu}$ [cm^{-1}] = 3030 (w), 2936 (w), 2857 (w), 1610 (w), 1599 (w), 1520 (s), 1506 (m), 1472 (m), 1456 (s), 1348 (m), 1337 (m), 1325 (w), 1312 (w), 1296 (w), 1271 (w), 1256 (w), 1227 (s), 1233 (m), 1194 (m), 1165 (w), 1150 (w), 1130 (w), 1121 (w), 1063 (w), 1037 (w), 1028 (w), 1007 (w), 950 (w), 939 (w), 909 (w), 883 (w), 841 (w), 814 (m), 746 (m), 729 (m), 696 (m), 673 (w), 646 (w). Anal. calcd. for $C_{26}H_{22}N_2$ [362.5]: C 86.15, H 6.12, N 7.73; Found: C 85.91, H 6.19, N 7.53.

3,9-Diphenyl-9*H*-carbazole (7e)

The synthesis was performed by GP2. After chromatography on silica gel (*n*-hexane/acetone 50:1), **7e** (158 mg, 0.495 mmol, 99%) was isolated as colorless crystals.

Mp 139 °C[6]. ^{1}H NMR (600 MHz, acetone-d$_6$): δ 7.29-7.37 (m, 2 H), 7.40-7.52 (m, 5 H), 7.56 (tt, ^3J = 7.3 Hz, ^4J = 1.3 Hz, 1 H), 7.64-7.68 (m, 2 H), 7.70-7.76 (m, 3 H), 7.76-7.80 (m, 2 H), 8.33 (d, ^3J = 7.8 Hz, 1 H), 8.53 (d, ^4J = 1.8 Hz, 1 H). ^{13}C NMR (150 MHz, acetone-d$_6$): δ 110.6 (CH), 110.9 (CH), 119.5 (CH), 121.0 (CH), 121.4 (CH), 124.4 (C$_{quat}$), 124.9 (C$_{quat}$), 126.2 (CH), 127.2 (CH), 127.4 (CH), 127.8 (CH), 127.9 (CH), 128.6 (CH), 129.7 (CH), 131.0 (CH), 134.2 (C$_{quat}$), 138.4 (C$_{quat}$), 141.2 (C$_{quat}$), 142.2 (C$_{quat}$), 142.6 (C$_{quat}$). EI MS (70 eV): *m*/*z* (%): 320 (25), 319 ([M]$^+$, 100), 318 (12), 317 (11), 241 ([C$_{18}$H$_{12}$N]$^+$, 14). IR: $\tilde{\nu}$ [cm^{-1}] = 3528 (w), 3462 (w), 3269 (w), 3030 (w), 2955 (w), 2920 (w), 2872 (w), 2857 (w), 1624 (w), 1593 (w), 1497 (m), 1474 (m), 1452 (m), 1422 (w), 1362 (m), 1329 (w), 1271 (w), 1256 (w), 1236 (w), 1169 (w), 1150 (w), 1134 (w), 1072 (w), 1057 (w), 887 (w), 812 (m), 762 (s), 748 (m), 727 (m), 691 (m), 640 (m), 627 (w). Anal. calcd. for C$_{24}$H$_{17}$N [319.4]: C 90.25, H 5.36, N 4.39; Found: C 90.30, H 5.12, N 4.21.

4-(3-Phenyl-9*H*-carbazol-9-yl)benzonitrile (7f)

The synthesis was performed by GP2. After chromatography on silica gel (*n*-hexane/acetone 50:1), **7f** (162 mg, 0.470 mmol, 94%) was isolated as colorless crystals.

Mp 158 °C. ^{1}H NMR (600 MHz, acetone-d$_6$): δ 7.35 (t, ^3J = 7.5 Hz, 2 H), 7.45-7.52 (m, 3 H), 7.54 (d, ^3J = 8.2 Hz, 1 H), 7.58 (d, ^3J = 8.6 Hz, 1 H), 7.73-7.80 (m, 3 H), 7.92 (d, ^3J = 8.8 Hz, 2 H), 8.10 (d, ^3J = 8.7 Hz, 2 H), 8.33 (d, ^3J = 7.8 Hz, 1 H), 8.53 (d, ^4J = 1.8 Hz, 1 H). ^{13}C NMR (150 MHz, acetone-d$_6$): δ 110.7 (CH), 111.0 (CH), 111.4 (C$_{quat}$), 119.0 (C$_{quat}$), 119.6 (CH), 121.6 (CH), 121.9 (CH), 124.9 (C$_{quat}$), 125.4 (C$_{quat}$), 126.5 (CH), 127.5 (CH), 127.6 (CH), 127.9 (CH), 128.2 (CH), 129.7 (CH), 135.1 (CH), 140.3 (C$_{quat}$), 141.3 (C$_{quat}$), 142.3 (C$_{quat}$), 142.6 (C$_{quat}$). EI MS (70 eV): *m*/*z* (%): 345 (25), 344 ([M]$^+$, 100), 365 (15), 241 (11), 172 (11). IR: $\tilde{\nu}$ [cm^{-1}] = 3049 (w), 1599 (m), 1509 (s), 1474 (m), 1452 (s), 1425 (w), 1364 (m), 1341 (w), 1296 (w), 1273 (w), 1256 (w), 1234 (w), 1223 (m), 1190 (w), 1171 (m), 1153 (w), 1140 (w), 1117 (w), 1080 (w), 1067 (w), 1007 (w), 935 (w), 914 (w), 883 (m), 841 (m), 816 (m), 760 (s), 743 (s), 725 (s), 698 (s), 673 (m). Anal. calcd. for C$_{25}$H$_{16}$N$_2$ [344.4]: C 87.18, H 4.68, N 8.13; Found: C 87.27, H 4.77, N 7.93.

General procedure 3 (GP3) for the synthesis of the 1,5-diaryl 1*H*-indoles 8

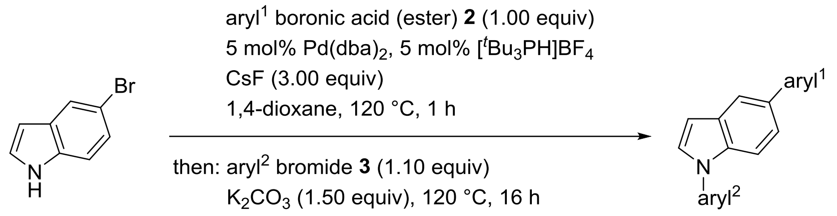

6 **8**

Under argon in a Schlenk tube with magnetic stir bar 5-bromo-1*H*-indole (**6**) (98.0 mg, 0.500 mmol), aryl boronic acid **2** (0.500 mmol), Pd(dba)$_2$ (14 mg, 5.0 mol%), tri-*tert*-butylphosphane tetrafluoroborate (8 mg, 5.0 mol%) and cesium fluoride (227 mg, 1.50 mmol) were dissolved in dry 1,4-dioxane (3 mL). The solution was degassed with argon for 5 min. Then, the reaction mixture was stirred at 120 °C (oil bath temperature) for 1 h. After cooling to room temperature, aryl bromide **3** (0.550 mmol) and potassium carbonate (207 mg, 1.50 mmol) were added and the reaction mixture was degassed with argon for 5 min. Then, the reaction mixture was stirred at 120 °C (oil bath temperature) for 20 h. After cooling to room temp deionized water (50 mL), saturated Na_2SO_3 solution (15 mL) and dichloromethane (50 mL) were successively added. The aqueous phase was extracted with dichloromethane (3 x 10 mL). The combined organic phases were dried with anhydrous magnesium sulfate and the solvents were removed in vacuo. The residue was purified by flash chromatography on silica gel and by recrystallization to give compound **7** as a solid.

Table S6: Experimental details for the synthesis of 1,5-diaryl 1*H*-indoles **8**.

entry	arylboronic acid or ester **2**	aryl bromide **3**	yield of product 8[a]
**1**	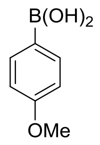 **2b**, 76 mg	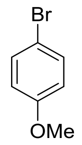 **3b**, 103 mg	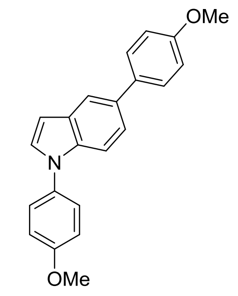 **8a**, 136 mg (83%)
**2**	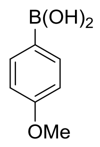 **2b**, 76 mg	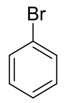 **3c**, 86 mg	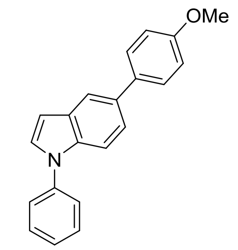 **8b**, 132 mg (88%)

[a] Yields after flash chromatography on silica gel.

Table S6: Experimental details for the synthesis of 1,5-diaryl 1*H*-indoles **8**

entry	arylboronic acid **2**	aryl bromide **3**	yield of product **8**
**3**	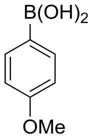 **2b**, 76 mg	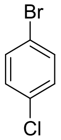 **3d**, 105 mg	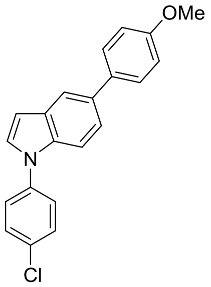 **8c**, 48 mg (29%)
**4**	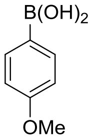 **2b**, 76 mg	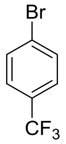 **3e**, 124 mg	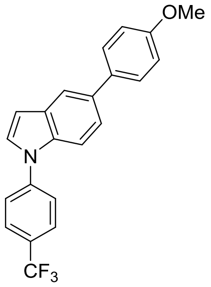 **8d**, 139 mg (76%)
**5**	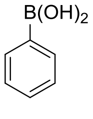 **2c**, 61 mg	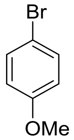 **3b**, 103 mg	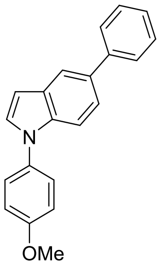 **8e**, 112 mg (75%)
**6**	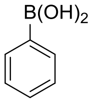 **2c**, 61 mg	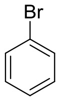 **3c**, 86 mg	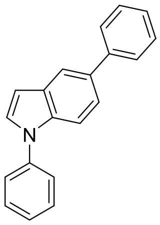 **8f**, 106 mg (79%)
**7**	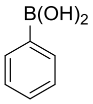 **2c**, 61 mg	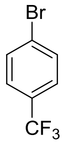 **3e**, 124 mg	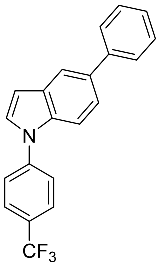 **8g**, 135 mg (80%)

[a] Yields after flash chromatography on silica gel.

Table S6: Experimental details for the synthesis of 1,5-diaryl 1*H*-indoles **8**.

entry	arylboronic acid or ester **2**	aryl bromide **3**	yield of product **8**
**8**	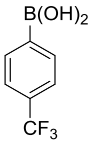 **2d**, 95 mg	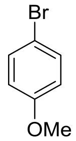 **3b**, 103 mg	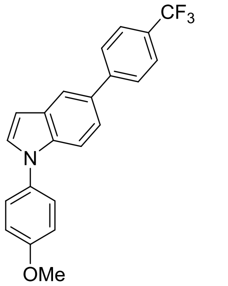 **8h**, 134 mg (73%)
**9**	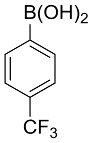 **2d**, 95 mg	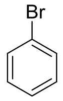 **3c**, 86 mg	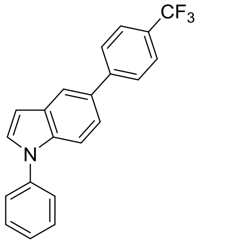 **8i**, 136 mg (81%)
**10**	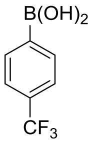 **2d**, 95 mg	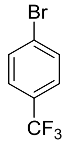 **3e**, 124 mg	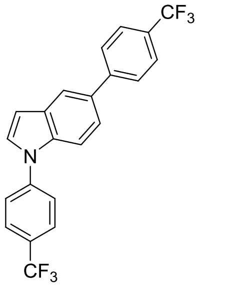 **8j**, 142 mg (70%)
**11**	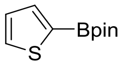 **2e**, 105 mg	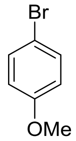 **3b**, 103 mg	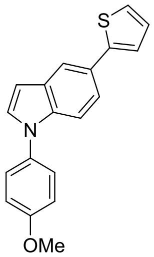 **8k**, 90 mg (59%)
**12**	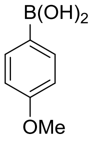 **2b**, 76 mg	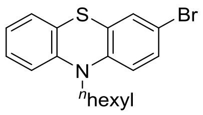 **3i**, 199mg	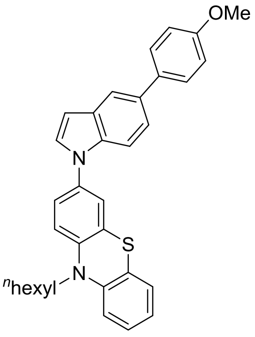 **8l**, 114 mg (45%)

[a] Yields after flash chromatography on silica gel.

Table S6: Experimental details for the synthesis of 1,5-diaryl 1*H*-indoles **8**.

entry	arylboronic acid or ester **2**	aryl bromide **3**	yield of product **8**[a]
**13**	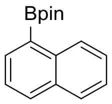 **2f**, 127 mg	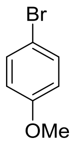 **3b**, 103 mg	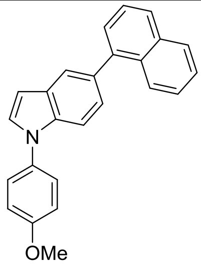 **8m**, 50 mg (29%)
**14**	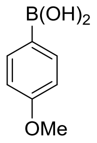 **2b**, 76 mg	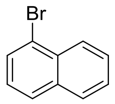 **3g**, 114 mg	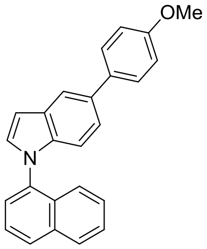 **8n**, 102 mg (58%)

[a] Yields after flash chromatography on silica gel.

Spectroscopic data of 1,5-diaryl indoles 8

1,5-Bis(4-methoxyphenyl)-1*H*-indole (8a)

The synthesis was performed by GP3. After chromatography on silica gel (*n*-hexane/acetone 20:1) and recrystallization from *n*-hexane, **8a** (136 mg, 0.413 mmol, 83%) was isolated as colorless crystals.
Mp 115 °C. ^{1}H NMR (600 MHz, acetone-d$_6$): δ 3.84 (s, 3 H), 3.89 (s, 3 H), 6.69 (dd, ^{3}J = 3.2 Hz, ^{4}J = 0.8 Hz, 1 H), 7.00-7.03 (m, 2 H), 7.12-7.17 (m, 2 H), 7.44 (dd, ^{3}J = 8.6 Hz, ^{4}J = 1.8 Hz, 1 H), 7.46 (d, ^{3}J = 3.2 Hz, 1 H), 7.50 (d, ^{3}J = 8.6 Hz, 1 H), 7.49-7.52 (m, 2 H) 7.60-7.62 (m, 2 H), 7.85 (dd, ^{4}J = 1.8 Hz, 0.7 Hz, 1 H). ^{13}C NMR (150 MHz, acetone-d$_6$): 55.6 (CH$_3$), 55.9 (CH$_3$), 104.1 (CH), 111.3 (CH), 115.0 (CH), 115.7 (CH), 119.5 (CH), 122.3 (CH), 126.5 (CH), 128.8 (CH), 129.8 (CH), 130.8 (C$_{quat}$), 133.5 (C$_{quat}$), 134.0 (C$_{quat}$), 135.5 (C$_{quat}$), 136.3 (C$_{quat}$), 159.3 (C$_{quat}$), 159.7 (C$_{quat}$). EI MS (70 eV): *m*/*z* (%): 330 (24), 329 ([M]$^{+}$, 100), 315 (13), 314 ([$C_{21}H_{16}NO_2$]$^{+}$, 52), 289 (11), 165 (15), 143 (10), 122 (12). IR: $\tilde{\nu}$ [cm^{-1}] = 3007 (w), 2963 (w), 2938 (w), 2907 (w), 2835 (w), 1607 (w), 1514 (s), 1504 (m), 1439 (m), 1339 (w), 1290 (w), 1273 (m), 1246 (s), 1234 (s), 1171 (m), 1155 (m), 1119 (m), 1107 (m), 1069 (w), 1030 (s), 1016 (m), 959 (w), 945 (w), 901 (m), 880 (w), 829 (s), 802 (s), 797 (m), 781 (m), 764 (s), 733 (m), 721 (m), 644 (m), 621 (m), 602 (s). Anal. calcd. for $C_{22}H_{19}NO_2$ [329.4]: C 80.22, H 5.81, N 4.25; Found: C 79.98, H 5.55, N 4.37.

5-(4-Methoxyphenyl)-1-phenyl-1*H*-indole (8b)

The synthesis was performed by GP3. After chromatography on silica gel (*n*-hexane/acetone 20:1) and recrystallization from *n*-hexane, **8b** (132 mg, 0.441 mmol, 88%) was isolated as colorless crystals.
Mp 130 °C. ^{1}H NMR (600 MHz, acetone-d$_6$): δ 3.84 (s, 3 H), 6.74 (d, ^{3}J = 3.1 Hz, 1 H), 7.02 (d, ^{3}J = 8.5 Hz, 2 H), 7.42 (t, ^{3}J = 6.7 Hz, 1 H), 7.47 (td, ^{3}J = 8.6 Hz, 1 H), 7.56 (d, ^{3}J = 3.2 Hz, 1 H), 7.58-7.65 (m, 7 H), 7.87 (d, ^{4}J = 1.7 Hz, 1 H). ^{13}C NMR (150 MHz, acetone-d$_6$): 55.6 (CH$_3$), 104.8 (CH), 111.5 (CH), 115.0 (CH), 119.6 (CH), 122.5 (CH), 124.8 (CH), 127.3 (CH), 128.8 (CH), 129.49 (CH), 130.2 (CH), 131.2 (C$_{quat}$), 134.3 (C$_{quat}$), 135.4 (C$_{quat}$), 135.8 (C$_{quat}$), 140.7 (C$_{quat}$), 159.7 (C$_{quat}$). EI MS (70 eV): *m*/*z* (%): 300 (23), 299 ([M]$^{+}$, 100), 285 (15), 284 ([$C_{20}H_{14}NO$]$^{+}$, 67), 256 ([$C_{19}H_{14}N$]$^{+}$, 25), 254 (10), 152 (11), 150 (18). IR: $\tilde{\nu}$ [cm^{-1}] = 3034 (w), 3011 (w), 2995 (w), 2957 (w), 2932 (w), 2901 (w), 2833 (w), 1595 (m), 1582 (w), 1530 (w), 1514 (w), 1497 (s), 1462 (m), 1445 (m), 1414 (w), 1369 (w), 1335 (w), 1315 (w), 1300 (w), 1271 (m), 1246 (m), 1229 (m), 1177 (m), 1165 (w), 1115 (w), 1080 (w), 1042 (m), 1020 (w), 951 (w), 881 (w), 837 (s), 791 (s), 758 (s), 718 (m), 702 (s), 663 (m). Anal. calcd. for $C_{21}H_{17}NO$ [299.4]: C 84.25, H 5.72, N 4.68; Found: C 84.37, H 5.80, N 4.66.

1-(4-Chlorophenyl)-5-(4-methoxyphenyl)-1*H*-indole (8c)

The synthesis was performed by GP3. After chromatography on silica gel (*n*-hexane/acetone 20:1) and recrystallization from *n*-hexane, **8c** (48 mg, 0.144 mmol, 29%) was isolated as yellow crystals.

Mp 128 °C. ^{1}H NMR (300 MHz, acetone-d$_6$): δ 3.84 (s, 3 H), 6.76 (dd, ^{3}J = 3.3 Hz, ^{4}J = 0.8 Hz, 1 H), 6.99-7.05 (m, 2 H), 7.45-7.51 (m, 1 H), 7.55-7.58 (m, 1 H), 7.58-7.62 (m, 2 H), 7.62-7.71 (m, 5 H), 7.87 (dd, ^{4}J = 1.8 Hz, 0.6 Hz, 1 H). ^{13}C NMR (75 MHz, acetone-d$_6$): 55.6 (CH$_3$), 105.3 (CH), 111.4 (CH), 115.1 (CH), 115.2 (CH), 119.7 (CH), 122.7 (CH), 126.3 (CH), 128.3 (CH), 128.8 (CH), 129.4 (CH), 129.6 (CH), 130.7 (CH), 131.3 (C$_{quat}$), 132.1 (C$_{quat}$), 134.5 (C$_{quat}$), 135.3 (C$_{quat}$), 135.7 (C$_{quat}$), 139.5 (C$_{quat}$), 159.8 (C$_{quat}$). EI MS (70 eV): *m*/*z* (%): 335 ([$C_{21}H_{16}{}^{37}ClNO]^+$, 34), 334 (25), 333 ([$C_{21}H_{16}{}^{35}ClNO]^+$, 100), 320 (18), 319 (12), 318 ([$C_{20}H_{13}{}^{35}ClNO$], 58), 290 ([$C_{19}H_{13}{}^{35}ClN]^+$, 22), 254 (15), 167 (10), 152 (12), 127 (13). IR: $\tilde{\nu}$ [cm^{-1}] = 2953 (w), 2926 (w), 2901 (w), 1605 (w), 1578 (w), 1522 (m), 1493 (m), 1452 (s), 1437 (m), 1369 (m), 1337 (m), 1300 (m), 1275 (m), 1242 (s), 1231 (m), 1182 (m), 1136 (m), 1119 (m), 1101 (m), 1090 (m), 1070 (m), 1040 (m), 1015 (m), 955 (m), 889 (m), 874 (w), 835 (m), 804 (s), 764 (m), 746 (m), 718 (m), 613 (m). Anal. calcd. for $C_{21}H_{16}ClNO$ [333.8]: C 75.56, H 4.83, N 4.20; Found C 75.46, H 4.94, N 3.95.

5-(4-Methoxyphenyl)-1-(4-(trifluoromethyl)phenyl)-1*H*-indole (8d)

The synthesis was performed by GP3. After chromatography on silica gel (*n*-hexane/acetone 20:1) and recrystallization from *n*-hexane, **8d** (139 mg, 0.378 mmol, 76%) was isolated as colorless crystals.

Mp 152 °C. ^{1}H NMR (300 MHz, acetone-d$_6$): δ 3.84 (s, 3 H), 6.79-6.83 (m, 1 H), 6.99-7.06 (m, 2 H), 7.51 (dd, ^{3}J = 8.1 Hz, ^{4}J = 1.7 Hz, 1 H), 7.60-7.65 (m, 2 H), 7.62-7.71 (m, 1 H), 7.71-7.76 (m, 1 H), 7.85-7.91 (m, 3 H), 7.95 (d, ^{3}J = 8.9 Hz , 2 H). ^{13}C NMR (75 MHz, acetone-d$_6$): 55.7 (CH$_3$), 106.2 (CH), 111.8 (CH), 115.2 (CH), 119.9 (CH), 123.1 (CH), 124.9 (CH), 125.4 (C$_{quat}$, q, ^{1}J = 271.1 Hz), 128.2 (C$_{quat}$, q, ^{2}J = 32.6 Hz), 128.0 (CH, q, ^{3}J = 3.8 Hz), 129.0 (CH), 129.3 (CH), 131.8 (C$_{quat}$), 135.03 (C$_{quat}$), 135.3 (C$_{quat}$), 135.6 (C$_{quat}$), 144.1 (C$_{quat}$), 160.0 (C$_{quat}$). EI MS (70 eV): *m*/*z* (%): 368 (24), 367 ([M]$^+$, 100), 353 (16), 352 ([$C_{21}H_{13}F_3NO]^+$, 66), 324 ([$C_{20}H_{13}F_3N]^+$, 32), 184 (19), 152 (17), 151 (10). IR: $\tilde{\nu}$ [cm^{-1}] = 3109 (w), 3065 (w), 2953 (w), 2901 (w), 2838 (w), 1609 (w), 1526 (m), 1506 (w), 1464 (m), 1443 (w), 1377 (w), 1312 (m), 1300 (m), 1288 (w), 1269 (m), 1240 (m), 1225 (m), 1163 (m), 1107 (s), 1063 (s), 1042 (m), 1009 (m), 955 (w), 883 (m), 849 (m), 831 (s), 804 (s), 764 (m), 719 (s), 617 (s). Anal. calcd. for $C_{22}H_{16}F_3NO$ [367.4]: C 71.93, H 4.39, N 3.81; Found: C 72.05, H 4.44, N 3.72.

1-(4-Methoxyphenyl)-5-phenyl-1*H*-indole (8e)

The synthesis was performed by GP3. After chromatography on silica gel (*n*-hexane/acetone 20:1) and recrystallization from *n*-hexane, **8e** (112 mg, 0.375 mmol,75%) was isolated as yellow crystals.
Mp 110 °C. ^{1}H NMR (300 MHz, acetone-d$_6$): δ 3.90 (s, 3 H), 6.72 (dd, ^3J = 3.2 Hz, ^4J = 0.7 Hz, 1 H), 7.12-7.18 (m, 2 H), 7.28-7.34 (m, 1 H), 7.41-7.56 (m, 7 H), 7.67-7.71 (m, 2 H), 7.92 (dd, ^4J = 1.7 Hz, 0.8 Hz, 1 H). ^{13}C NMR (75 MHz, acetone-d$_6$): 55.9 (CH_3), 104.2 (CH), 111.4 (CH), 115.7 (CH), 116.9 (CH), 120.1 (CH), 122.5 (CH), 126.5 (CH), 127.2 (CH), 127.9 (CH), 129.6 (CH), 130.0 (CH), 130.8 (C_{quat}), 133.02 (CH), 133.5 (C_{quat}), 134.2 (C_{quat}), 136.7 (C_{quat}), 143.1 (C_{quat}), 159.4 (C_{quat}). EI MS (70 eV): *m*/*z* (%): 300 (24), 299 ([M]$^+$, 100), 285 (11), 284 ([$C_{20}H_{14}NO$]$^+$, 50), 256 ([$C_{19}H_{14}N$]$^+$, 14), 254 (12), 150 (14), 127 (16), 125 (11), 111 (16), 97 (22), 95 (15), 85 (15), 83 (17), 81 (14), 71 (19), 69 (18), 57 (26), 55 (20). IR: $\tilde{\nu}$ [cm^{-1}] = 2959 (w), 2928 (w), 2901 (w), 2832 (w), 1597 (w), 1510 (m), 1452 (s), 1437 (m), 1373 (w), 1362 (w), 1337 (m), 1298 (m), 1244 (s), 1229 (m), 1198 (m), 1177 (m), 1169 (m), 1152 (m), 1117 (m), 1105 (m), 1078 (m), 1034 (m), 1024 (m), 1011 (m), 989 (w), 957 (m), 893 (m), 881 (m), 858 (w), 831 (s), 816 (m), 785 (m), 754 (s), 737 (m), 727 (m), 716 (m), 696 (s), 652 (m), 637 (m). Anal. calcd. for $C_{21}H_{17}NO$ [299.4]: C 84.25, H 5.72, N 4.68; Found C 84.39, H 5.94, N 4.69.

1,5-Diphenyl-1*H*-indole (8f)

The synthesis was performed by GP3. After chromatography on silica gel (*n*-hexane/acetone 20:1) and recrystallization from *n*-hexane, **8f** (106 mg, 0.394 mmol, 79%) was isolated as colorless crystals.
Mp 104 °C.[7] ^{1}H NMR (300 MHz, acetone-d$_6$): δ 6.77 (dd, ^3J = 3.3 Hz, ^4J = 0.8 Hz, 1 H), 7.28-7.35 (m, 1 H), 7.39-7.49 (m, 3 H), 7.52 (dd, ^3J = 8.6 Hz, ^4J = 1.8 Hz, 1 H), 7.57-7.59 (m, 1 H), 7.59-7.63 (m, 2 H), 7.63-7.68 (m, 3 H), 7.68-7.70 (m, 1 H),7.70-7.73 (m, 1 H), 7.94 (dd, ^4J = 1.8 Hz, 0.7 Hz, 1 H). ^{13}C NMR (75 MHz, acetone-d$_6$): 104.9 (CH), 111.6 (CH), 120.2 (CH), 122.7 (CH), 124.9 (CH), 127.3 (CH), 127.4 (CH), 127.9 (CH), 129.7 (CH), 130.7 (CH), 131.2 (C_{quat}), 134.5 (C_{quat}), 136.2 (C_{quat}), 140.6 (C_{quat}), 143.0 (C_{quat}). EI MS (70 eV): *m*/*z* (%): 270 (20), 269 ([M]$^+$, 100), 268 (14), 165 ([$C_{13}H_9$]$^+$, 20). IR: $\tilde{\nu}$ [cm^{-1}] = 3753 (w), 3736 (w), 3258 (w), 3238 (w), 3132 (w), 3111 (w), 3053 (w), 3028 (w), 3015 (w), 2953 (w), 1595 (m), 1558 (w), 1491 (m), 1466 (m), 1456 (m), 1433 (m), 1369 (m), 1350 (m), 1339 (m), 1327 (w), 1312 (w), 1283 (w), 1265 (w), 1229 (m), 1198 (w), 1175 (m), 1150 (w), 1117 (m), 1072 (m), 1018 (w), 955 (m), 905 (w), 889 (m), 856 (w), 812 (m), 787 (m), 752 (s), 727 (s), 694 (s), 681 (m), 662 (w), 638 (w), 627 (m). Anal. calcd. for $C_{20}H_{15}N$ [269.4]: C 89.19, H 5.61, N 5.20; Found: C 89.15, H 5.84, N 5.01.

5-Phenyl-1-(4-(trifluoromethyl)phenyl)-1*H*-indole (8g)

The synthesis was performed by GP3. After chromatography on silica gel (*n*-hexane/acetone 20:1) and recrystallization from *n*-hexane, **8g** (135 mg, 0.400 mmol, 80%) was isolated as colorless crystals. Mp 120 °C. ^{1}H NMR (600 MHz, acetone-d$_6$): δ 6.84 (d, ^3J = 3.3 Hz, 1 H), 7.33 (t, ^3J = 7.4 Hz, 1 H), 7.46 (t, ^3J = 7.7 Hz, 2 H), 7.57 (dd, ^3J = 8.6 Hz, ^4J = 1.8 Hz, 1 H), 7.69-7.71 (m, 2 H), 7.71-7.72 (m, 1 H), 7.77 (d, ^3J = 8.6 Hz, 1 H), 7.90 (d, ^3J = 8.6 Hz, 2 H), 7.96 (dd, ^3J = 5.1 Hz, 3.3 Hz, 3 H). ^{13}C NMR (150 MHz, acetone-d$_6$): 106.2 (CH), 111.8 (CH), 120.5 (CH), 123.3 (CH), 124.9 (CH), 125.3 (C$_{quat}$, q, ^1J = 271.1 Hz), 127.5 (CH), 127.9 (CH, q, ^3J = 3.4 Hz), 128.4 (C$_{quat}$, q, ^2J = 32.7 Hz), 129.4 (CH), 129.7 (CH), 131.7 (C$_{quat}$), 135.3 (C$_{quat}$), 136.0 (C$_{quat}$), 142.9 (C$_{quat}$), 144.0 (C$_{quat}$). EI MS (70 eV): *m*/*z* (%): 338 (23), 337 ([M]$^+$, 100), 165 ([$C_{13}H_9$]$^+$, 24). IR: $\tilde{\nu}$ [cm^{-1}] = 3065 (w), 3030 (w), 1609 (m), 1599 (w), 1518 (m), 1466 (m), 1452 (m), 1423 (w), 1375 (m), 1317 (s), 1298 (m), 1288 (m), 1267 (m), 1227 (m), 1157 (m), 1119 (s), 1107 (s), 1063 (s), 1036 (m), 1015 (m), 999 (w), 957 (m), 939 (w), 914 (w), 907 (w), 870 (m), 847 (m), 839 (m), 810 (m), 785 (m), 754 (s), 712 (s), 700 (s), 691 (m), 667 (w), 633 (m), 602 (m). Anal. calcd. for $C_{21}H_{14}F_3N$ [337.4]: C 74.77, H 4.18, N 4.15; Found C 75.01, H 4.30, N 4.10.

1-(4-Methoxyphenyl)-5-(4-(trifluoromethyl)phenyl)-1*H*-indole (8h)

The synthesis was performed by GP3. After chromatography on silica gel (*n*-hexane/acetone 20:1) and recrystallization from *n*-hexane, **8h** (134 mg, 0.365 mmol, 73%) was isolated as colorless crystals. Mp 111 °C. ^{1}H NMR (600 MHz, acetone-d$_6$): δ 3.90 (s, 3 H), 6.76 (d, ^3J = 3.2 Hz, 1 H), 7.14-7.18 (m, 2 H), 7.52 (dd, ^3J = 6.0 Hz, ^4J = 2.8 Hz, 3 H), 7.56 (s, 2 H), 7.79 (d, ^3J = 8.2 Hz, 2 H), 7.93 (d, ^3J = 8.1 Hz, 2 H), 8.02 (s, 1 H). ^{13}C NMR (150 MHz, acetone-d$_6$): 56.0 (CH_3), 104.3 (CH), 111.7 (CH), 115.8 (CH), 120.6 (CH), 122.5 (CH), 125.67 (C$_{quat}$, q, ^1J = 270.9 Hz), 126.5 (CH,q, ^3J = 3.8 Hz), 126.6 (CH), 128.4 (CH), 128.6 (C$_{quat}$, q, ^2J = 32.0 Hz), 130.4 (CH), 130.8 (C$_{quat}$), 132.4 (C$_{quat}$), 133.3 (C$_{quat}$), 137.2 (C$_{quat}$), 147.0 (C$_{quat}$), 159.5 (C$_{quat}$). EI MS (70 eV): *m*/*z* (%): 368 (23), 367 ([M]$^+$, 100), 353 (12), 352 ([$C_{21}H_{13}F_3NO$]$^+$, 59), 324 ([$C_{20}H_{13}F_3N$]$^+$, 16), 254 (11), 184 (15). IR: $\tilde{\nu}$ [cm^{-1}] = 3215 (w), 3013 (w), 2955 (w), 2930 (w), 2893 (w), 2837 (w), 1684 (w), 1609 (w), 1512 (m), 1468 (w), 1443 (w), 1406 (w), 1371 (w), 1327 (m), 1300 (m), 1281 (m), 1273 (m), 1250 (m), 1227 (m), 1184 (m), 1157 (m), 1105 (s), 1069 (m), 1030 (m), 1011 (m), 972 (w), 955 (m), 930 (w), 910 (w), 881 (w), 835 (m), 791 (m), 760 (m), 745 (m), 729 (m), 708 (m), 692 (w), 664 (w), 642 (w), 604 (m). Anal. calcd. for $C_{22}H_{16}F_3NO$ [367.4]: C 71.93, H 4.39, N 3.81; Found: 71.78, H 4.51, N 3.66.

1-Phenyl-5-(4-(trifluoromethyl)phenyl)-1*H*-indole (8i)

The synthesis was performed by GP3. After chromatography on silica gel (*n*-hexane/acetone 20:1) and recrystallization from *n*-hexane, **8i** (136 mg, 0.403 mmol, 81%) was isolated as colorless crystals.
Mp 113 °C. ^{1}H NMR (600 MHz, acetone-d$_6$): δ 6.81 (dd, ^{3}J = 3.3 Hz, ^{4}J = 0.8 Hz, 1 H), 7.43-7.46 (m, 1 H), 7.59 (dd, ^{3}J = 8.6 Hz, ^{4}J = 1.8 Hz, 1 H), 7.60-7.63 (m, 2 H), 7.63-7.65 (m, 3 H), 7.69 (d, ^{3}J = 8.6 Hz, 1 H), 7.79 (d, ^{3}J = 8.1 Hz, 2 H), 7.93 (d, ^{3}J = 8.1 Hz, 2 H), 8.04 (dd, ^{4}J = 1.9 Hz, 0.7 Hz, 1 H). ^{13}C NMR (150 MHz, acetone-d$_6$): 105.0 (CH), 111.9 (CH), 120.7 (CH), 122.7 (CH), 124.8 (C$_{quat}$, q, ^{1}J = 270.8 Hz), 125.0 (CH), 126.5 (CH, q, ^{3}J = 3.8 Hz), 127.6 (CH), 128.4 (CH), 128.8 (C$_{quat}$, q, ^{2}J = 32.3 Hz), 130.1 (CH), 130.7 (CH), 131.2 (C$_{quat}$), 132.7 (C$_{quat}$), 136.7 (C$_{quat}$), 140.4 (C$_{quat}$), 146.9 (C$_{quat}$). EI MS (70 eV): *m*/*z* (%): 338 (22), 337 ([M]$^{+}$, 100), 165 ([C$_{13}$H$_9$]$^{+}$, 24), 159 (11). IR: $\tilde{\nu}$ [cm^{-1}] = 3906 (w). 3736 (w), 3726 (w), 3123 (w), 3071 (w), 3044 (w), 3021 (w), 1616 (w), 1599 (w), 1514 (m), 1497 (m), 1470 (w), 1458 (w), 1406 (w), 1373 (w), 1323 (m), 1298 (w), 1267 (w), 1244 (w), 1225 (m), 1155 (m), 1107 (s), 1067 (m), 1003 (w), 953 (w), 883 (w), 843 (m), 837 (w), 806 (s), 791 (m), 733 (s), 691 (s), 638 (m), 625 (s), 608 (m). Anal. calcd. for $C_{21}H_{14}F_3N$ [337.4]: C 74.77, H 4.18, N 4.15; Found C 74.95, H 4.12, N 3.95.

1,5-Bis(4-(trifluoromethyl)phenyl)-1*H*-indole (8j)

The synthesis was performed by GP3. After chromatography on silica gel (*n*-hexane/acetone 20:1) and recrystallization from *n*-hexane, **8j** (142 mg, 0.350 mmol, 70%) was isolated as colorless crystals.
Mp 159 °C. ^{1}H NMR (300 MHz, acetone-d$_6$): δ 6.88 (dd, ^{3}J = 3.4 Hz, ^{4}J = 0.8 Hz, 1 H), 7.64 (dd, ^{3}J = 8.7 Hz, ^{4}J = 1.8 Hz, 1 H), 7.74 (d, ^{3}J = 3.4 Hz, 1 H), 7.78-7.84 (m, 3 H), 7.89-7.94 (m, 3 H), 7.95-8.00 (m, 3 H), 8.07 (dd, ^{4}J = 1.8 Hz, 0.6 Hz, 1 H).^{13}C NMR (75 MHz, acetone-d$_6$): 106.3 (CH), 112.0 (CH), 120.9 (CH), 123.2 (CH), 125.0 (CH), 125.2 (C$_{quat}$, q, ^{1}J = 271.0 Hz), 126.5 (CH, q, ^{3}J = 3.8 Hz), 128.0 (CH, q, ^{3}J = 3.8 Hz), 128.4 (CH), 128.7 (C$_{quat}$, q, ^{2}J = 32.4 Hz), 129.8 (CH), 131.6 (C$_{quat}$), 133.3 (C$_{quat}$), 136.3 (C$_{quat}$), 143.7 (C$_{quat}$), 146.7 (C$_{quat}$). EI MS (70 eV): *m*/*z* (%): 406 (23), 405 ([M]$^{+}$, 100), 183 ([C$_{10}$H$_6$F$_3$]$^{+}$, 10), 165 ([C$_{13}$H$_9$]$^{+}$, 15). IR: $\tilde{\nu}$ [cm^{-1}] = 3119 (w), 1611 (w), 1526 (w), 1470 (w), 1447 (w), 1427 (w), 1406 (w), 1375 (w), 1335 (m), 1321 (s), 1283 (w), 1267 (w), 1238 (w), 1227 (m), 1167 (s), 1153 (m), 1117 (s), 1107 (s), 1067 (s), 1032 (w), 1013 (m), 972 (w), 957 (w), 939 (w), 910 (w), 883 (w), 866 (w), 845 (s), 833 (m), 804 (s), 768 (m), 733 (m), 692 (m), 613 (m). Anal. calcd. for $C_{22}H_{13}F_6N$ [405.3]: C 65.19, H 3.23, N 3.46; Found: C 65.42, H 3.14, N 3.32.

1-(4-Methoxyphenyl)-5-(thiophen-2-yl)-1*H*-indole (8k)

The synthesis was performed by GP3. After chromatography on silica gel (*n*-hexane/acetone 20:1) and recrystallization from *n*-hexane, **8k** (90 mg, 0.295 mmol, 59%) was isolated as yellow crystals.
Mp 90 °C. ^{1}H NMR (600 MHz, acetone-d$_6$): δ 3.89 (s, 3 H), 6.70 (dd, ^3J = 3.2 Hz, ^4J = 0.8 Hz, 1 H), 7.10 (dd, ^3J = 5.1 Hz, 3.5 Hz, 1 H), 7.13-7.16 (m, 2 H), 7.36 (dd, ^3J = 5.1 Hz, ^4J = 1.1 Hz, 1 H), 7.39 (dd, ^3J = 3.6 Hz, ^4J = 1.2 Hz, 1 H), 7.46-7.49 (m, 2 H), 7.49-7.50 (m, 1 H), 7.50-7.53 (m, 2 H), 7.94 (dd, ^4J = 1.8 Hz, 0.7 Hz, 1 H). ^{13}C NMR (150 MHz, acetone-d$_6$): 55.9 (CH_3), 104.1 (CH), 111.6 (CH), 115.8 (CH), 118.9 (CH), 121.6 (CH), 123.0 (CH), 124.6 (CH), 126.6 (CH), 127.7 (C_{quat}), 128.9 (CH), 130.3 (CH), 130.6 (C_{quat}), 133.3 (C_{quat}), 136.7 (C_{quat}), 146.5 (C_{quat}), 159.5 (C_{quat}). EI MS (70 eV): *m*/*z* (%): 306 (22), 305 ($[M]^+$, 100), 291 (10), 290 ($[C_{18}H_{12}NOS]^+$, 48), 262 ($[C_{17}H_{12}NS]^+$, 15), 153 ($[C_{11}H_7N]^+$, 13). IR: $\tilde{\nu}$ [cm^{-1}] = 3071 (w), 2999 (w), 2961 (w), 2930 (w), 2835 (w), 1614 (w), 1508 (m), 1472 (m), 1429 (m), 1377 (w), 1333 (m), 1296 (m), 1283 (m), 1244 (m), 1223 (m), 1204 (m), 1169 (m), 1153 (m), 1107 (m), 1078 (w), 1028 (m), 1007 (w), 982 (w), 955 (m), 930 (w), 874 (m), 826 (m), 804 (s), 777 (m), 760 (m), 725 (s), 714 (m), 698 (m), 677 (s), 627 (m). Anal. calcd. for $C_{19}H_{15}NOS$ [305.4]: C 74.73, H 4.95, N 4.59, S 10.50; Found C 74.76, H 5.02, N 4.63, S 10.21.

10-Hexyl-3-(5-(4-methoxyphenyl)-1*H*-indol-1-yl)-10*H*-phenothiazine (8l)

The synthesis was performed by GP3. After chromatography on silica gel (*n*-hexane/acetone 20:1) and recrystallization from *n*-hexane, **8l** (114 mg, 0.226 mmol, 45%) was isolated as colorless crystals.
Mp 202 °C. ^{1}H NMR (600 MHz, acetone-d$_6$): δ 0.92 (t, ^3J = 7.1 Hz, 3 H), 1.35-1.40 (m, 4 H), 1.51-1.55 (m, 2 H), 1.85-1.92 (m, 2 H), 3.84 (s, 3 H), 4.00 (t, ^3J = 7.0 Hz, 2 H), 6.66 (dd, ^3J = 3.2 Hz, ^4J = 0.8 Hz, 1 H), 6.95 (dd, ^3J = 7.5 Hz, ^4J = 1.2 Hz, 1 H), 6.97-6.99 (m, 2 H), 7.03 (dd, ^3J = 8.2 Hz, ^4J = 1.1 Hz, 1 H), 7.13-7.16 (m, 2 H), 7.21 (ddd, ^3J = 8.2 Hz, 7.3 Hz, ^4J = 1.6 Hz, 1 H), 7.31 (d, ^4J = 2.5 Hz, 1 H), 7.38 (dd, ^3J = 8.6 Hz, ^4J = 2.5 Hz, 1 H), 7.41 (td, ^3J = 3.6 Hz, ^4J = 1.8 Hz, 2 H), 7.53 (td, ^3J = 8.6 Hz, ^4J = 0.8 Hz, 1 H), 7.55-7.58 (m, 2 H), 7.78 (dd, ^4J = 1.8 Hz, 0.7 Hz, 1 H). ^{13}C NMR (150 MHz, acetone-d$_6$): 14.6 (CH_3), 23.6 (CH_2), 27.5 (CH_2), 27.7 (CH_2), 32.4 (CH_2), 48.1 (CH_2), 55.5 (CH_3), 104.6 (CH), 111.4 (CH), 115.0 (CH), 116.6 (CH), 117.0 (CH), 119.6 (CH), 122.5 (CH), 123.5 (CH), 123.5 (CH), 123.9 (CH), 124.9 (C_{quat}), 127.2 (C_{quat}), 128.1 (CH), 128.5 (CH), 128.8 (CH), 129.3 (CH), 130.8 (C_{quat}), 134.1 (C_{quat}), 135.3 (C_{quat}), 135.4 (C_{quat}), 135.9 (C_{quat}), 144.6 (C_{quat}), 145.8 (C_{quat}), 159.5 (C_{quat}). EI MS (70 eV): *m*/*z* (%): 505 (16), 504 ($[M]^+$, 45), 420 (16), 419 ($[C_{27}H_{19}N_2OS]^+$, 52), 376 (13), 329 ($[C_{21}H_{15}NOS]^+$, 14), 304 (10), 179 ($[C_{13}H_9N]^+$, 11), 91 (16), 84 (14), 69 ($[C_4H_5O]^+$, 34), 57 ($[C_4H_9]^+$, 44), 56 ($[C_3H_4O]^+$, 100), 55 (35). IR: $\tilde{\nu}$ [cm^{-1}] = 3555 (w), 2947 (w), 2932 (w), 2909 (w), 2868 (w), 2847 (w), 2832 (w), 1605 (w), 1499 (m), 1464 (s), 1439 (m), 1373 (w), 1333 (m), 1300 (m), 1269 (m), 1236 (m), 1223 (m), 1177 (m), 1153 (m), 1146 (m), 1105 (m), 1078 (w), 1040 (m), 1022 (m), 1007 (w), 978 (w), 910 (w), 887 (m), 843 (m), 826 (m), 799 (s), 789 (m), 758 (s), 725 (m), 704 (m), 681 (m), 627 (m). Anal. calcd. for $C_{33}H_{32}N_2OS$ [504.7]: C 78.54, H 6.39, N 5.55, S 6.35; Found: C 78.34, H 6.12, N 5.34, S 6.11.

1-(4-Methoxyphenyl)-5-(naphthalen-1-yl)-1*H*-indole (8m)

The synthesis was performed by GP3. After chromatography on silica gel (*n*-hexane/acetone 20:1) and recrystallization from *n*-hexane, **8m** (50 mg, 0.143 mmol, 29%) was isolated as yellow crystals.
Mp 126 °C. ^{1}H NMR (300 MHz, acetone-d$_6$): δ 3.91 (s, 3 H), 6.75 (dd, ^3J = 3.2 Hz, ^4J = 0.8 Hz, 1 H), 7.14-7.21 (m, 2 H), 7.31 (dd, ^3J = 8.5 Hz, ^4J = 1.7 Hz, 1 H), 7.41-7.53 (m, 3 H), 7.53-7.62 (m, 5 H), 7.75 (dd, ^4J = 1.7 Hz, 0.6 Hz, 1 H), 7.89-7.94 (m, 1 H), 7.95-8.00 (m, 2 H). ^{13}C NMR (75 MHz, acetone-d$_6$): 55.9 (CH$_3$), 104.0 (CH), 110.9 (CH), 115.8 (CH), 123.0 (CH), 125.3 (CH), 126.3 (CH), 126.5 (CH), 126.6 (CH), 126.7 (CH), 127.0 (CH), 128.0 (CH), 128.0 (CH), 129.1 (CH), 130.0 (CH), 130.3 (C$_{quat}$), 133.0 (C$_{quat}$), 133.4 (C$_{quat}$), 133.5 (C$_{quat}$), 135.0 (C$_{quat}$), 136.5 (C$_{quat}$), 142.1 (C$_{quat}$), 159.4 (C$_{quat}$). EI MS (70 eV): *m*/*z* (%): 350 (26), 349 ([M]$^+$, 100), 348 (22), 334 ([C$_{24}$H$_{16}$NO]$^+$, 21), 304 (10), 152 ([C$_{11}$H$_6$N]$^+$, 18). IR: $\tilde{\nu}$ [cm^{-1}] = 3051 (w), 2959 (w), 2936 (w), 2905 (w), 2837 (w), 1612 (w), 1591 (w), 1516 (s), 1477 (m), 1460 (m), 1443 (m), 1429 (w), 1395 (m), 1335 (m), 1302 (m), 1279 (m), 1240 (s), 1184 (m), 1159 (m), 1144 (w), 1123 (m), 1107 (m), 1090 (w), 1024 (s), 955 (m), 905 (w), 889 (m), 866 (w), 820 (m), 799 (s), 775 (s), 762 (m), 745 (m), 721 (s), 708 (m), 683 (m), 662 (m), 648 (m), 633 (m). Anal. calcd. for C$_{25}$H$_{19}$NO [349.4]: C 85.93, H 5.48, N 4.01; Found C 86.03, H 5.64, N 4.10.

5-(4-Methoxyphenyl)-1-(naphthalen-1-yl)-1*H*-indole (8n)

The synthesis was performed by GP3. After chromatography on silica gel (*n*-hexane/acetone 20:1) and recrystallization from *n*-hexane, **8n** (102 mg, 0.292 mmol, 58%) was isolated as colorless crystals.
Mp 92 °C. ^{1}H NMR (600 MHz, acetone-d$_6$): δ 03.84 (s, 3 H), 6.83 (dd, ^3J = 3.1 Hz, ^4J = 0.8 Hz, 1 H), 6.99 (d, ^3J = 8.5 Hz, 1 H), 7.00-7.03 (m, 2 H), 7.36 (dd, ^3J = 8.5 Hz, ^4J = 1.8 Hz, 1 H), 7.39 (dd, ^3J = 8.5 Hz, ^4J = 0.7 Hz, 1 H), 7.50 (ddd, ^3J = 8.1 Hz, 6.8 Hz, ^4J = 1.2 Hz, 1 H), 7.53 (d, ^3J = 3.1 Hz, 1 H), 7.60-7.64 (m, 3 H), 7.66 (dd, ^3J = 7.2 Hz, ^4J = 1.2 Hz, 1 H), 7.71 (dd, ^3J = 8.2 Hz, 7.3 Hz, 1 H), 7.93 (d, ^4J = 1.3 Hz, 1 H), 8.11 (dd, ^3J = 8.2 Hz, 5.2 Hz, 2 H). ^{13}C NMR (150 MHz, acetone-d$_6$): 55.6 (CH$_3$), 104.1 (CH), 111.6 (CH), 115.0 (CH), 119.5 (CH), 122.4 (CH), 123.8 (CH), 126.1 (CH), 126.7 (CH), 127.6 (CH), 128.0 (CH), 128.9 (CH), 129.3 (CH), 129.5 (CH), 130.2 (C$_{quat}$), 131.4 (CH), 131.4 (C$_{quat}$), 134.1 (C$_{quat}$), 135.6 (C$_{quat}$), 135.6 (C$_{quat}$), 136.8 (C$_{quat}$), 138.2 (C$_{quat}$), 159.7 (C$_{quat}$). EI MS (70 eV): *m*/*z* (%): 350 (25), 349 ([M]$^+$, 100), 348 (13), 335 (11), 334 (C$_{24}$H$_{16}$NO]$^+$, 39), 306 ([C$_{23}$H$_{16}$N]$^+$, 18), 304 (14), 175 (11), 152 ([C$_{11}$H$_6$N]$^+$, 21), 151 ([C$_{12}$H$_7$]$^+$, 14), 139 ([C$_{10}$H$_5$N]$^+$, 11). IR: $\tilde{\nu}$ [cm^{-1}] = 2995 (w), 2955 (w), 2903 (w), 2835 (w), 1607 (w), 1597 1271 (m), (w), 1576 (w), 1508 (m), 1474 (m), 1458 (m), 1441 (m), 1402 (m), 1335 (w), 1298 (w), 1244 (s), 1233 (m), 1180 (m), 1152 (w), 1132 (w), 1111 (w), 1088 (w), 1040 (m), 1017 (w), 1007 (w), 934 (m), 912 (w), 878 (w), 868 (w), 833 (m), 799 (s), 772 (s), 760 (m), 719 (s), 652 (m), 642 (m), 625 (w). Anal. calcd. for C$_{25}$H$_{19}$NO [349.4]: C 85.93, H 5.48, N 4.01; Found C 86.05, H 5.66, N 3.97.

4. NMR spectra

4.1 ^{1}H and ^{13}C NMR spectra of 3,10-diaryl phenothiazines 4

^{1}H NMR spectrum of 3,10-Bis(4-methoxyphenyl)-10*H*-phenothiazine (4a) (acetone-d_6, 300 MHz, 293 K)

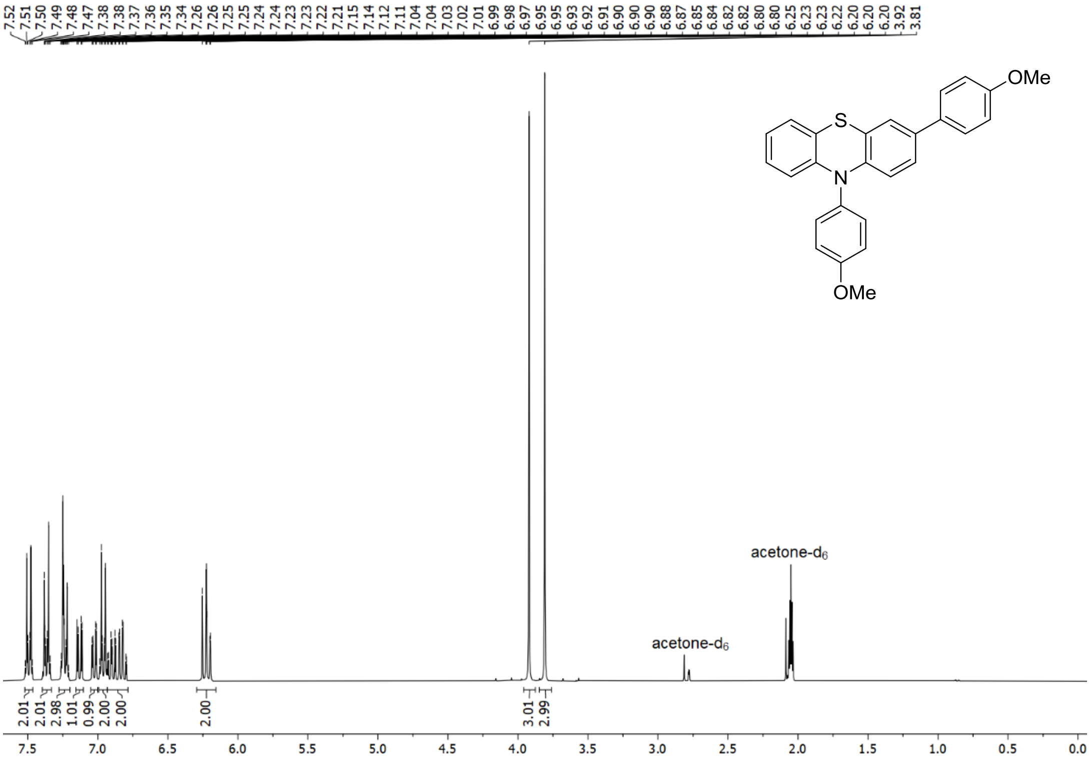

^{13}C NMR spectrum of 3,10-Bis(4-methoxyphenyl)-10*H*-phenothiazine (4a) (acetone-d_6, 75 MHz, 293 K)

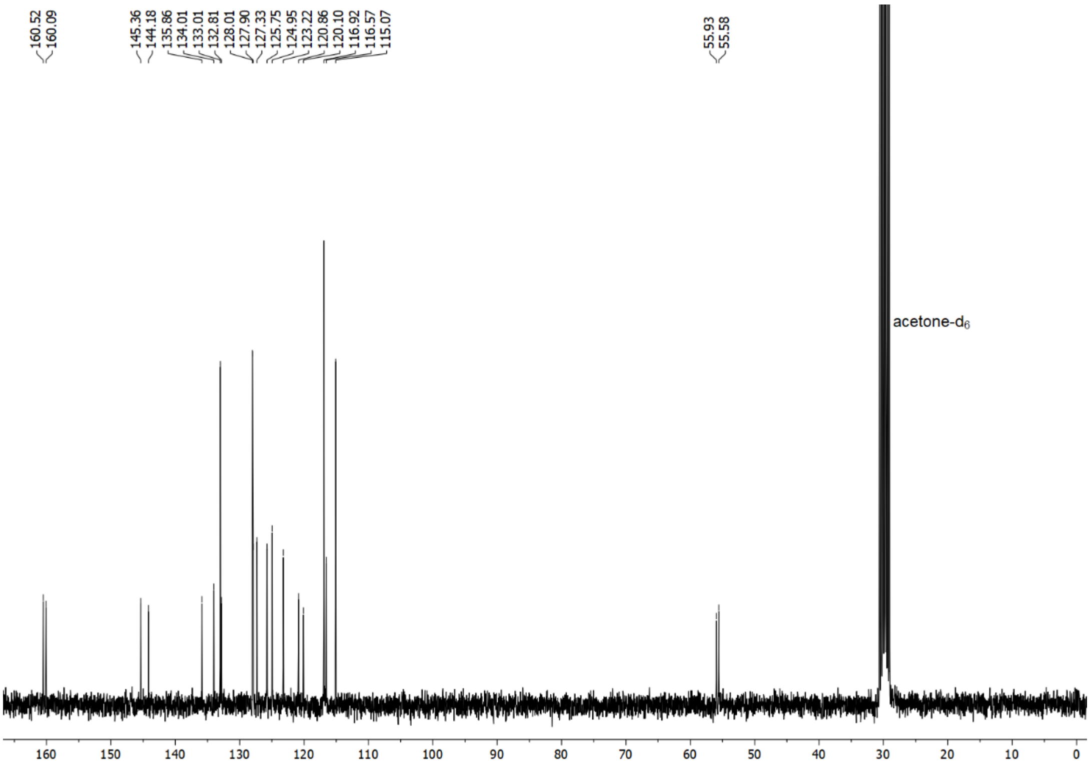

^{1}H NMR spectrum of 3-(4-Methoxyphenyl)-10-phenyl-10*H*-phenothiazine (4b) (acetone-d$_6$, 300 MHz, 293 K)

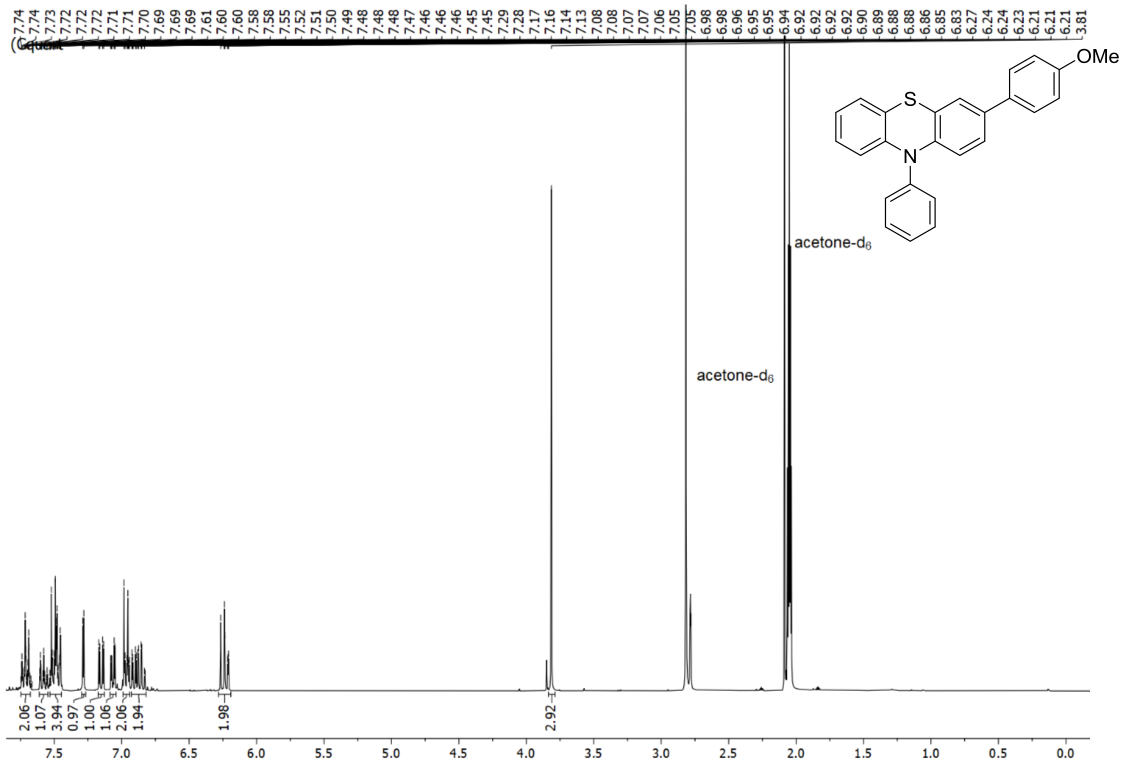

^{13}C NMR spectrum of 3-(4-Methoxyphenyl)-10-phenyl-10*H*-phenothiazine (4b) (acetone-d$_6$, 75 MHz, 293 K)

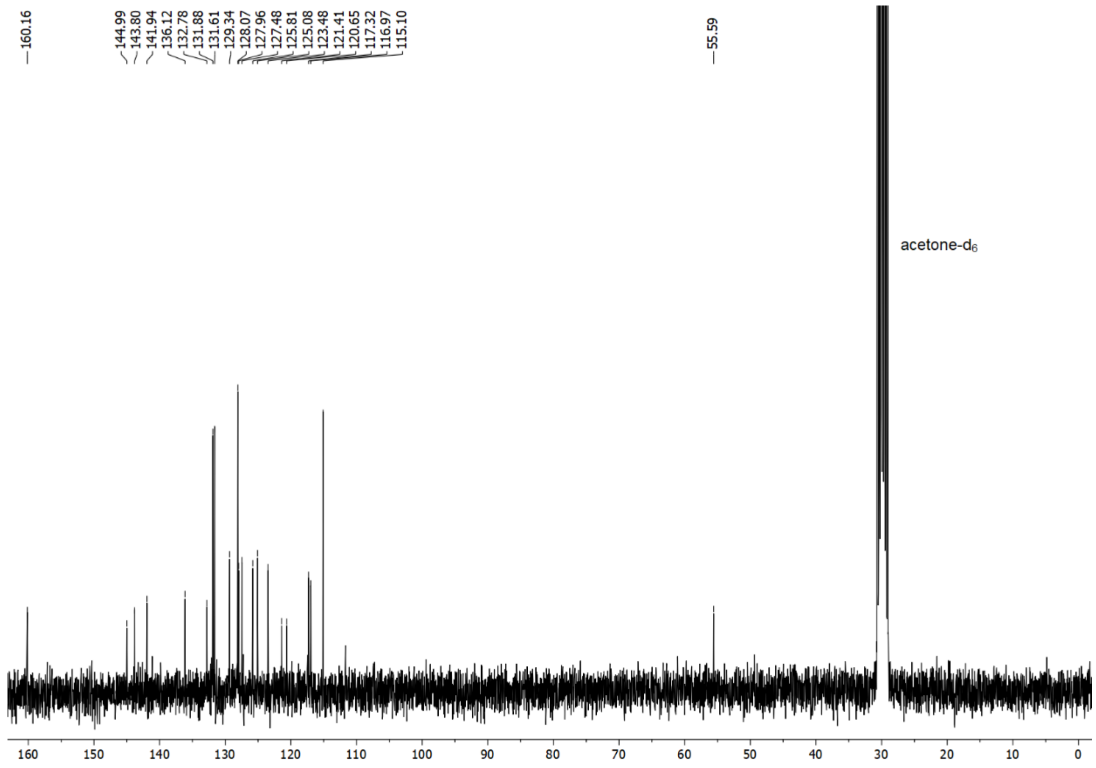

^{1}H NMR spectrum of 4-(3-(4-Methoxyphenyl)-10*H*-phenothiazin-10-yl)benzonitrile (4c) (acetone-d_6, 500 MHz, 293 K)

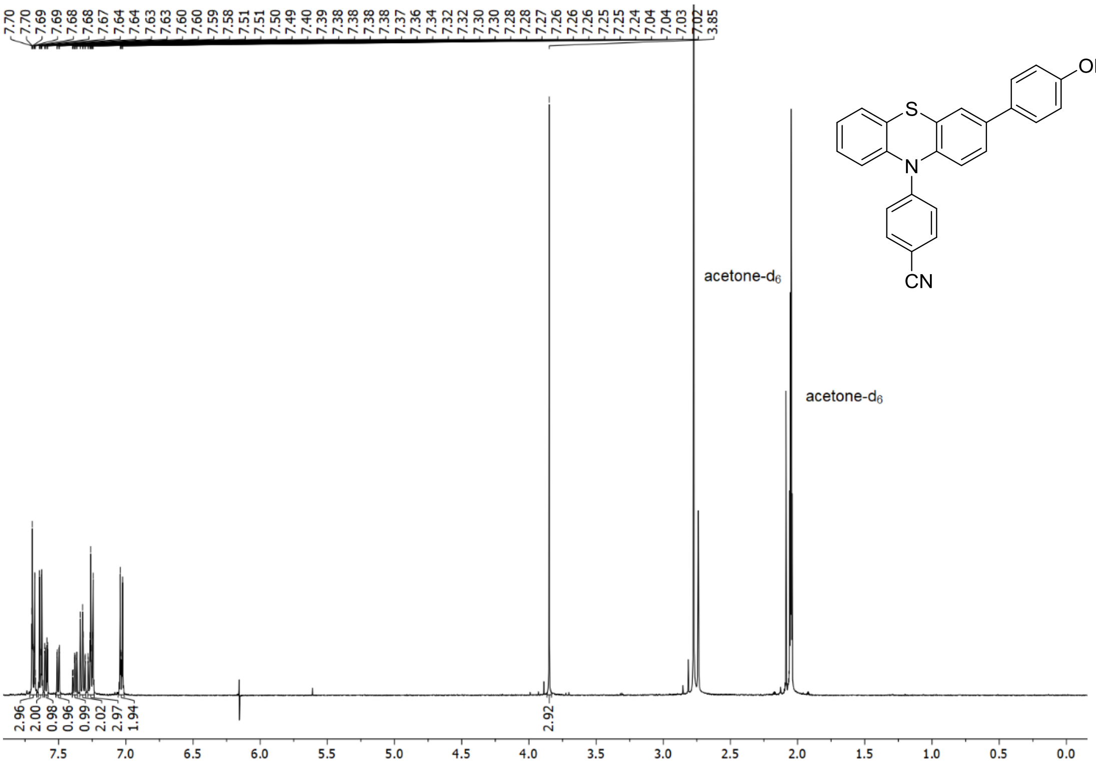

^{13}C NMR spectrum of 4-(3-(4-Methoxyphenyl)-10*H*-phenothiazin-10-yl)benzonitrile (4c) (acetone-d_6, 125 MHz, 293 K)

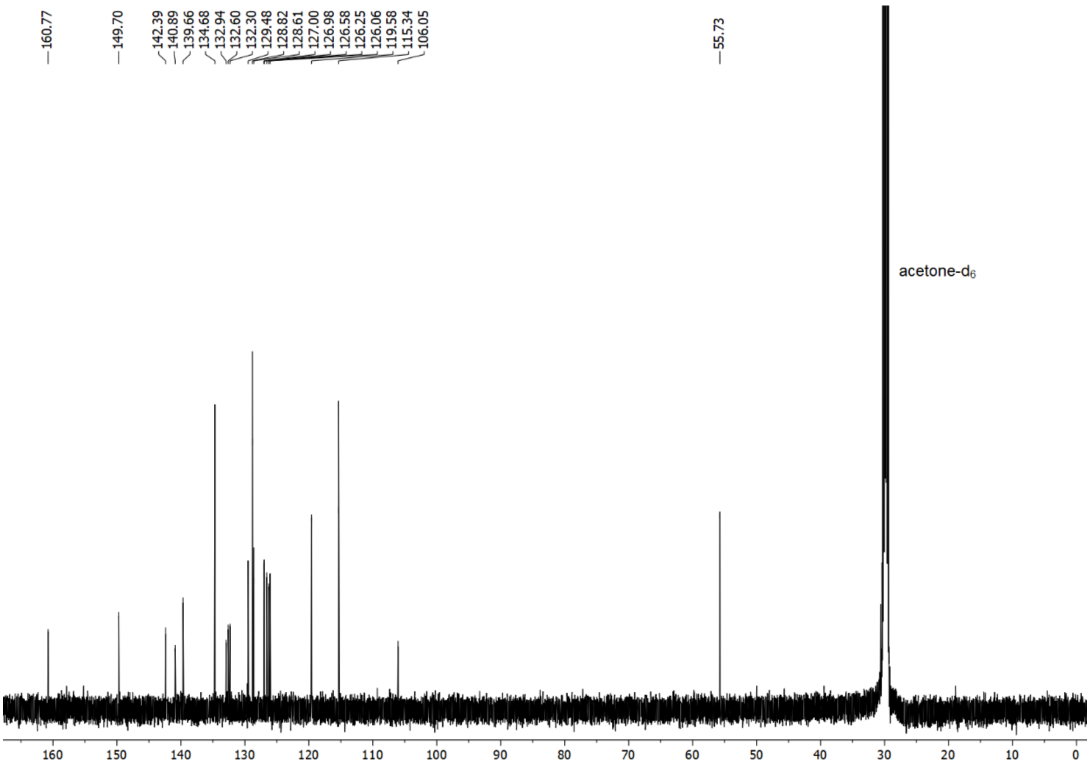

¹H NMR spectrum of 10-(4-Methoxyphenyl)-3-phenyl-10*H*-phenothiazine (4d) (acetone-d$_6$, 600 MHz, 293 K)

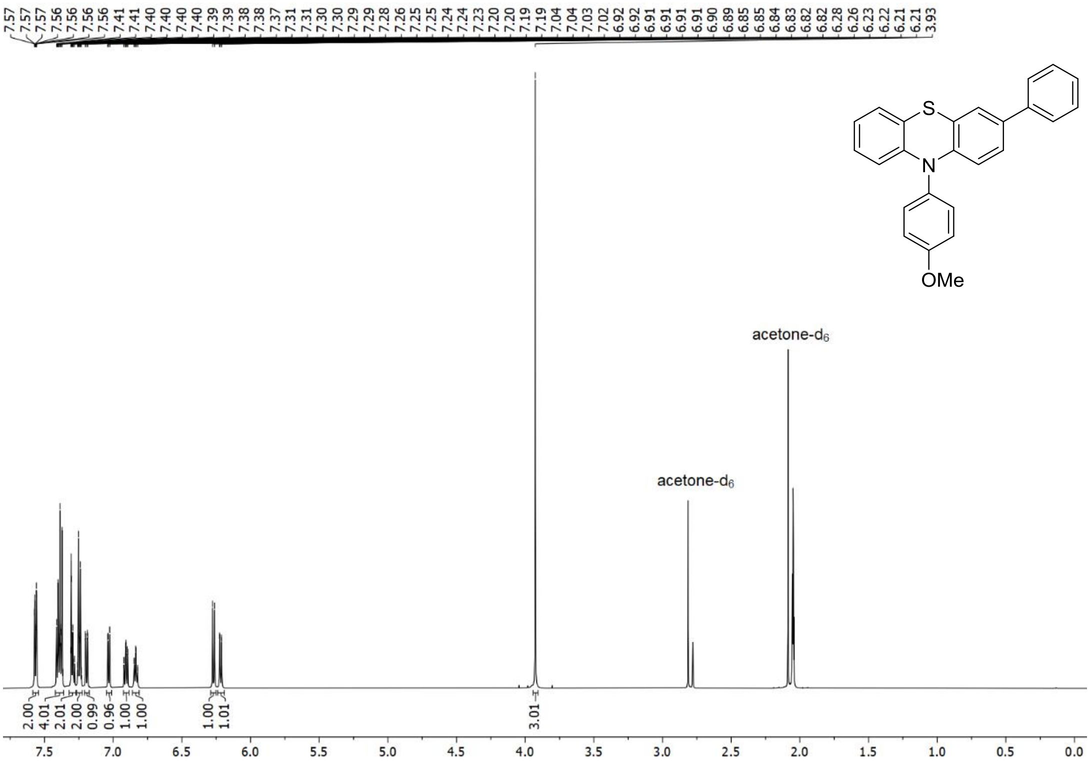

¹³C NMR spectrum of 10-(4-Methoxyphenyl)-3-phenyl-10*H*-phenothiazine (4d) (acetone-d$_6$, 150 MHz, 293 K)

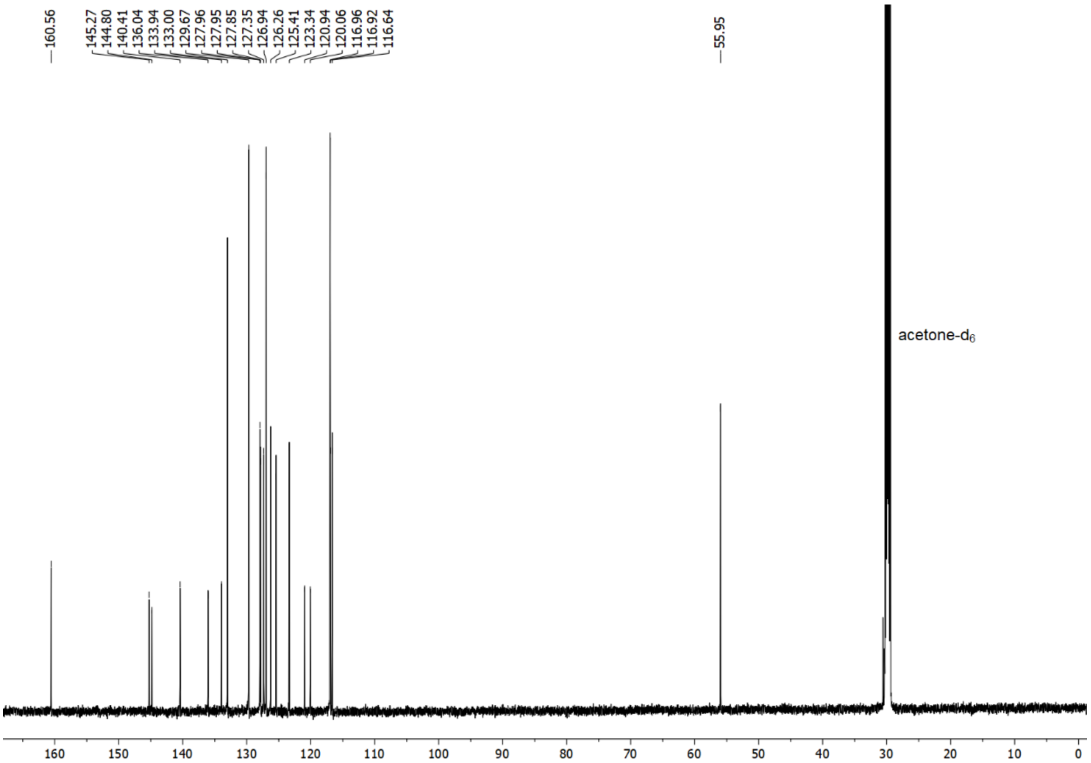

^{1}H NMR spectrum of 3,10-Diphenyl-10*H*-phenothiazine (4e) (acetone-d$_6$, 600 MHz, 293 K)

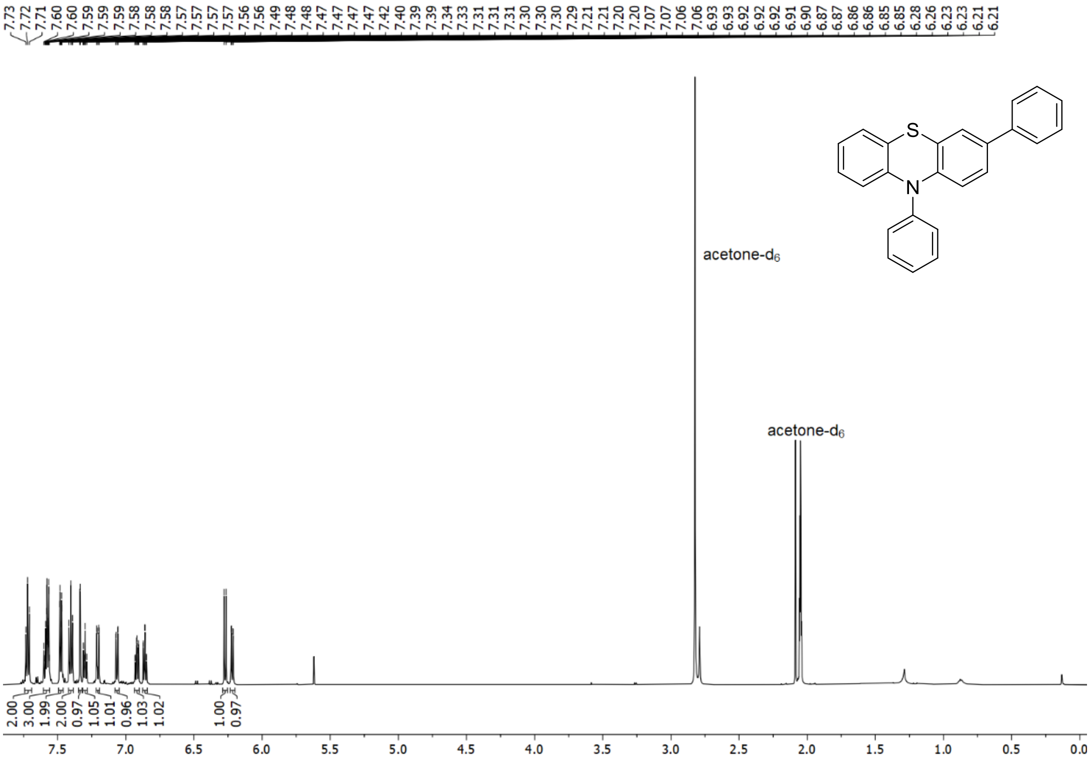

^{13}C NMR spectrum of 3,10-Diphenyl-10*H*-phenothiazine (4e) (acetone-d$_6$, 150 MHz, 293 K)

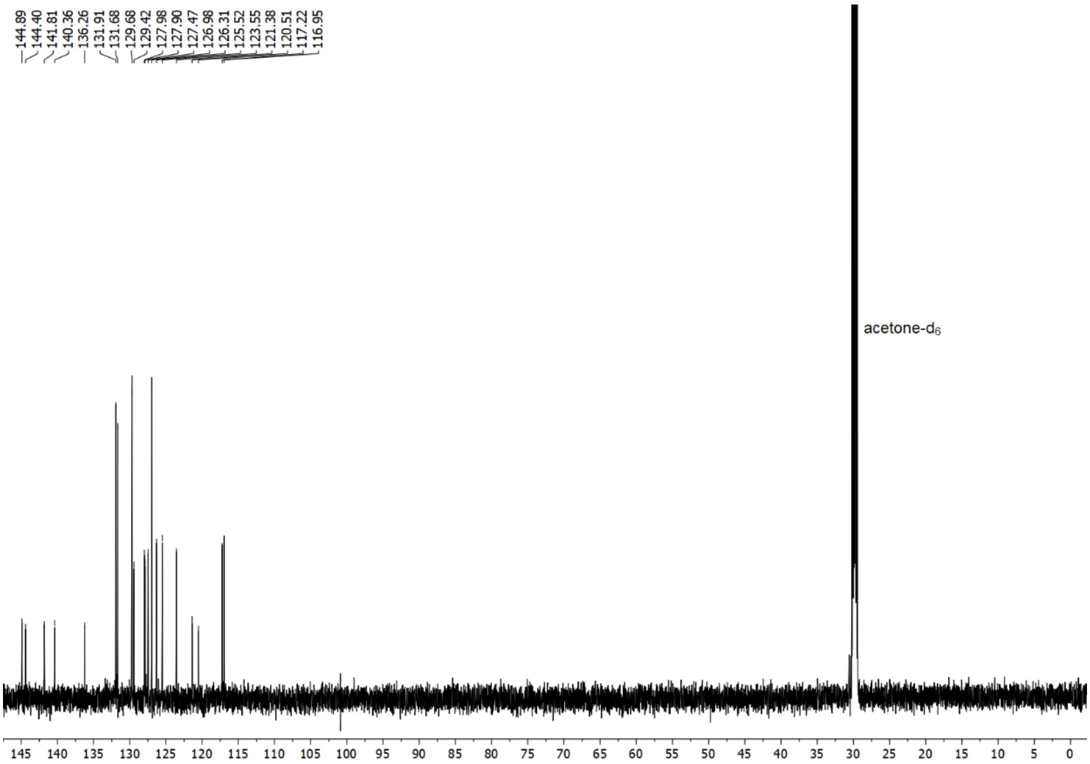

^{1}H NMR spectrum of 4-(3-Phenyl-10*H*-phenothiazin-10-yl)benzonitrile (4f) (acetone-d$_6$, 500 MHz, 293 K)

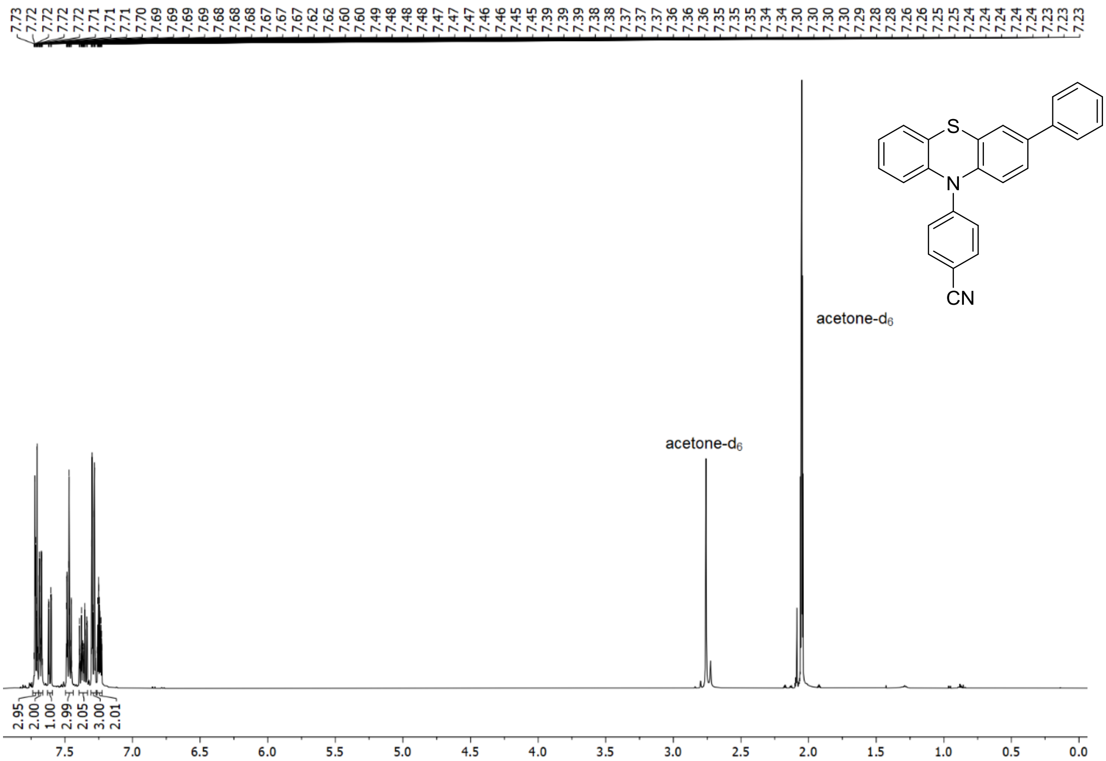

^{13}C NMR spectrum of 4-(3-Phenyl-10*H*-phenothiazin-10-yl)benzonitrile (4f) (acetone-d$_6$, 125 MHz, 293 K)

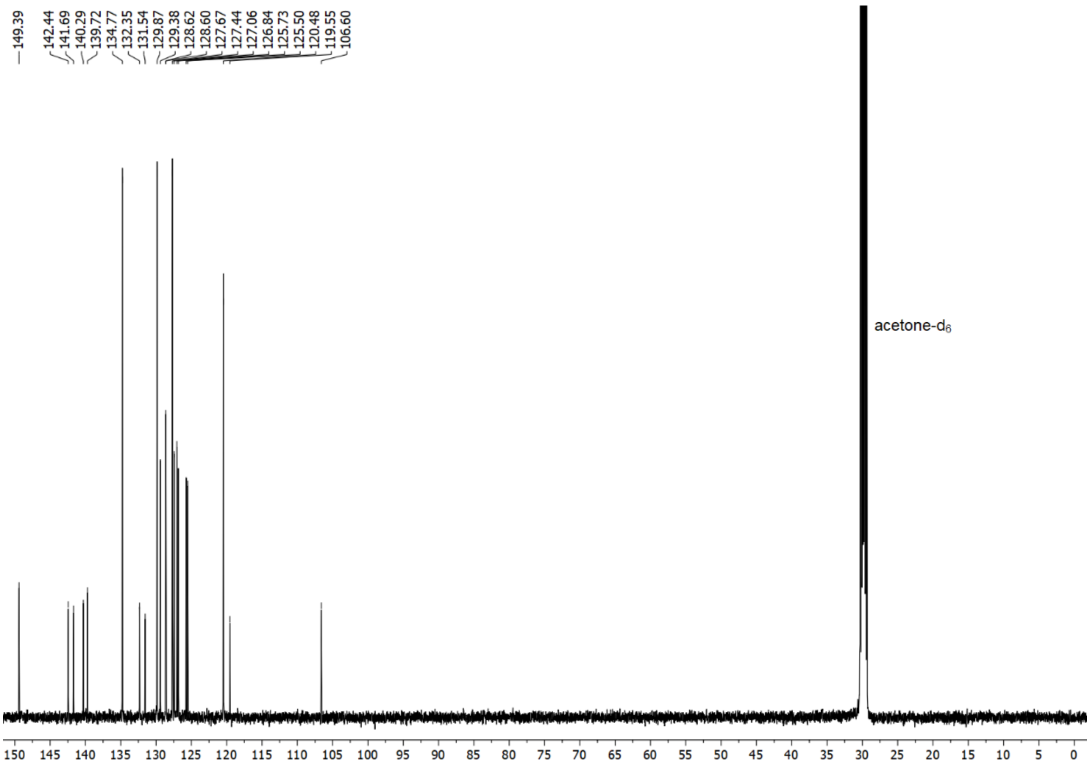

^{1}H NMR spectrum of 10-(4-Methoxyphenyl)-3-(4-(trifluoromethyl)phenyl)-10*H*-phenothiazine (4g) (acetone-d$_6$, 600 MHz, 293 K)

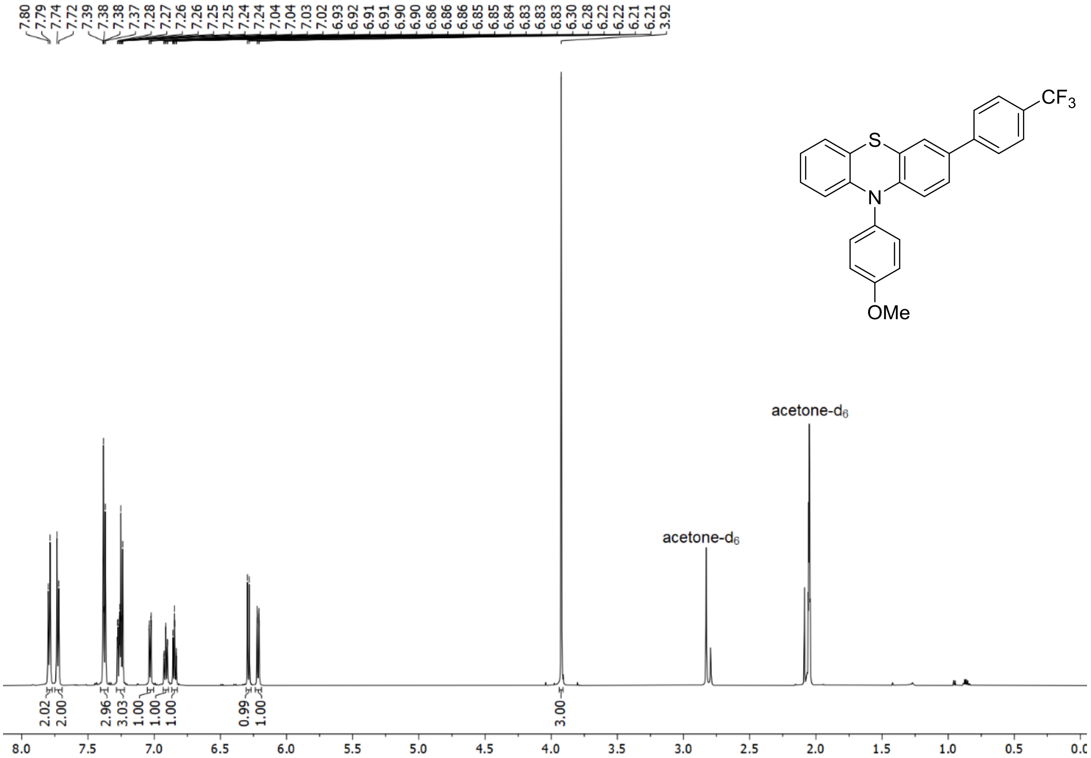

^{13}C NMR spectrum of 10-(4-Methoxyphenyl)-3-(4-(trifluoromethyl)phenyl)-10*H*-phenothiazine (4g) (acetone-d$_6$, 150 MHz, 293 K)

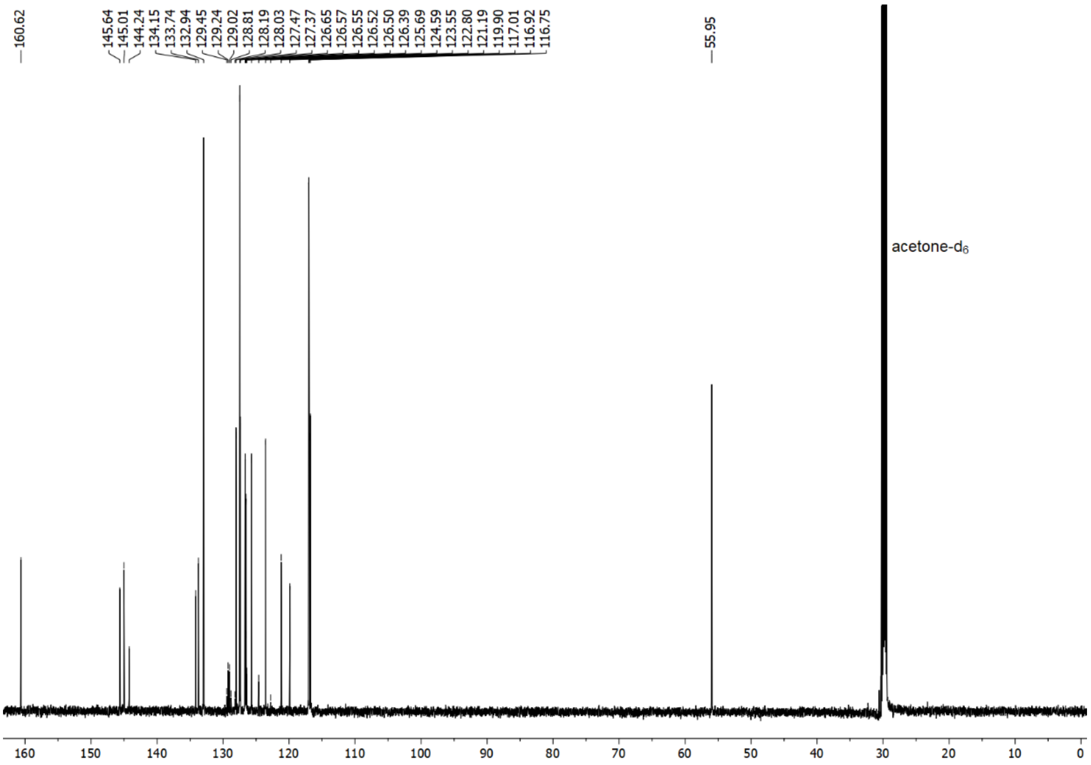

^{1}H NMR spectrum of 10-Phenyl-3-(4-(trifluoromethyl)phenyl)-10*H*-phenothiazine (4h) (acetone-d_6, 600 MHz, 293 K)

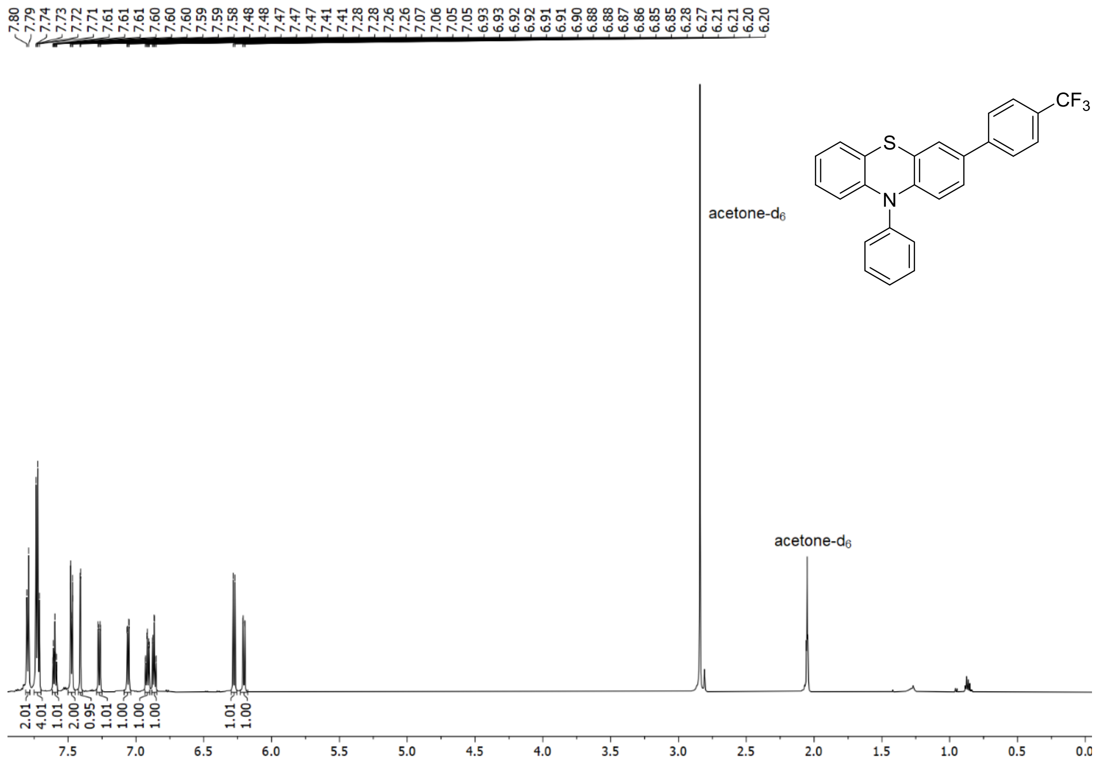

^{13}C NMR spectrum of 10-Phenyl-3-(4-(trifluoromethyl)phenyl)-10*H*-phenothiazine (4h) (acetone-d_6, 150 MHz, 293 K)

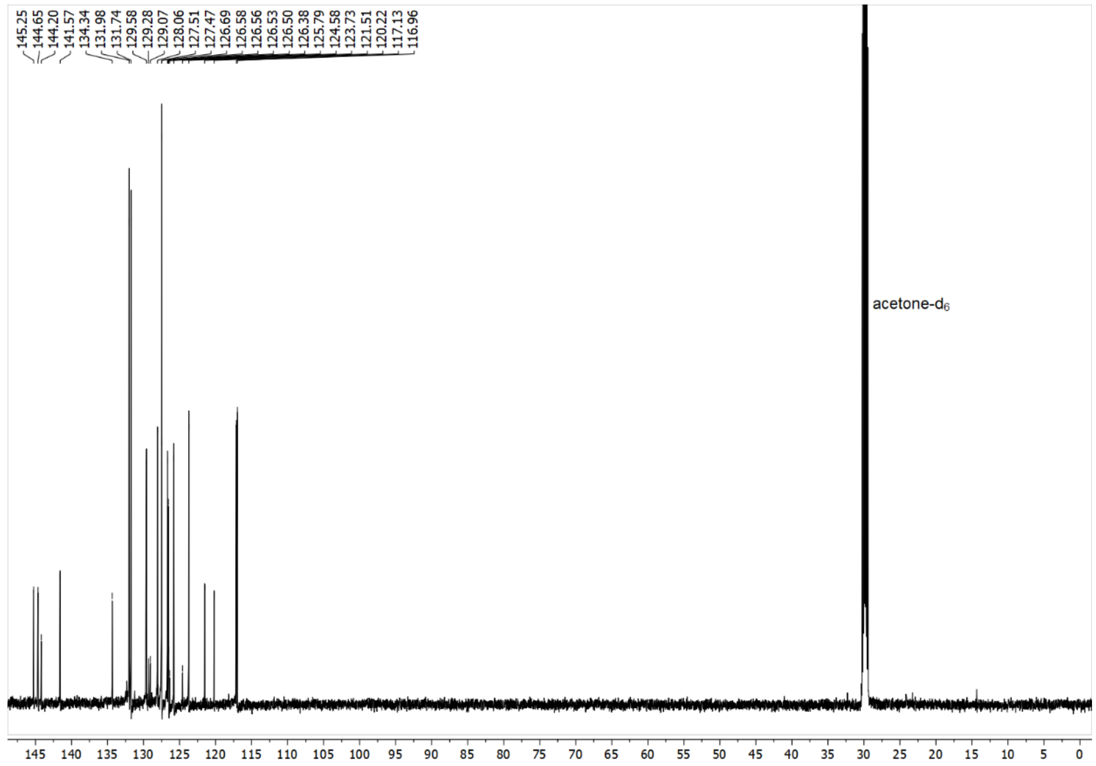

^{1}H NMR spectrum of 4-(3-(4-(Trifluoromethyl)phenyl)-10*H*-phenothiazin-10-yl)benzonitrile (4i) (acetone-d$_6$, 600 MHz, 293 K)

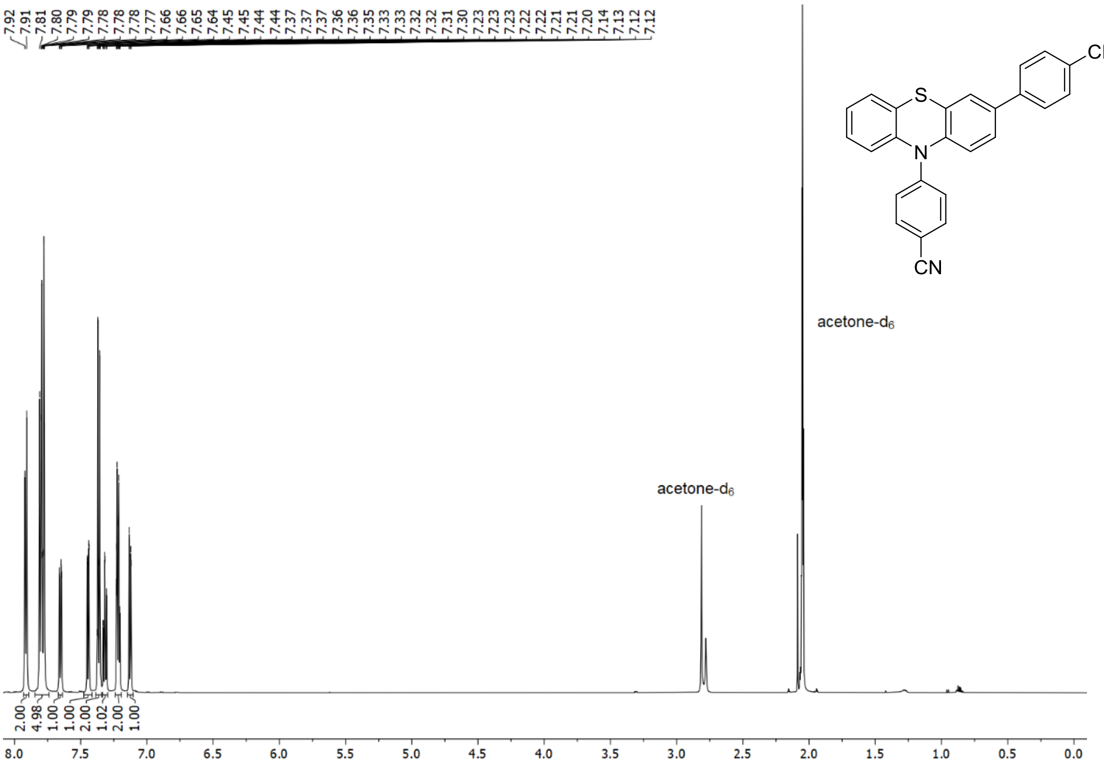

^{13}C NMR spectrum of 4-(3-(4-(Trifluoromethyl)phenyl)-10*H*-phenothiazin-10-yl)benzonitrile (4i) (acetone-d$_6$, 150 MHz, 293 K)

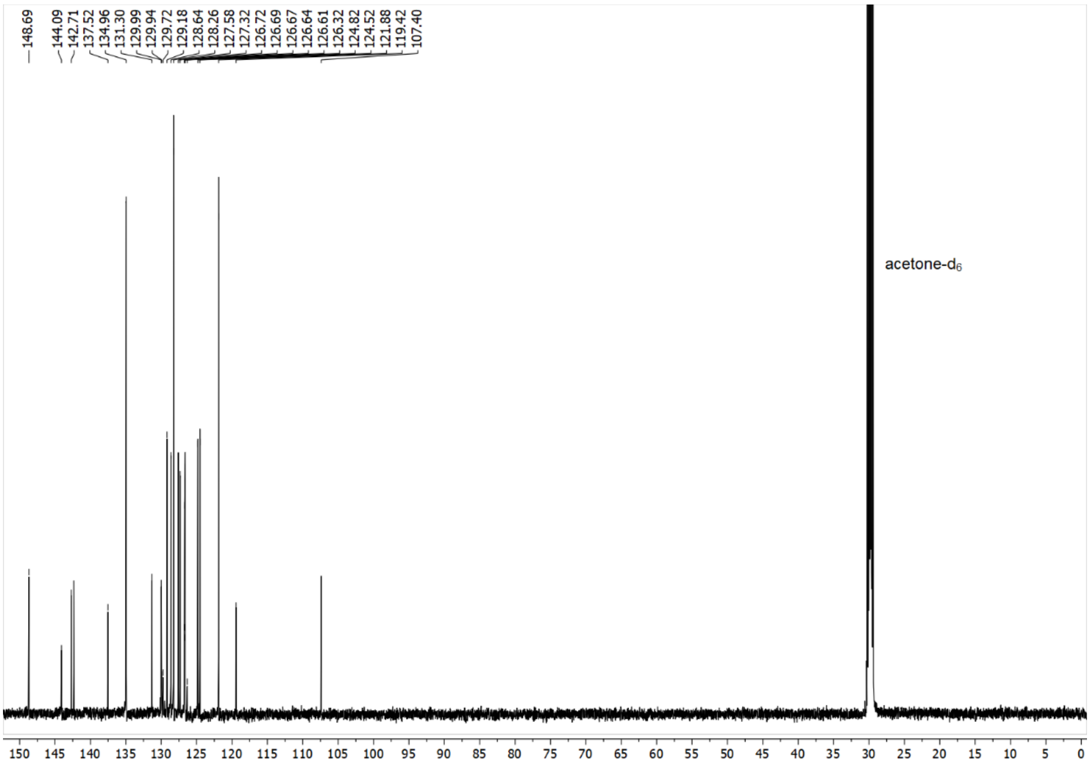

^{1}H NMR spectrum of 10-(4-Methoxyphenyl)-3-(thiophen-2-yl)-10*H*-phenothiazine (4j) (acetone-d_6, 600 MHz, 293 K)

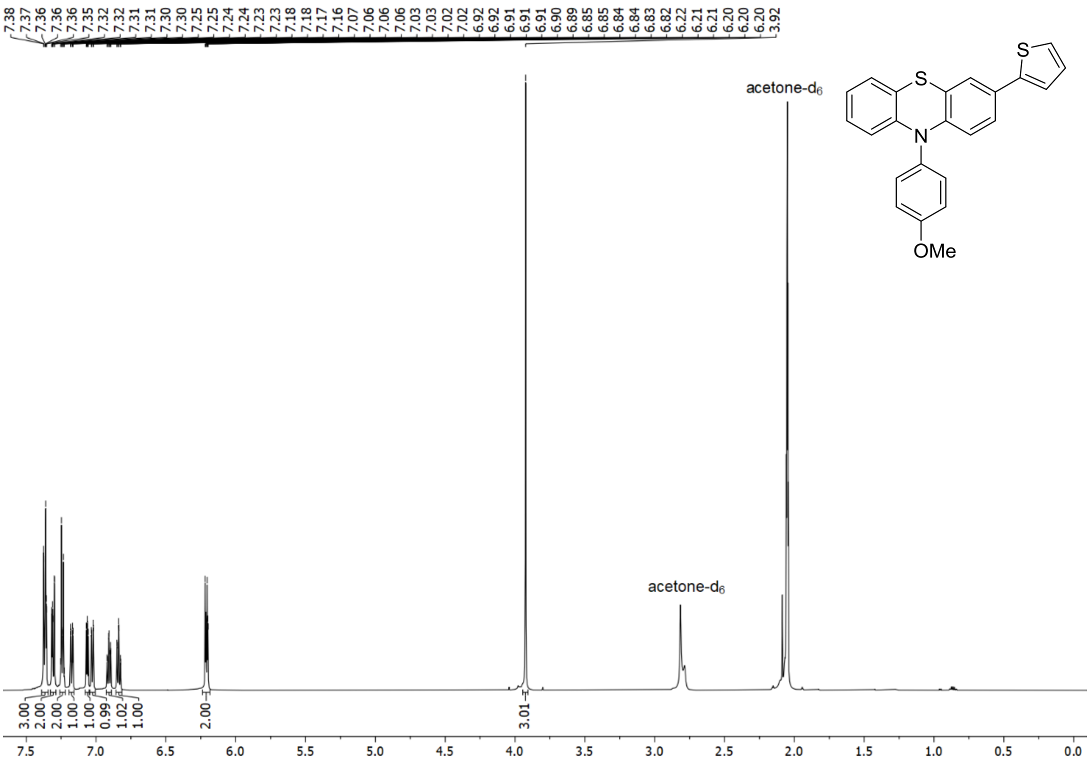

^{13}C NMR spectrum of 10-(4-Methoxyphenyl)-3-(thiophen-2-yl)-10*H*-phenothiazine (4j) (acetone-d_6, 150 MHz, 293 K)

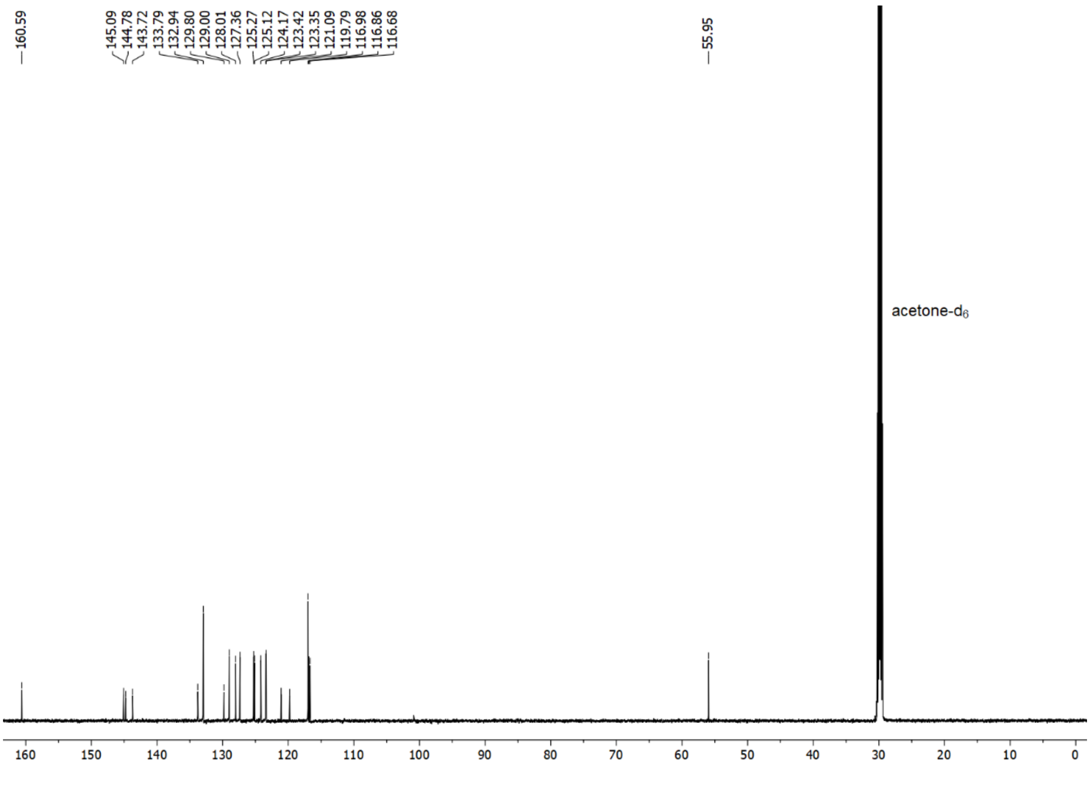

^{1}H NMR spectrum of 3-(4-Methoxyphenyl)-10-(pyridin-4-yl)-10*H*-phenothiazine (4k) (acetone-d$_6$, 300 MHz, 293 K)**

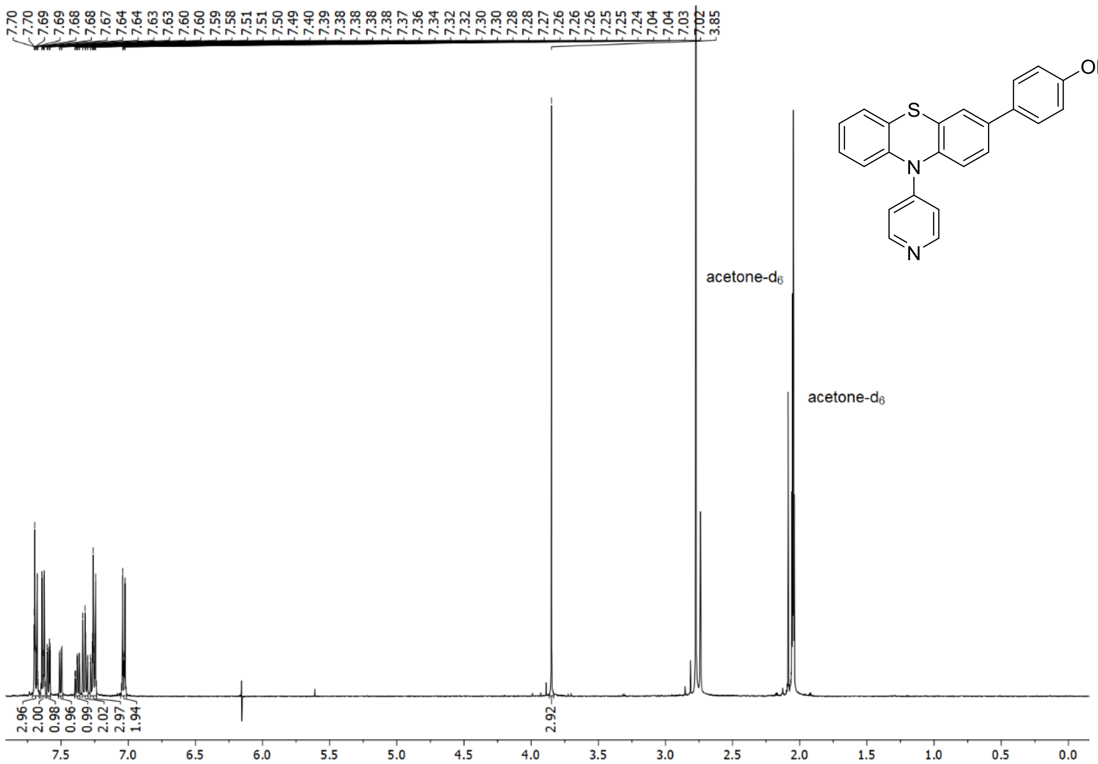

^{13}C NMR spectrum of 3-(4-Methoxyphenyl)-10-(pyridin-4-yl)-10*H*-phenothiazine (4k) (acetone-d$_6$, 75 MHz, 293 K)

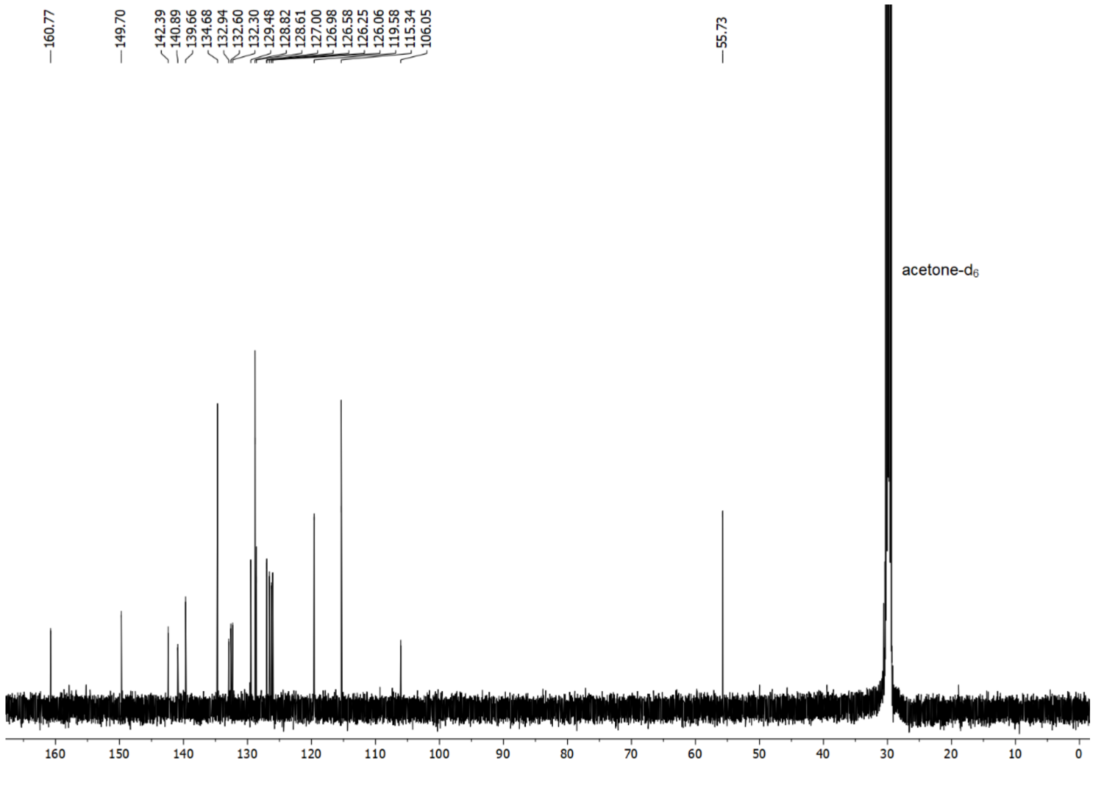

^{1}H NMR spectrum of 10-(4-Methoxyphenyl)-3-(naphthalen-1-yl)-10*H*-phenothiazine (4l) (acetone-d$_6$, 300 MHz, 293 K)

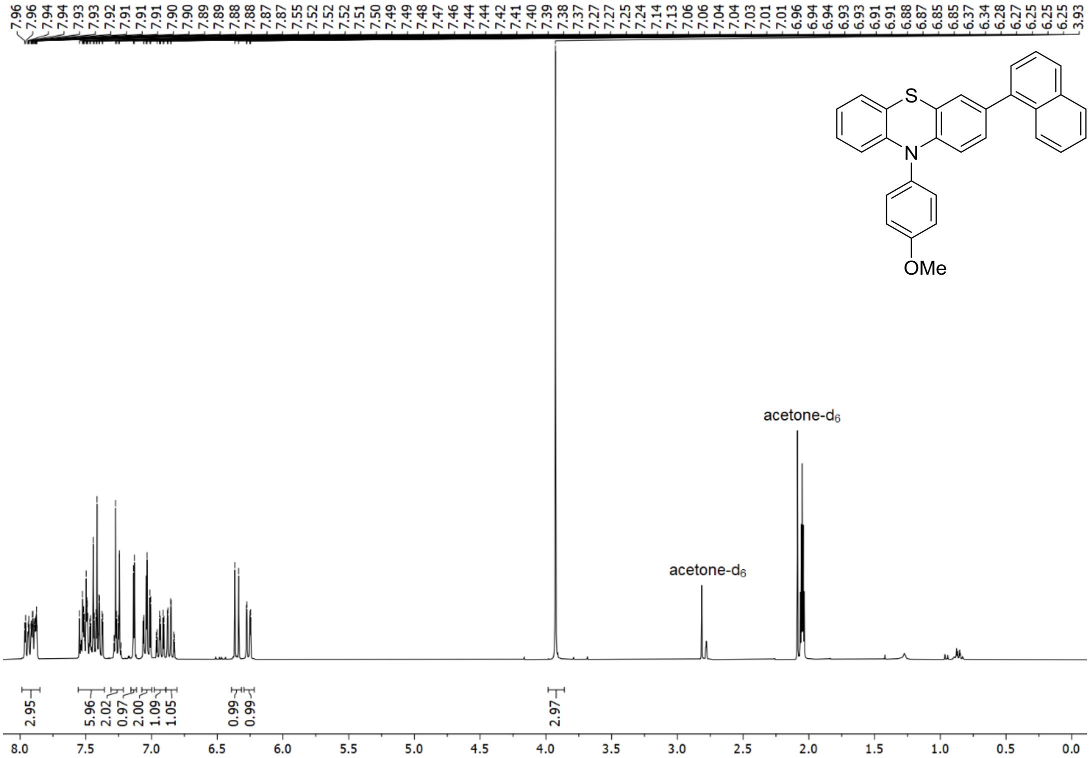

^{13}C NMR spectrum of 10-(4-Methoxyphenyl)-3-(naphthalen-1-yl)-10*H*-phenothiazine (4l) (acetone-d$_6$, 75 MHz, 293 K)

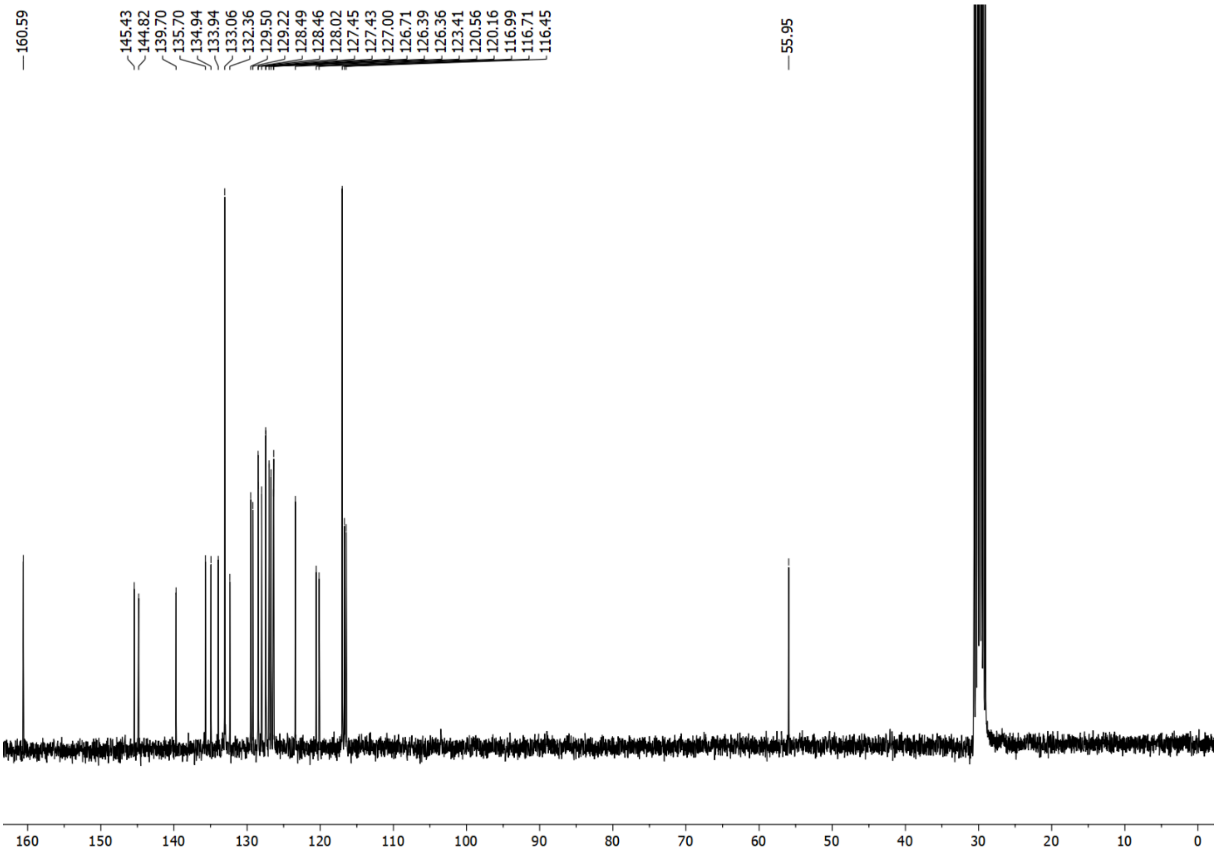

^{1}H NMR spectrum of 3-(4-Methoxyphenyl)-10-(naphthalen-1-yl)-10*H*-phenothiazine (4m) (acetone-d$_6$, 300 MHz, 293 K)

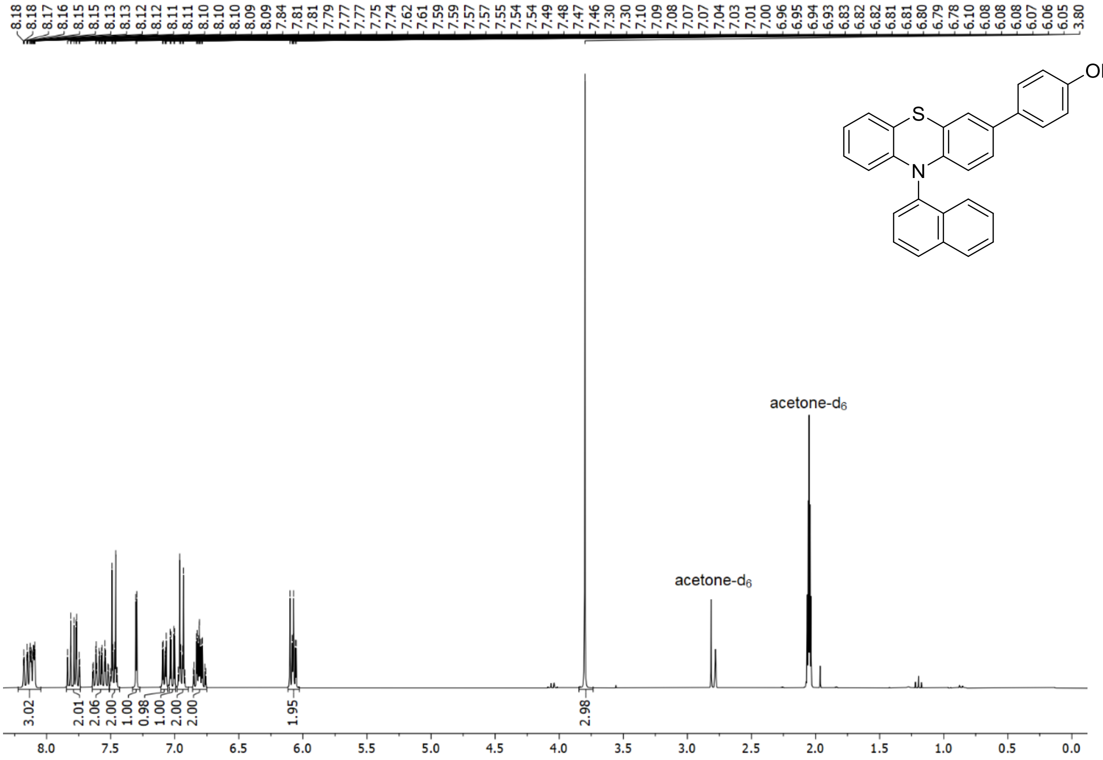

^{13}C NMR spectrum of 3-(4-Methoxyphenyl)-10-(naphthalen-1-yl)-10*H*-phenothiazine (4m) (acetone-d$_6$, 75 MHz, 293 K)

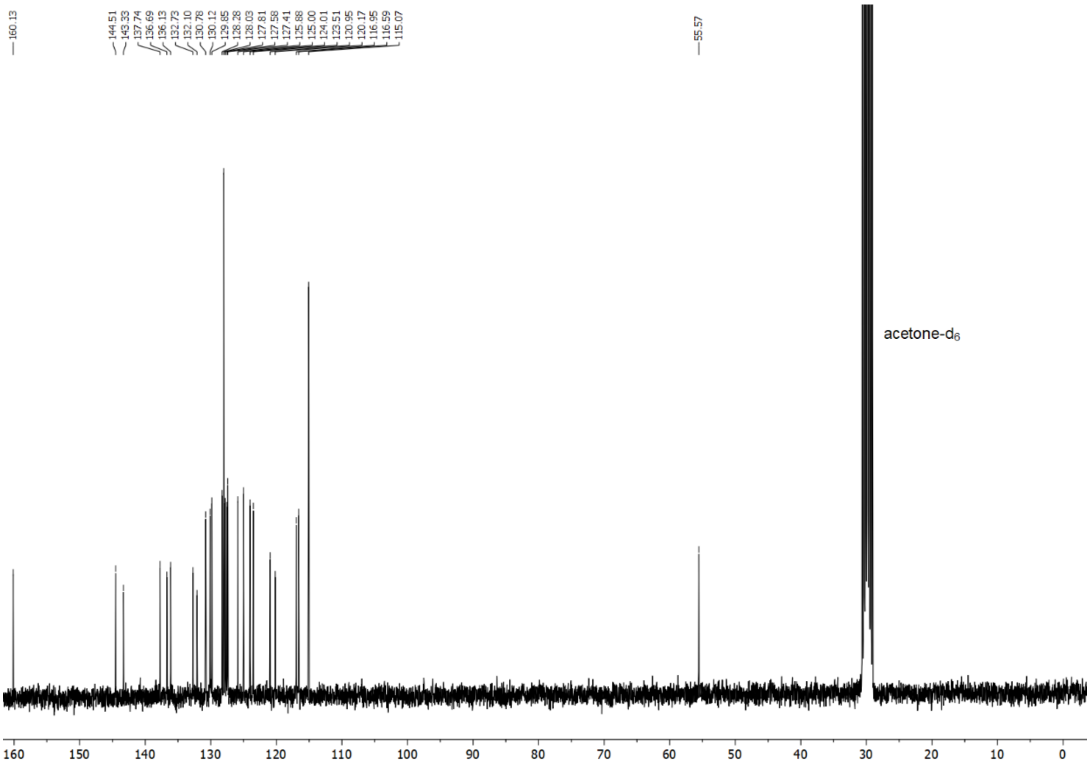

^{1}H NMR spectrum of 10-(4-Methoxyphenyl)-3-(*o*-tolyl)-10*H*-phenothiazine (4n) (acetone-d$_6$, 300 MHz, 293 K)

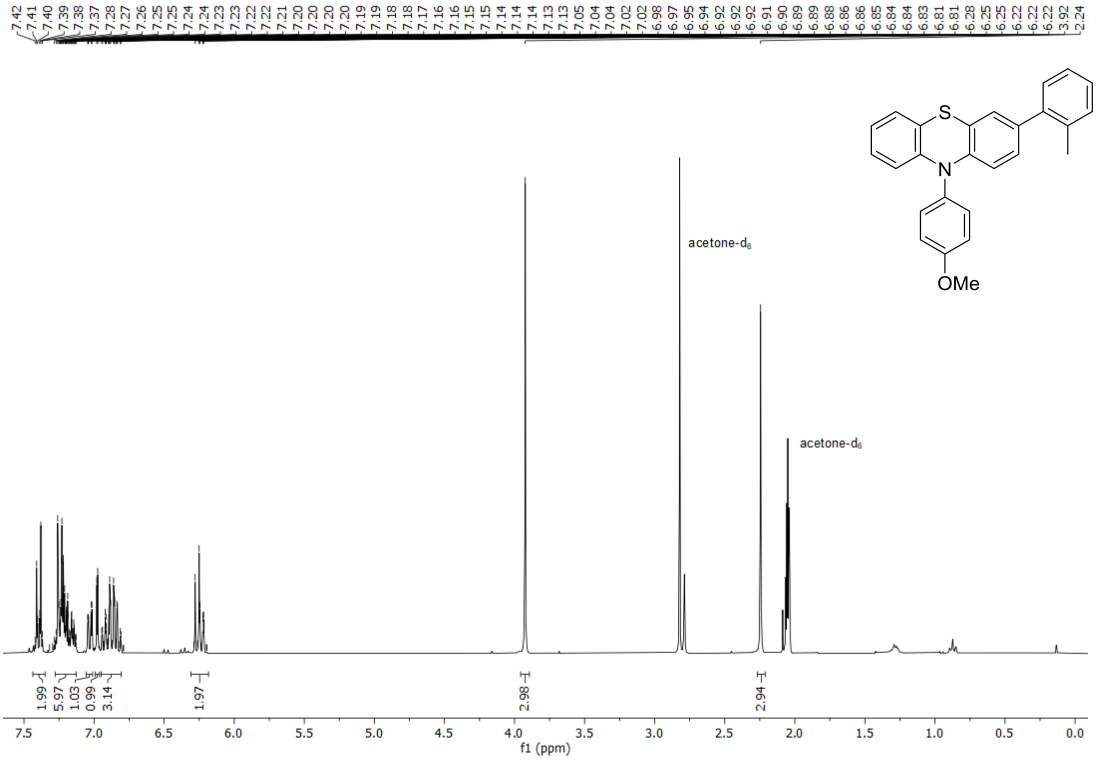

^{13}C NMR spectrum of 10-(4-Methoxyphenyl)-3-(*o*-tolyl)-10*H*-phenothiazine (4n) (acetone-d$_6$, 75 MHz, 293 K)

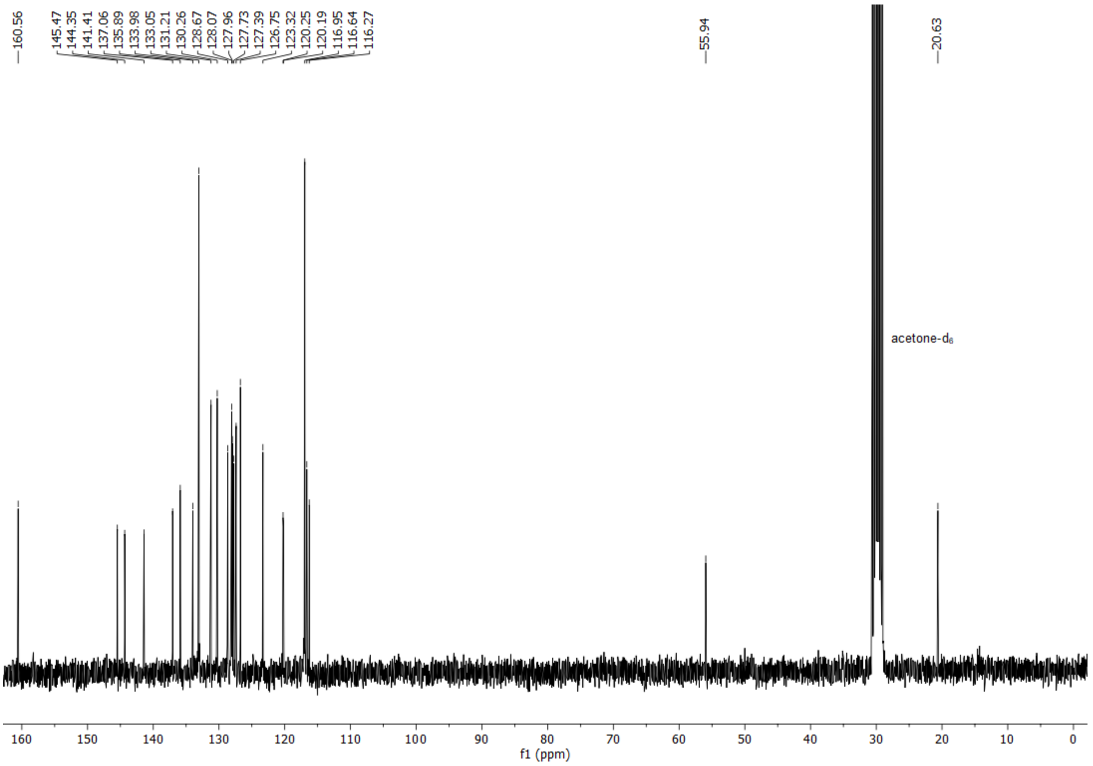

^{1}H NMR spectrum of 2-(10-(4-Methoxyphenyl)-10*H*-phenothiazin-3-yl)benzaldehyde (4o) (acetone-d_6, 600 MHz, 293 K)

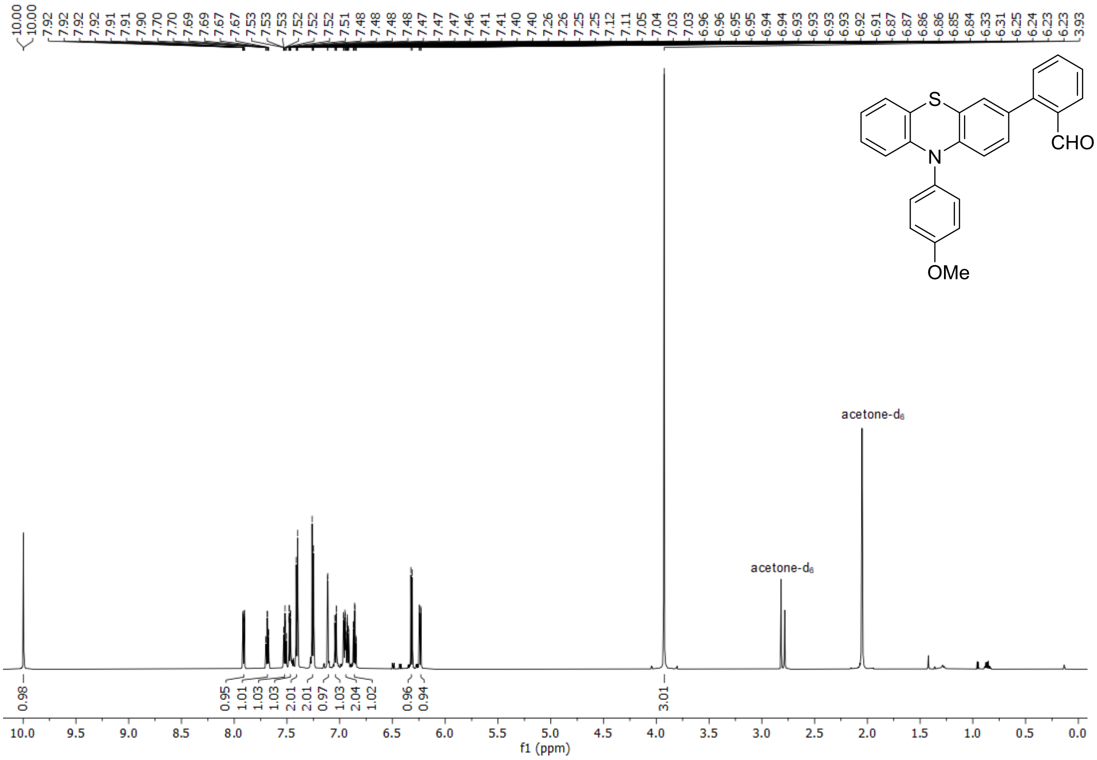

^{13}C NMR spectrum of 2-(10-(4-Methoxyphenyl)-10*H*-phenothiazin-3-yl)benzaldehyde (4o) (acetone-d_6, 75 MHz, 293 K)

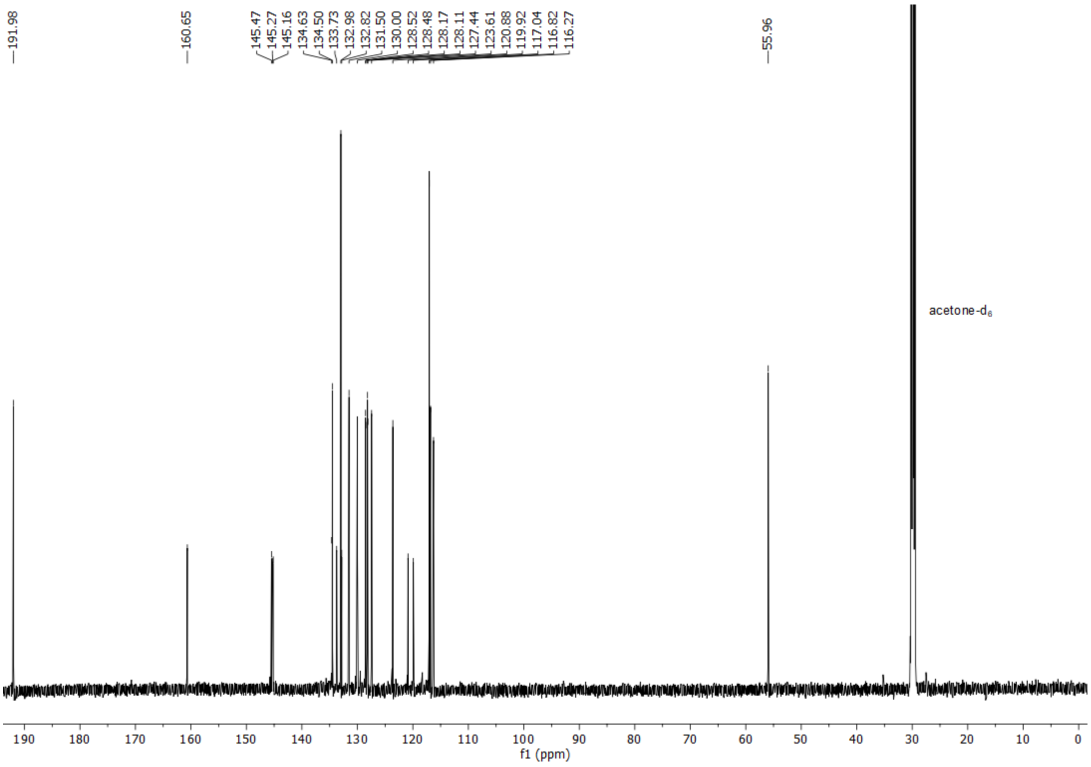

^{1}H NMR spectrum of 3-(4-Methoxyphenyl)-10-(*o*-tolyl)-10*H*-phenothiazine (4p) (acetone-d_6, 600 MHz, 293 K)

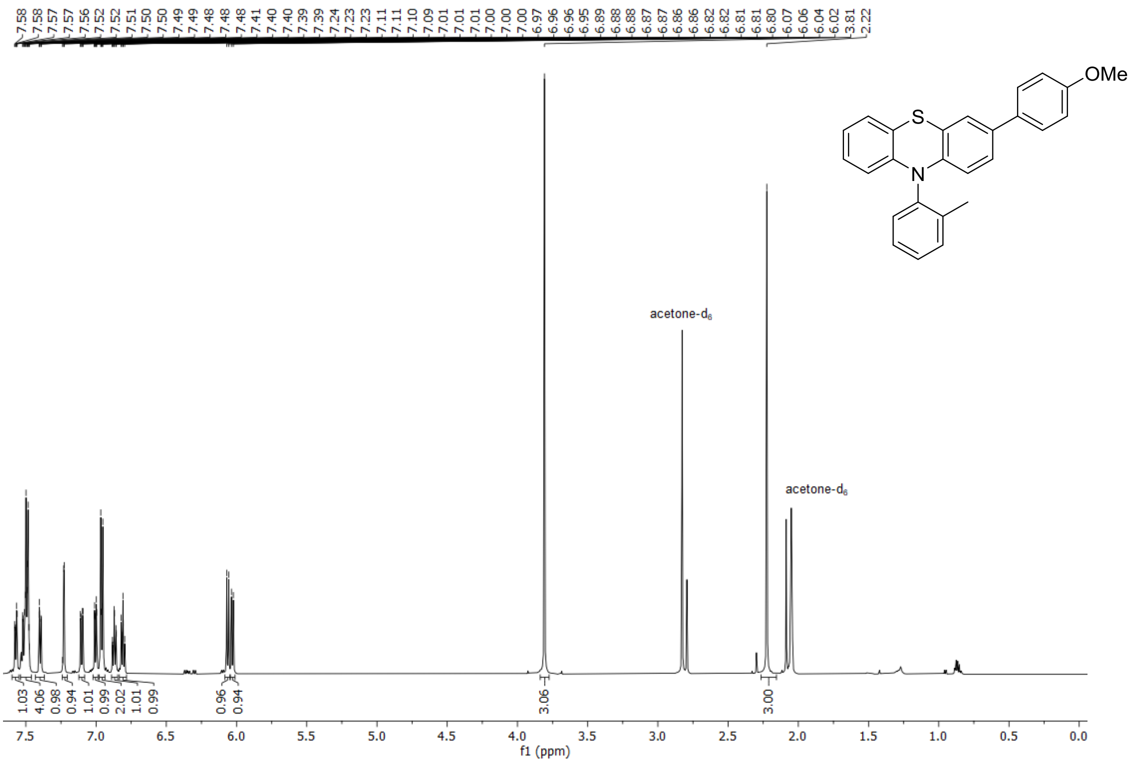

^{13}C NMR spectrum of 3-(4-Methoxyphenyl)-10-(*o*-tolyl)-10*H*-phenothiazine (4p) (acetone-d_6, 150 MHz, 293 K)

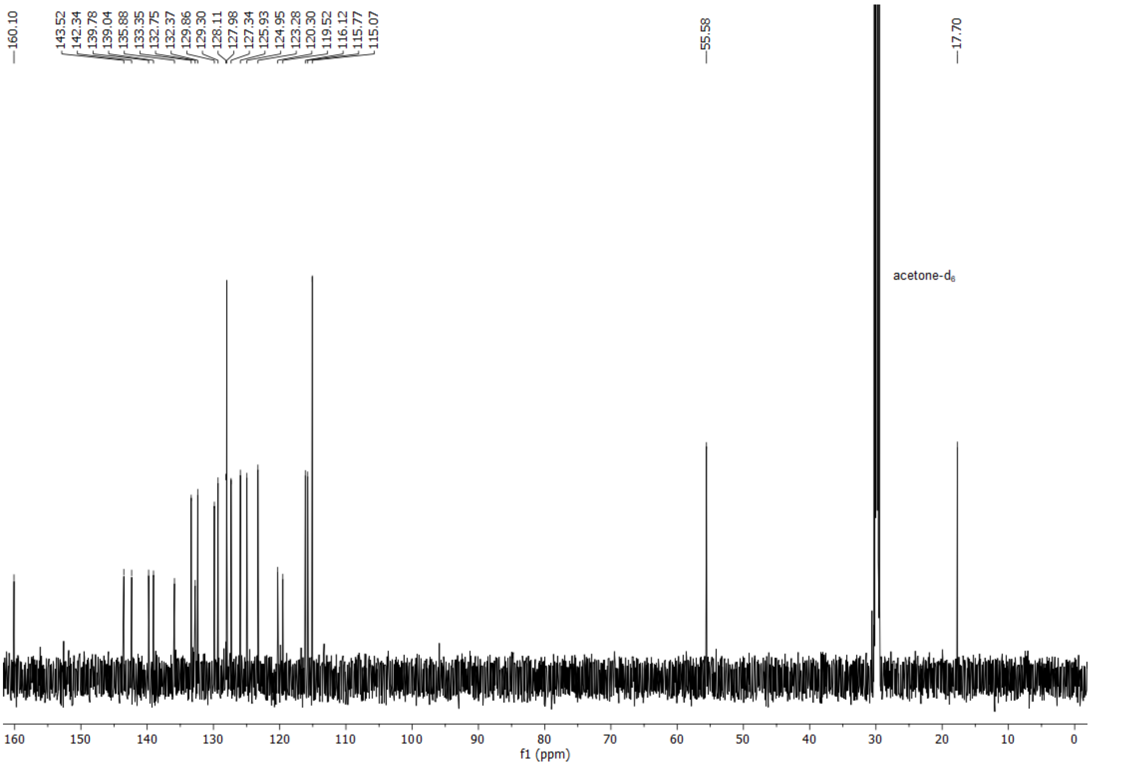

4.2 ^{1}H and ^{13}C NMR spectra of 3,9-diaryl carbazoles 7

^{1}H NMR spectrum of 4,4'-(9*H*-Carbazole-3,9-diyl)bis(*N*,*N*-dimethylaniline) (7a) (acetone-d$_6$, 300 MHz, 293 K)

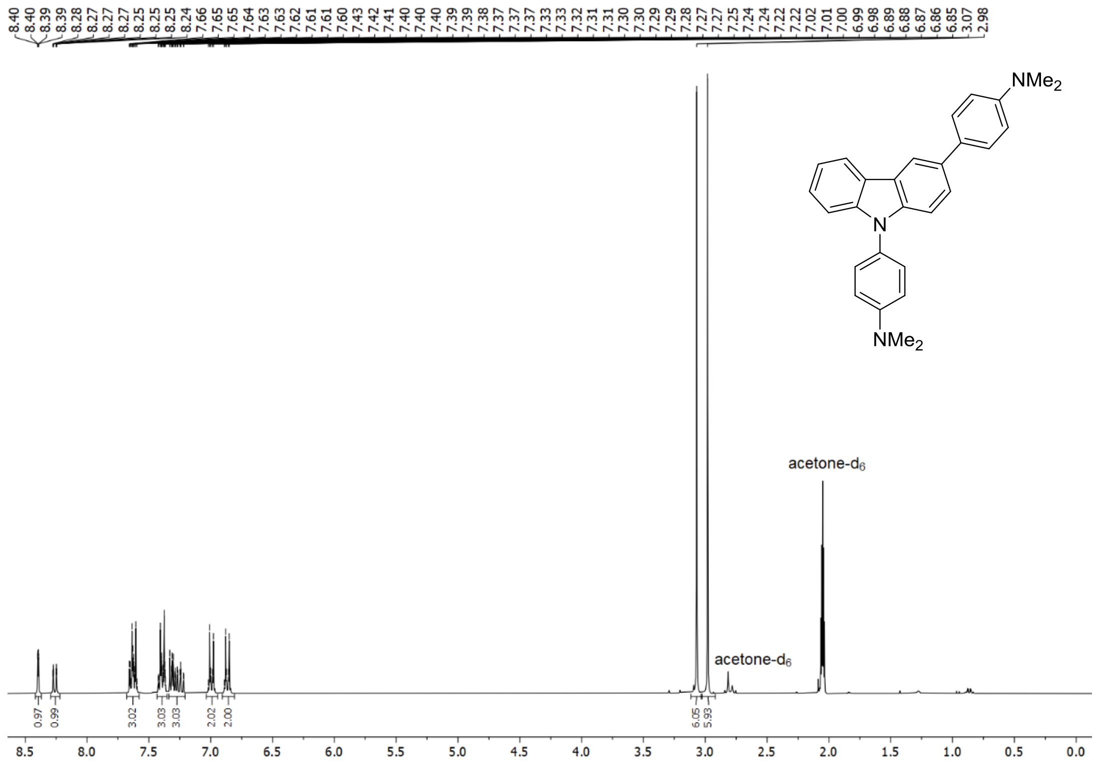

^{13}C NMR spectrum of 4,4'-(9*H*-Carbazole-3,9-diyl)bis(*N*,*N*-dimethylaniline) (7a) (acetone-d$_6$, 75 MHz, 293 K)

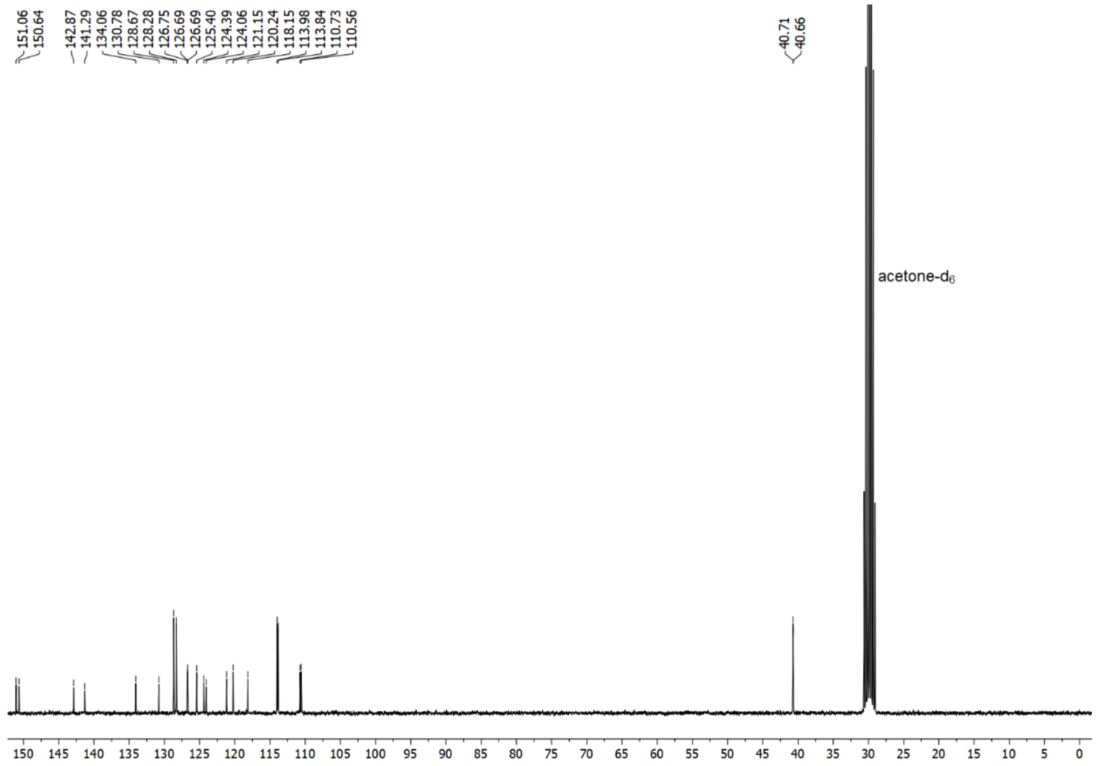

^{1}H NMR spectrum of 4-(3-(4-(Dimethylamino)phenyl)-9*H*-carbazol-9-yl)benzonitrile (7b) (acetone-d$_6$, 300 MHz, 293 K)

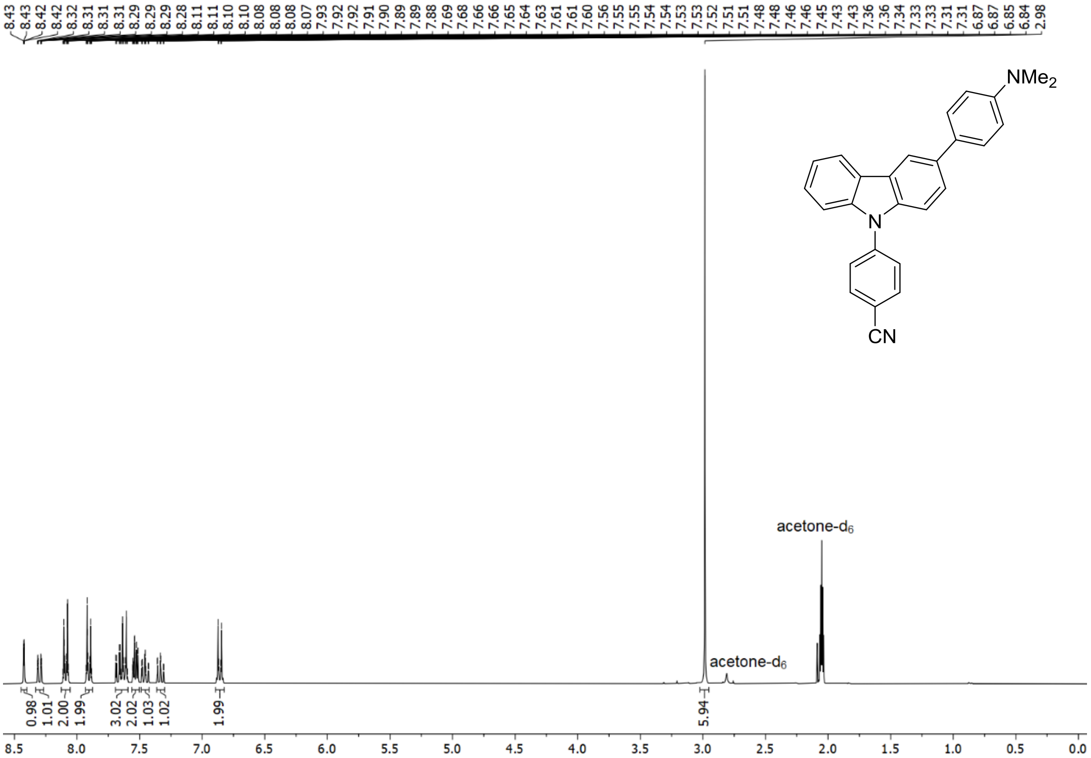

^{13}C NMR spectrum of 4-(3-(4-(Dimethylamino)phenyl)-9*H*-carbazol-9-yl)benzonitrile (7b) (acetone-d$_6$, 75 MHz, 293 K)

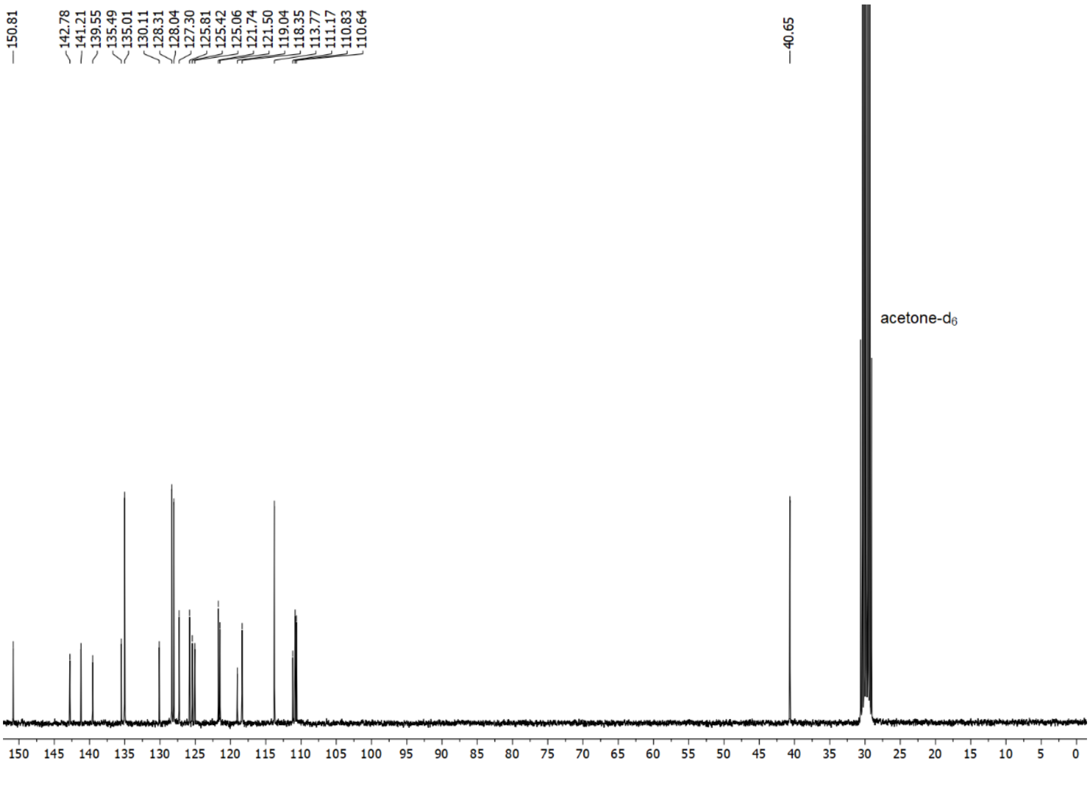

^{1}H NMR spectrum of 3,9-Bis(4-methoxyphenyl)-9*H*-carbazole (7c) (acetone-d$_6$, 600 MHz, 293 K)

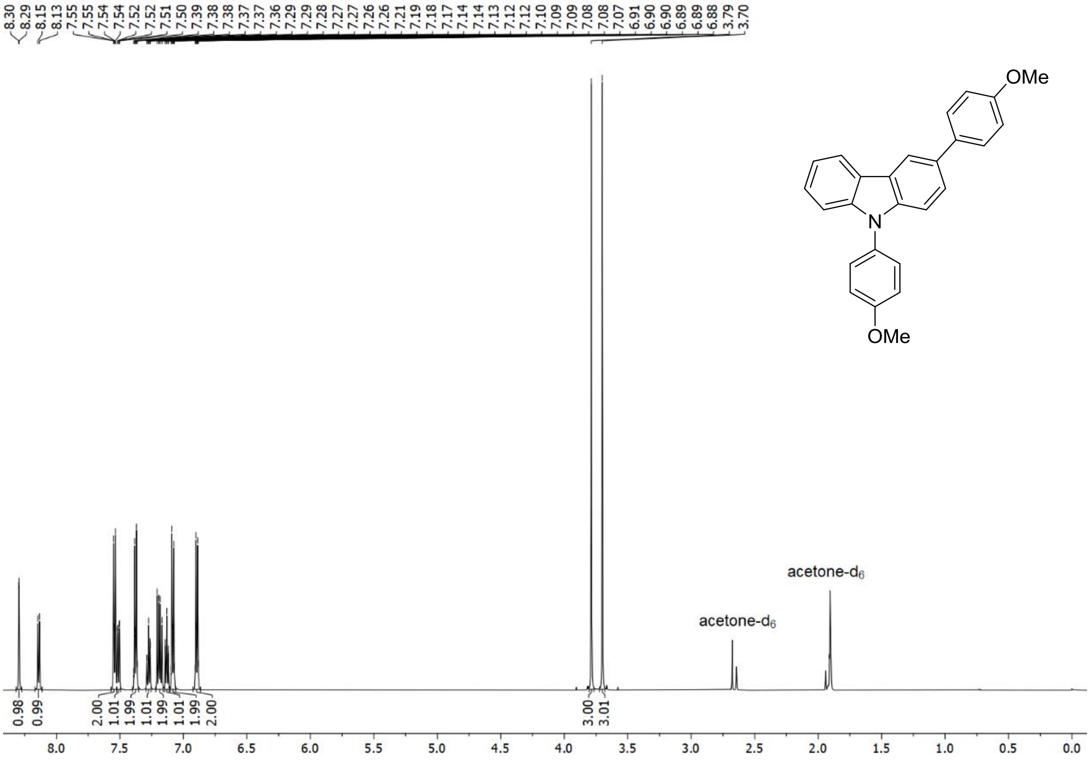

^{13}C NMR spectrum of 3,9-Bis(4-methoxyphenyl)-9*H*-carbazole (7c) (acetone-d$_6$, 150 MHz, 293 K)

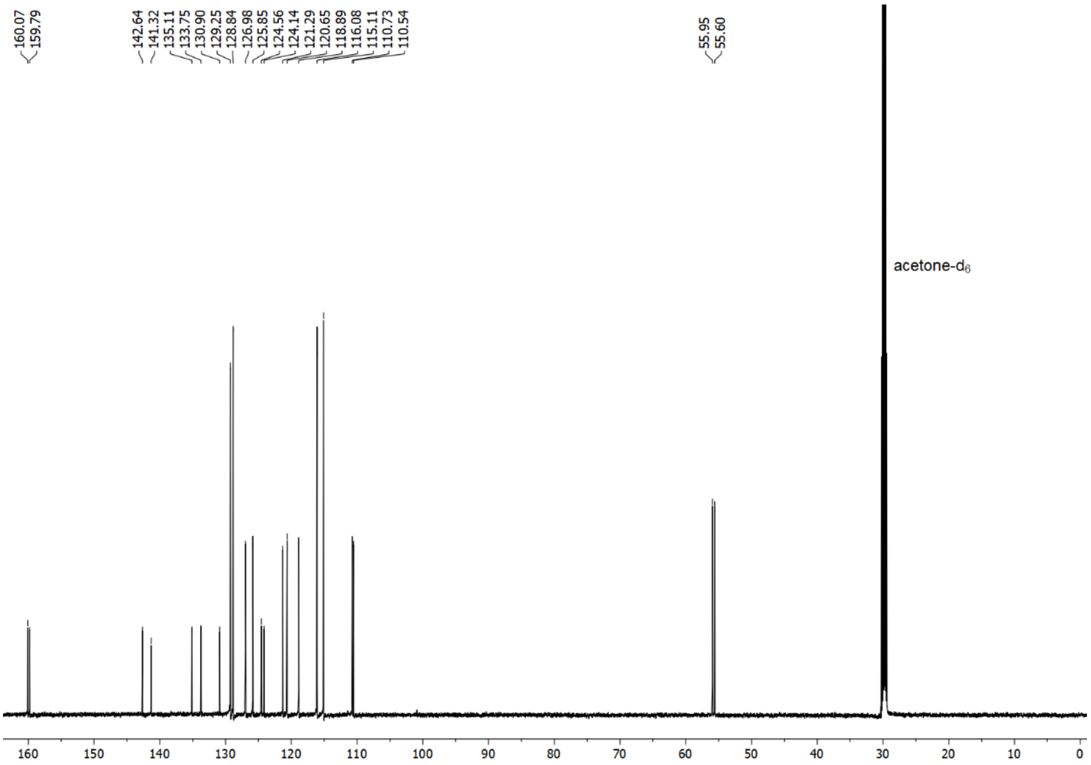

^{1}H NMR spectrum of *N*,*N*-Dimethyl-4-(3-phenyl-9*H*-carbazol-9-yl)aniline (7d) (acetone-d_6, 600 MHz, 293 K)

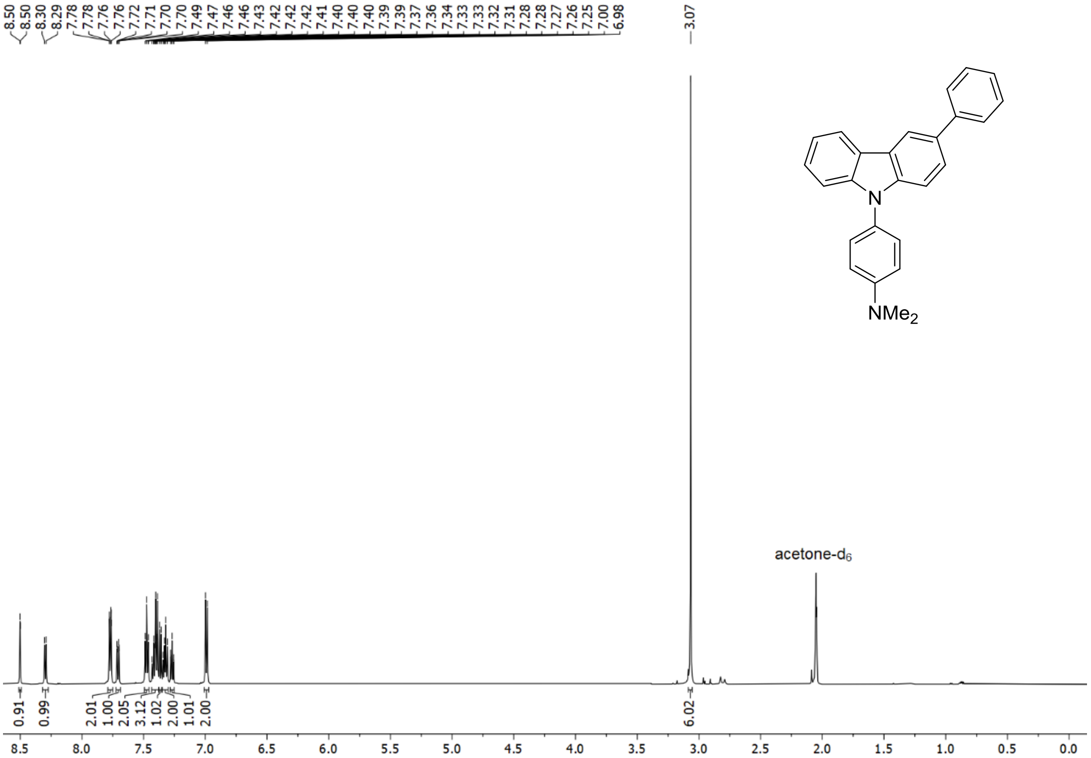

^{13}C NMR spectrum of *N*,*N*-Dimethyl-4-(3-phenyl-9*H*-carbazol-9-yl)aniline (7d) (acetone-d_6, 150 MHz, 293 K)

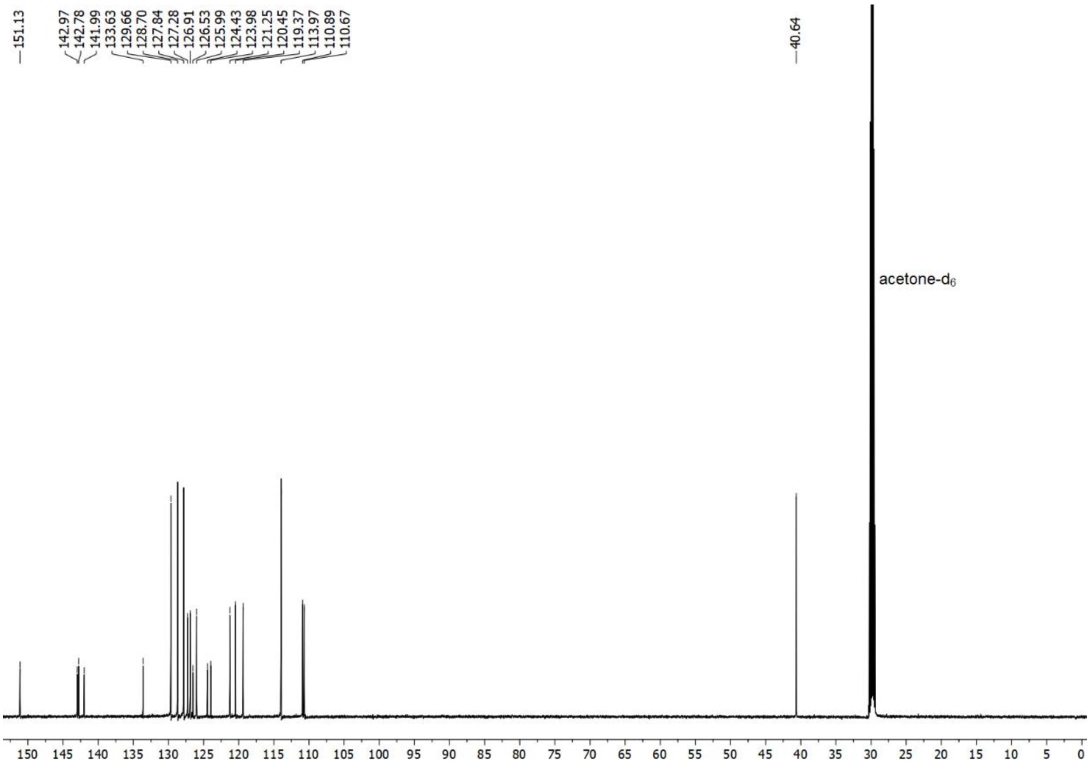

^{1}H NMR spectrum of 3,9-Diphenyl-9*H*-carbazole (7e) (acetone-d_6, 600 MHz, 293 K)

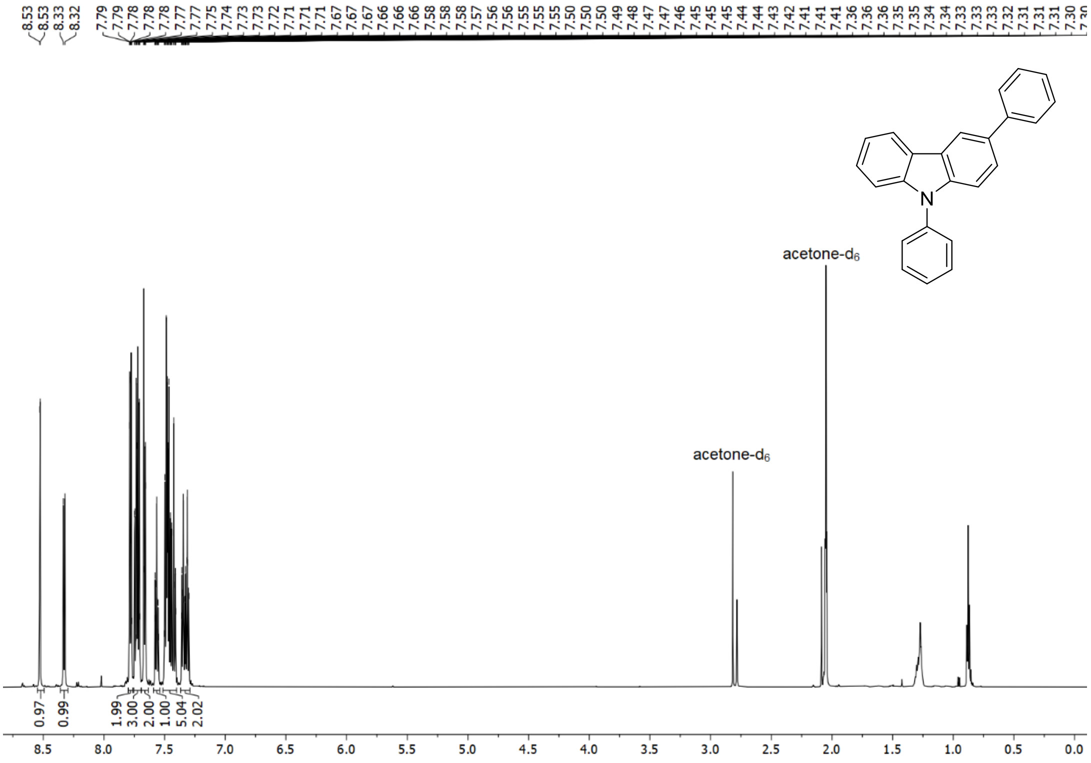

^{13}C NMR spectrum of 3,9-Diphenyl-9*H*-carbazole (7e) (acetone-d_6, 150 MHz, 293 K)

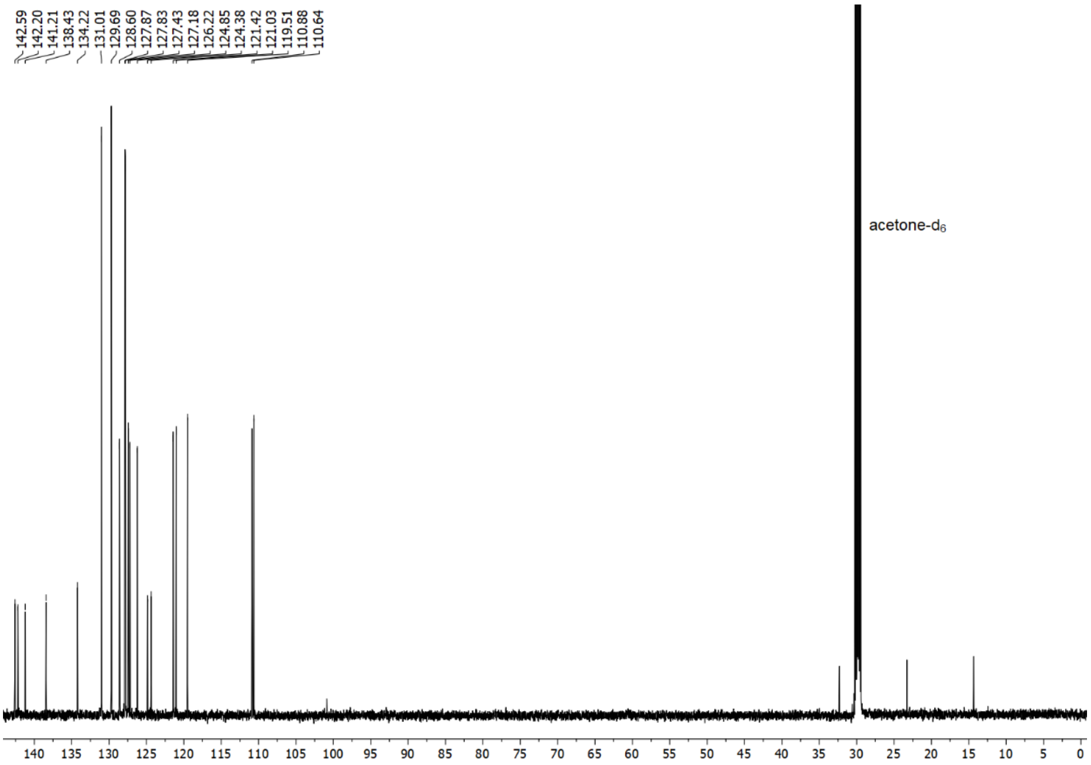

^{1}H NMR spectrum of 4-(3-Phenyl-9*H*-carbazol-9-yl)benzonitrile (7f) (acetone-d_6, 600 MHz, 293 K)

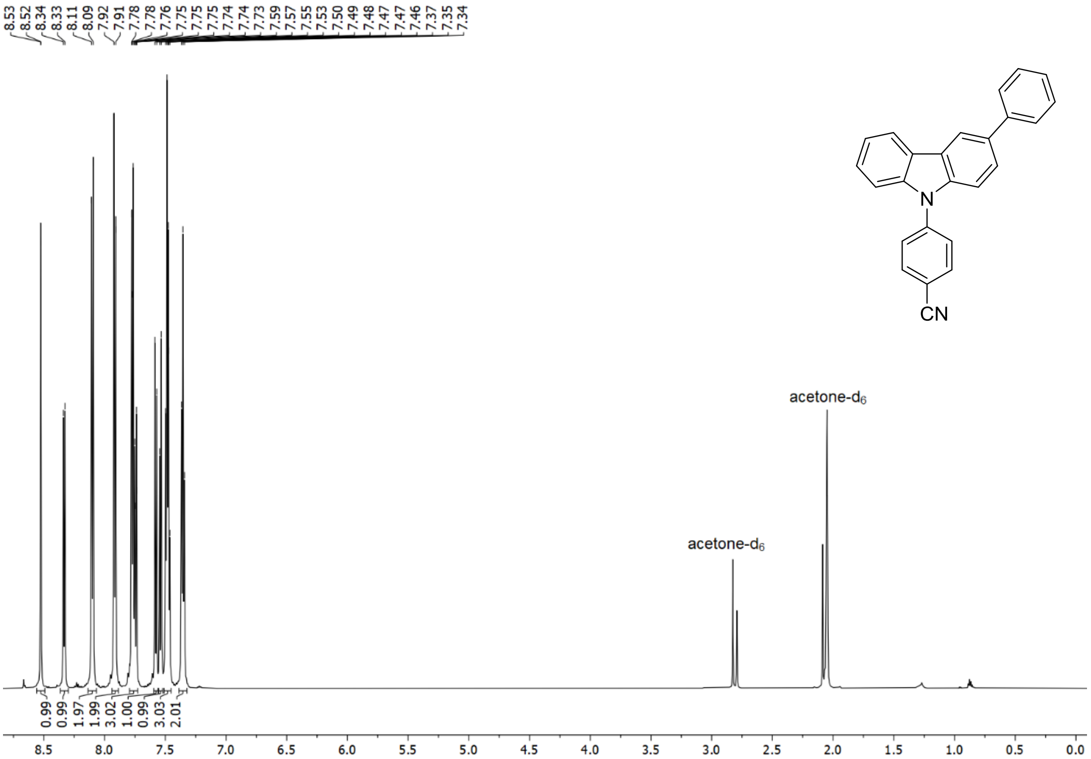

^{13}C NMR spectrum of 4-(3-Phenyl-9*H*-carbazol-9-yl)benzonitrile (7f) (acetone-d_6, 150 MHz, 293 K)

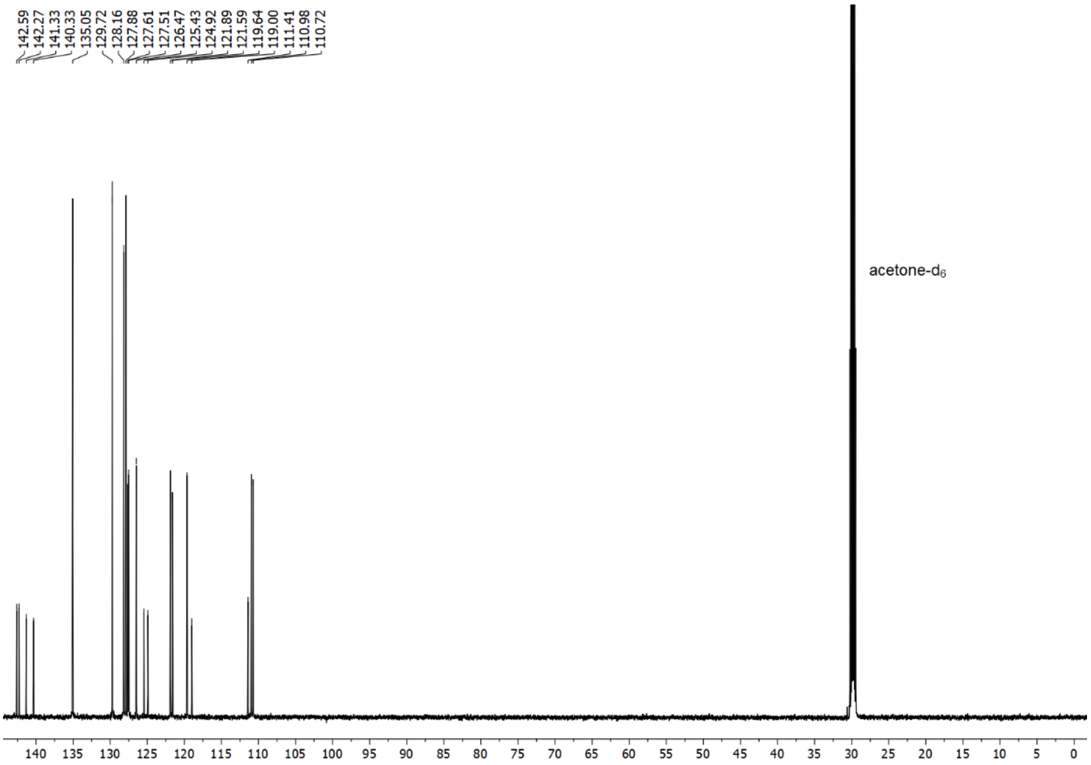

4.3 ^{1}H and ^{13}C NMR spectra of 1,5-diaryl indoles 8

^{1}H NMR spectrum of 1,5-Bis(4-methoxyphenyl)-1*H*-indole (8a) (acetone-d_6, 600 MHz, 293 K)

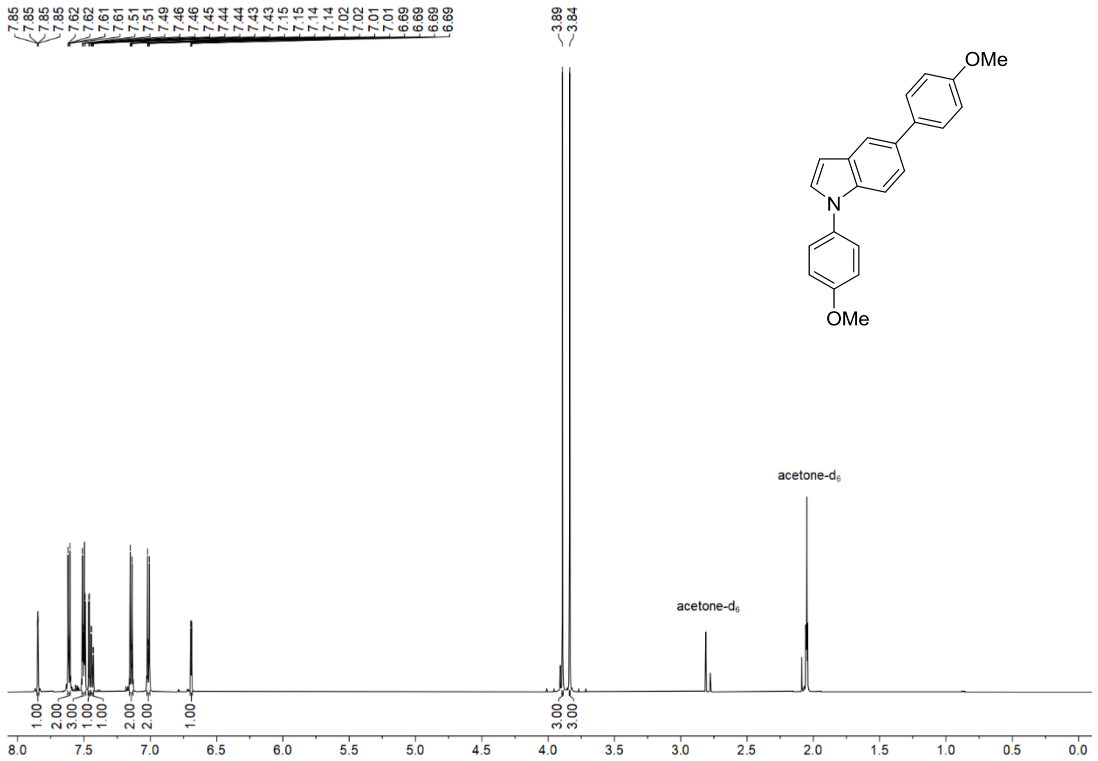

^{13}C NMR spectrum of 1,5-Bis(4-methoxyphenyl)-1*H*-indole (8a) (acetone-d_6, 75 MHz, 293 K)

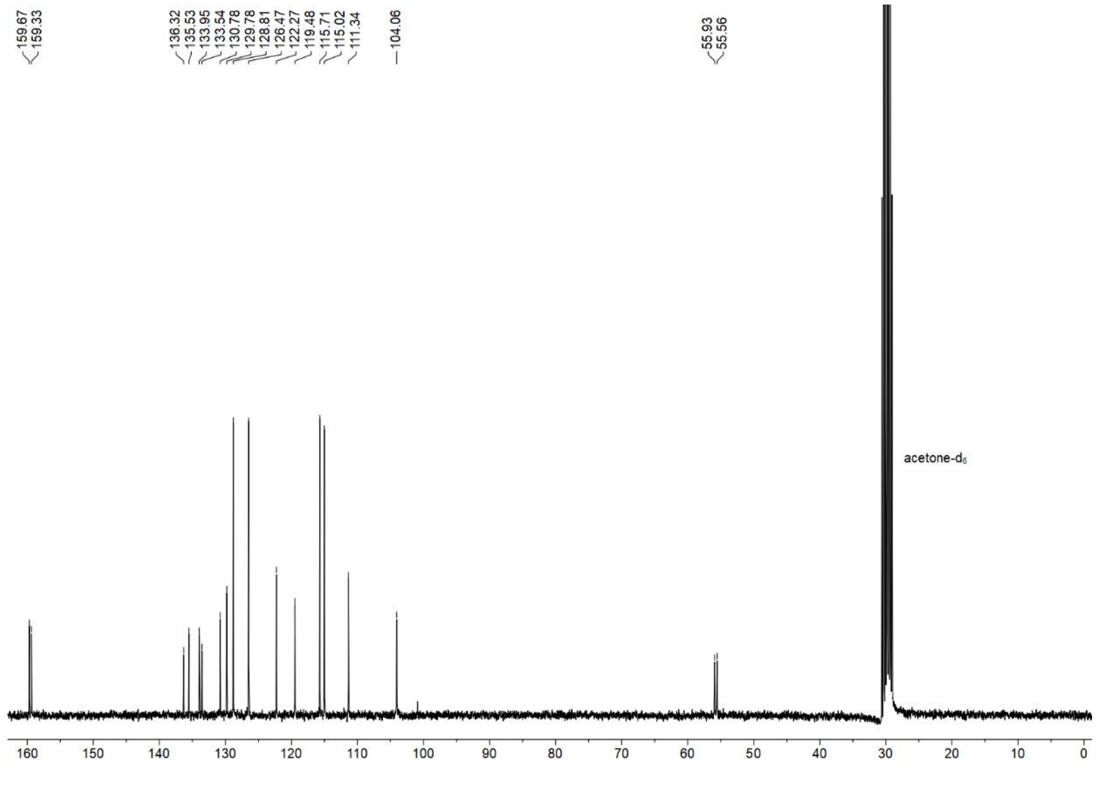

^{1}H NMR spectrum of 5-(4-Methoxyphenyl)-1-phenyl-1*H*-indole (8b) (acetone-d_6, 600 MHz, 293 K)

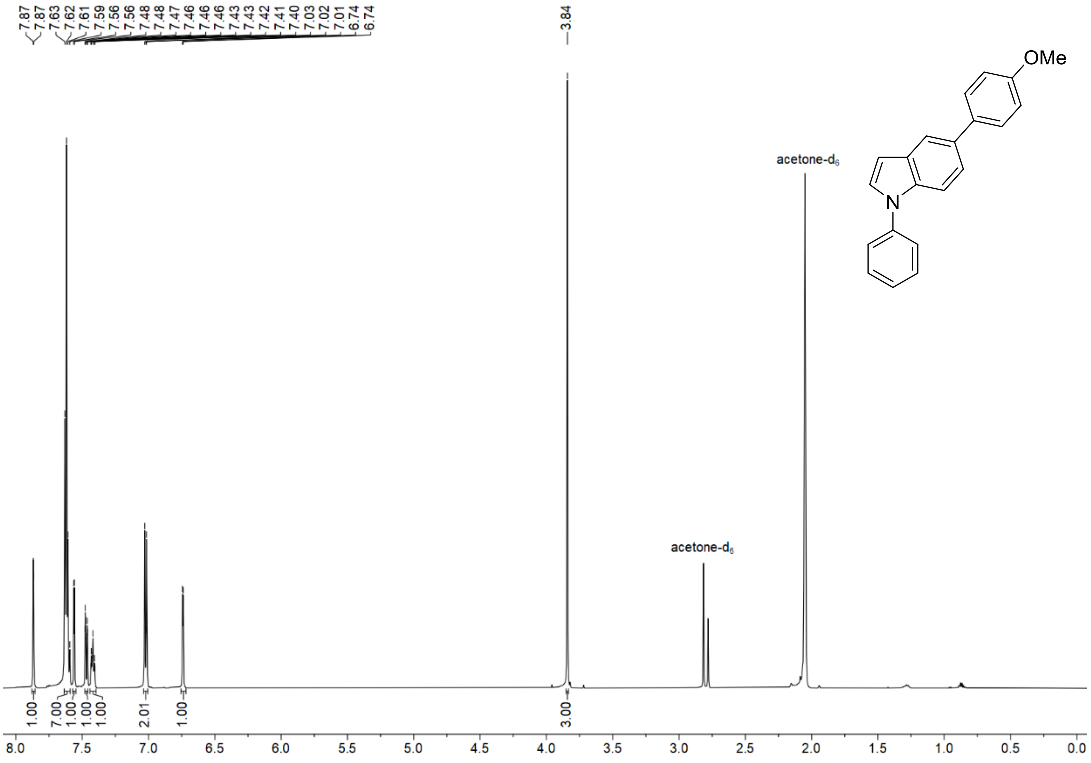

^{13}C NMR spectrum of 5-(4-Methoxyphenyl)-1-phenyl-1*H*-indole (8b) (acetone-d_6, 150 MHz, 293 K)

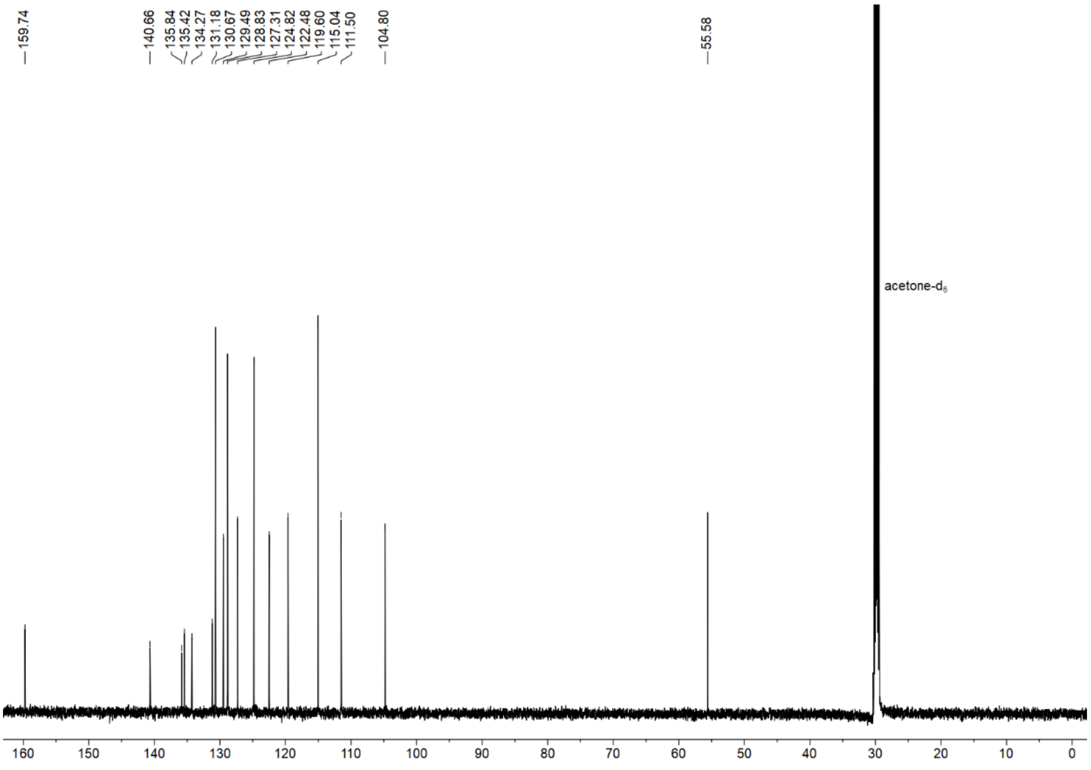

^{1}H NMR spectrum of 1-(4-Chlorophenyl)-5-(4-methoxyphenyl)-1*H*-indole (8c) (acetone-d_6, 300 MHz, 293 K)

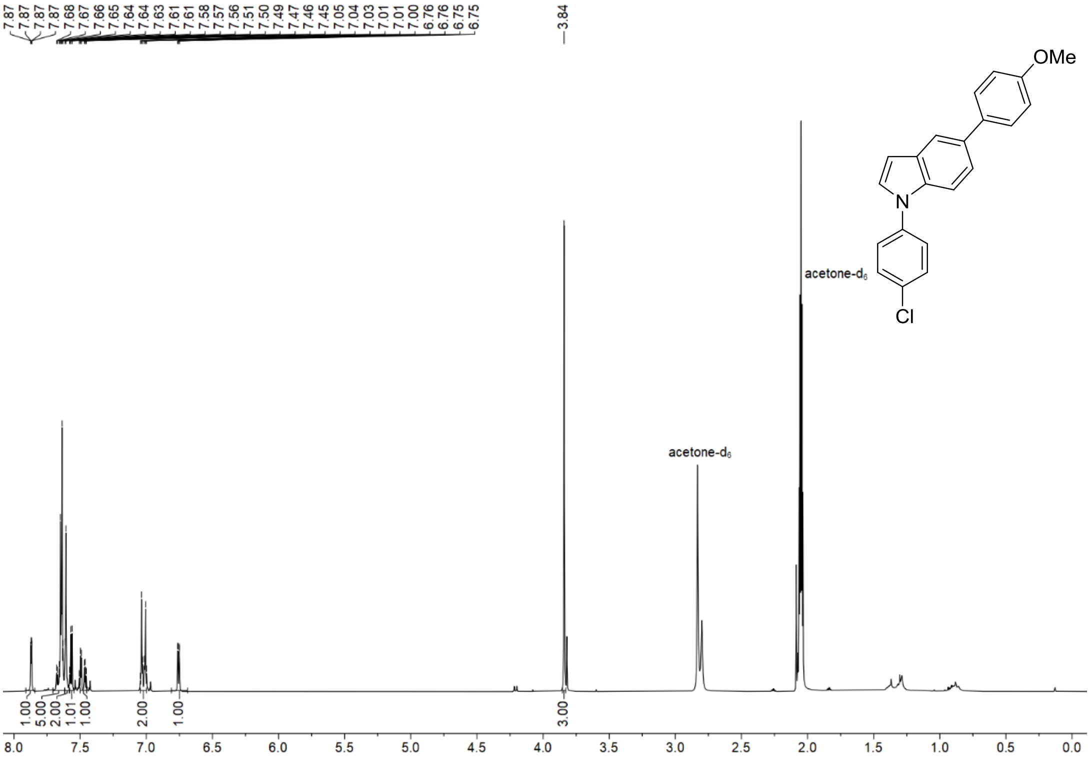

^{13}C NMR spectrum of 1-(4-Chlorophenyl)-5-(4-methoxyphenyl)-1*H*-indole (8c) (acetone-d_6, 75 MHz, 293 K)

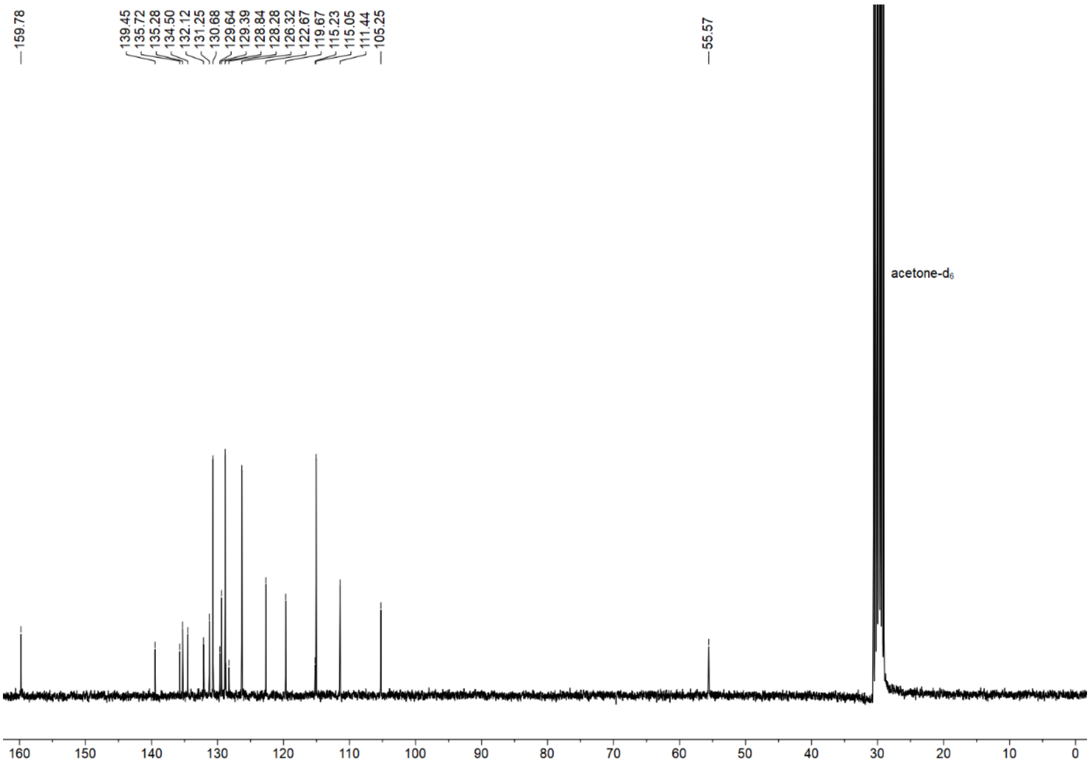

^{1}H NMR spectrum of 5-(4-Methoxyphenyl)-1-(4-(trifluoromethyl)phenyl)-1*H*-indole (8d) (acetone-d_6, 300 MHz, 293 K)

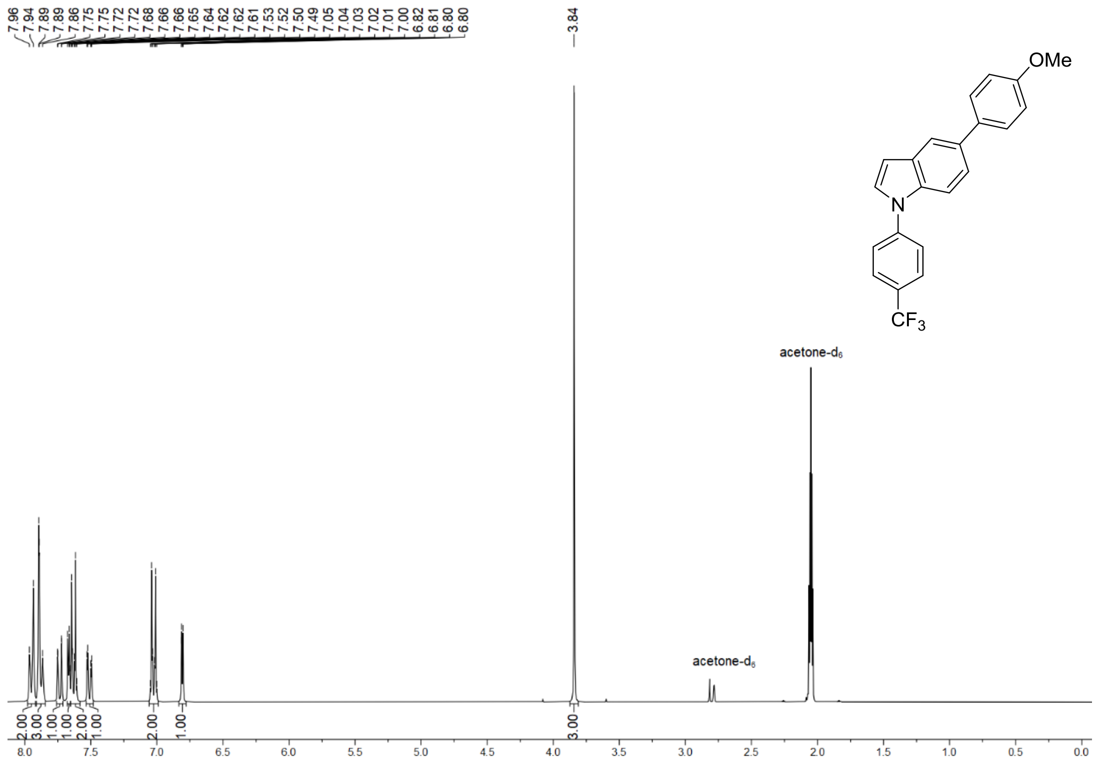

^{13}C NMR spectrum of 5-(4-Methoxyphenyl)-1-(4-(trifluoromethyl)phenyl)-1*H*-indole (8d) (acetone-d_6, 75 MHz, 293 K)

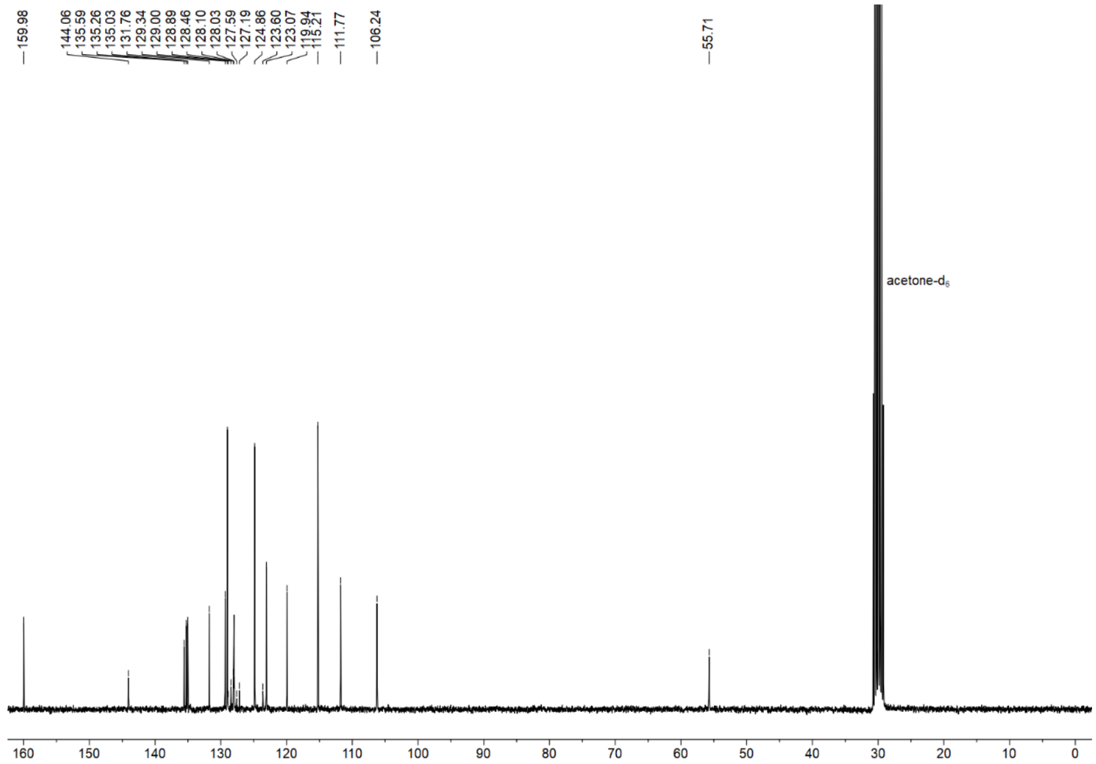

^{1}H NMR spectrum of 1-(4-Methoxyphenyl)-5-phenyl-1*H*-indole (8e) (acetone-d$_6$, 300 MHz, 293 K)

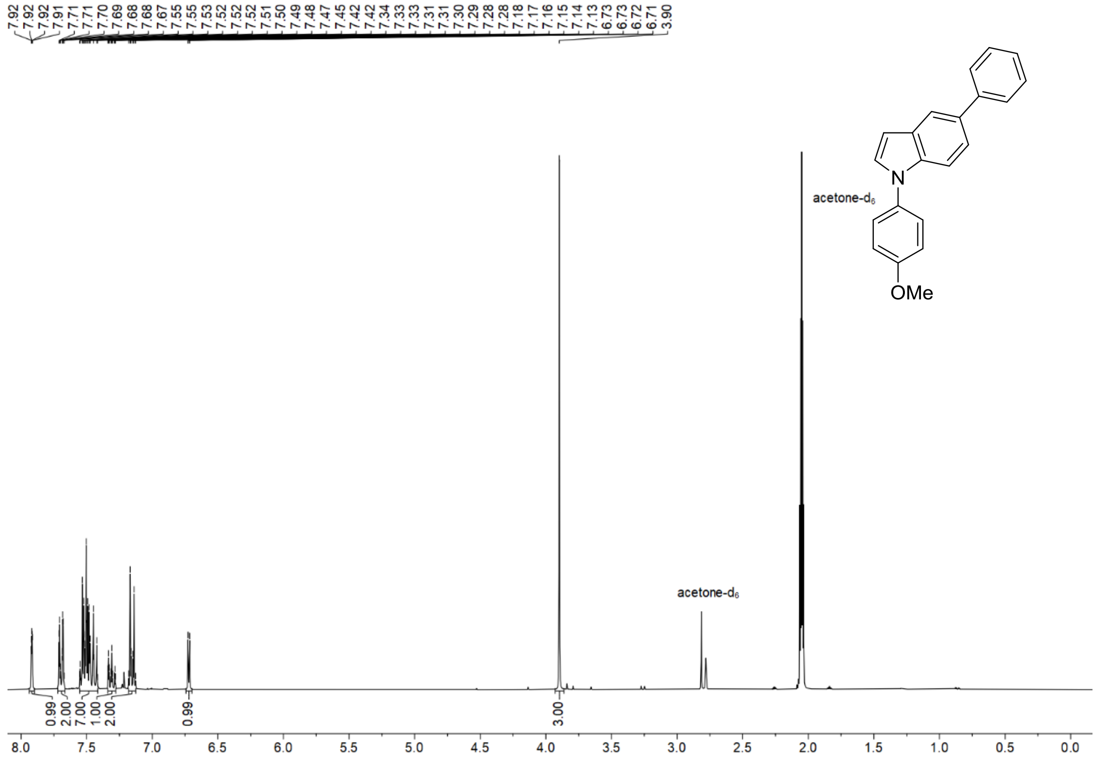

^{13}C NMR spectrum of 1-(4-Methoxyphenyl)-5-phenyl-1*H*-indole (8e) (acetone-d$_6$, 75 MHz, 293 K)

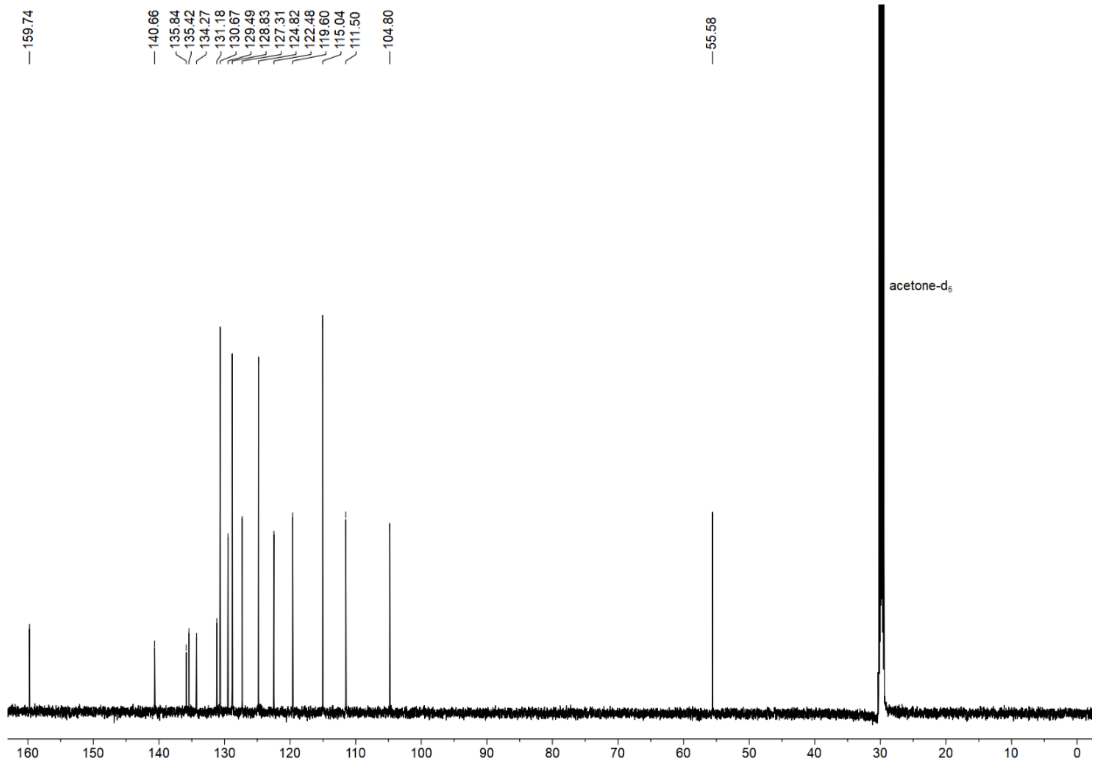

^{1}H NMR spectrum of 1,5-Diphenyl-1*H*-indole (8f) (acetone-d_6, 300 MHz, 293 K)

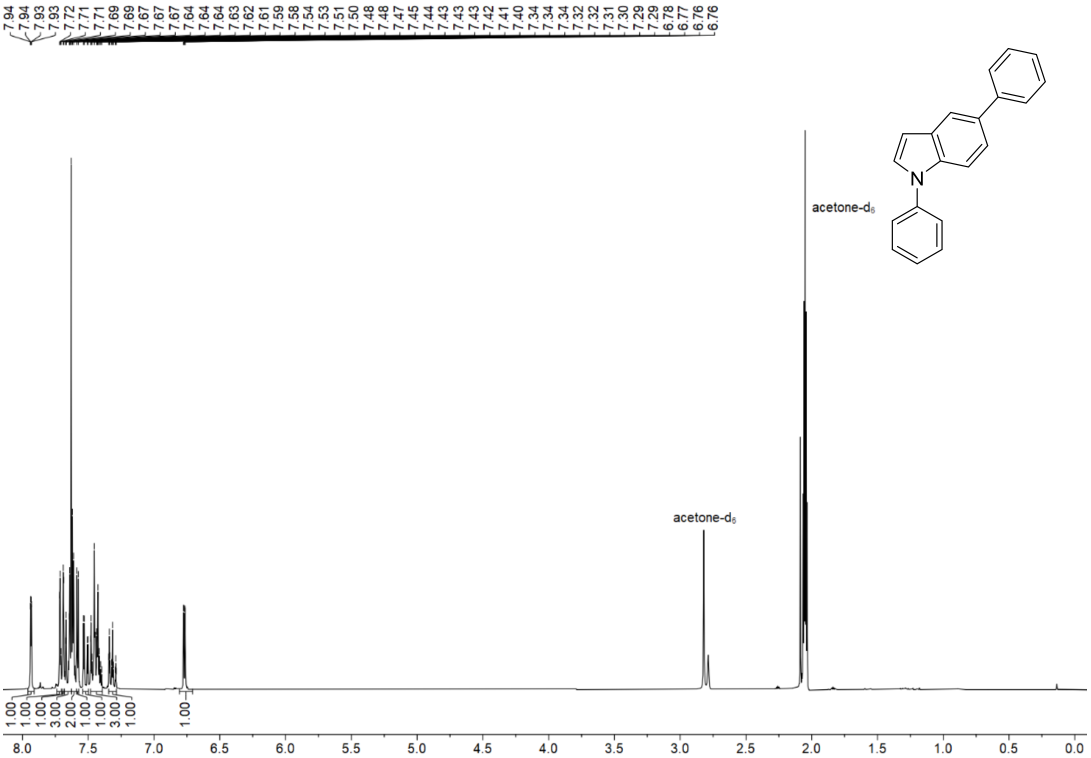

^{13}C NMR spectrum of 1,5-Diphenyl-1*H*-indole (8f) (acetone-d_6, 75 MHz, 293 K)

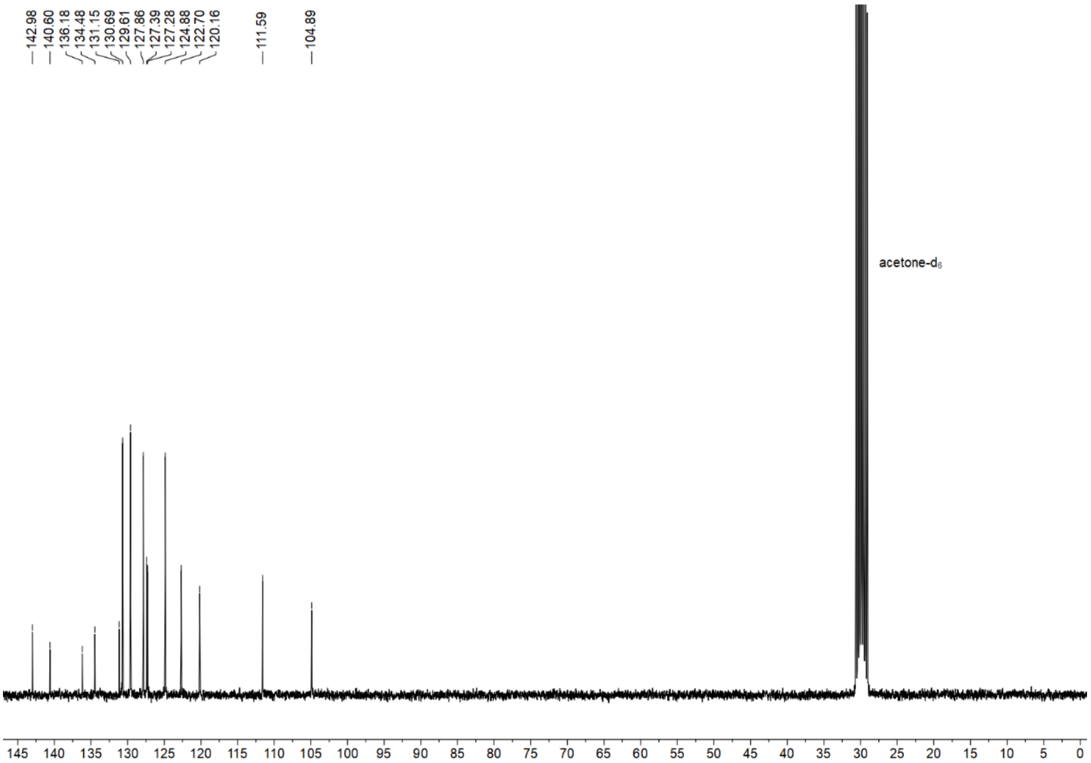

^{1}H NMR spectrum of 5-Phenyl-1-(4-(trifluoromethyl)phenyl)-1*H*-indole (8g) (acetone-d$_6$, 600 MHz, 293 K)

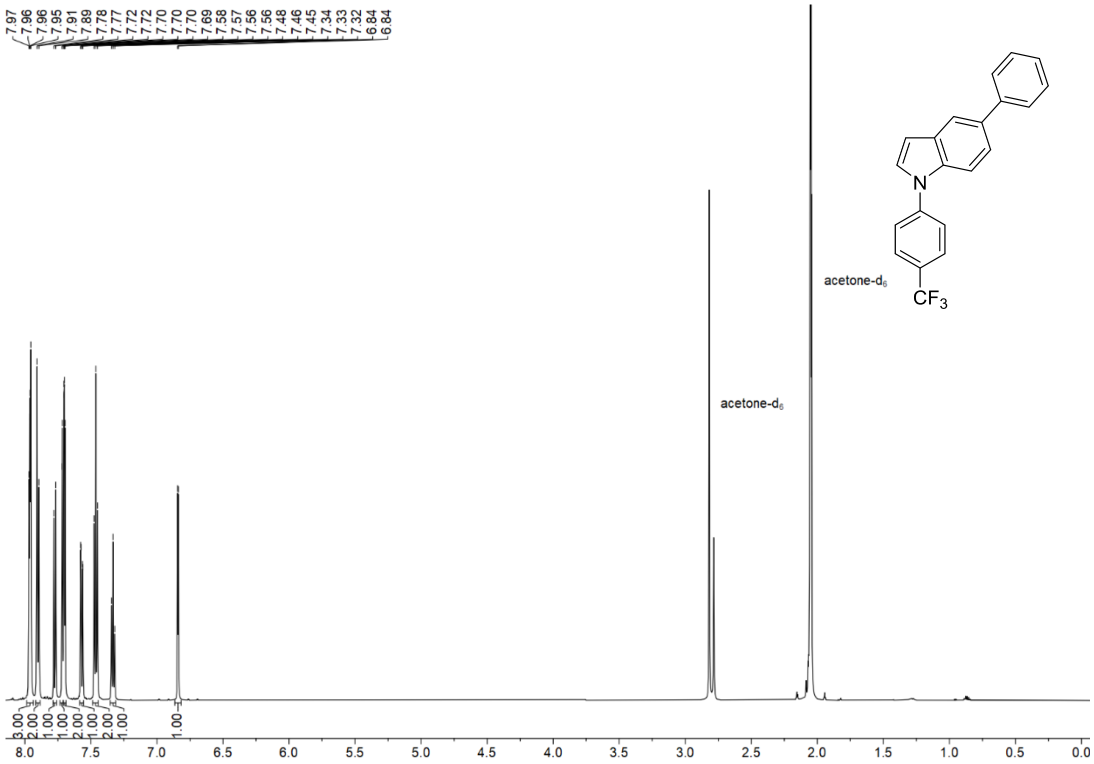

^{13}C NMR spectrum of 5-Phenyl-1-(4-(trifluoromethyl)phenyl)-1*H*-indole (8g) (acetone-d$_6$, 150 MHz, 293 K)

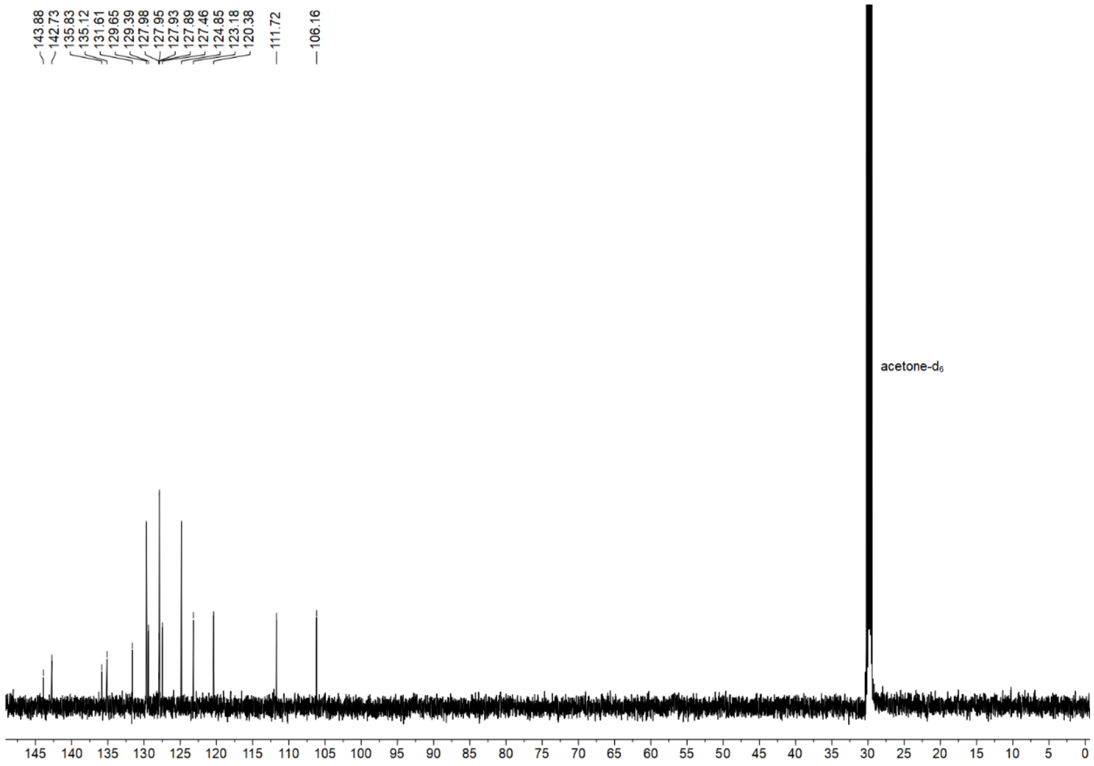

^{1}H NMR spectrum of 1-(4-Methoxyphenyl)-5-(4-(trifluoromethyl)phenyl)-1*H*-indole (8h) (acetone-d_6, 600 MHz, 293 K)

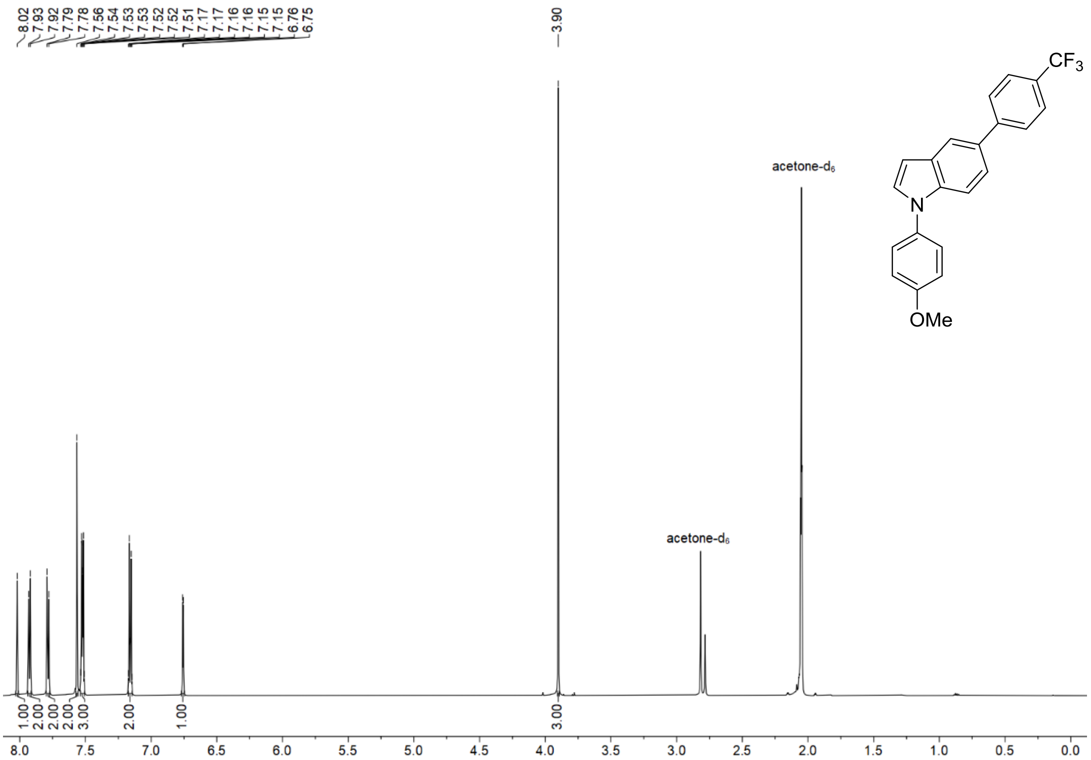

^{13}C NMR spectrum of 1-(4-Methoxyphenyl)-5-(4-(trifluoromethyl)phenyl)-1*H*-indole (8h) (acetone-d_6, 150 MHz, 293 K)

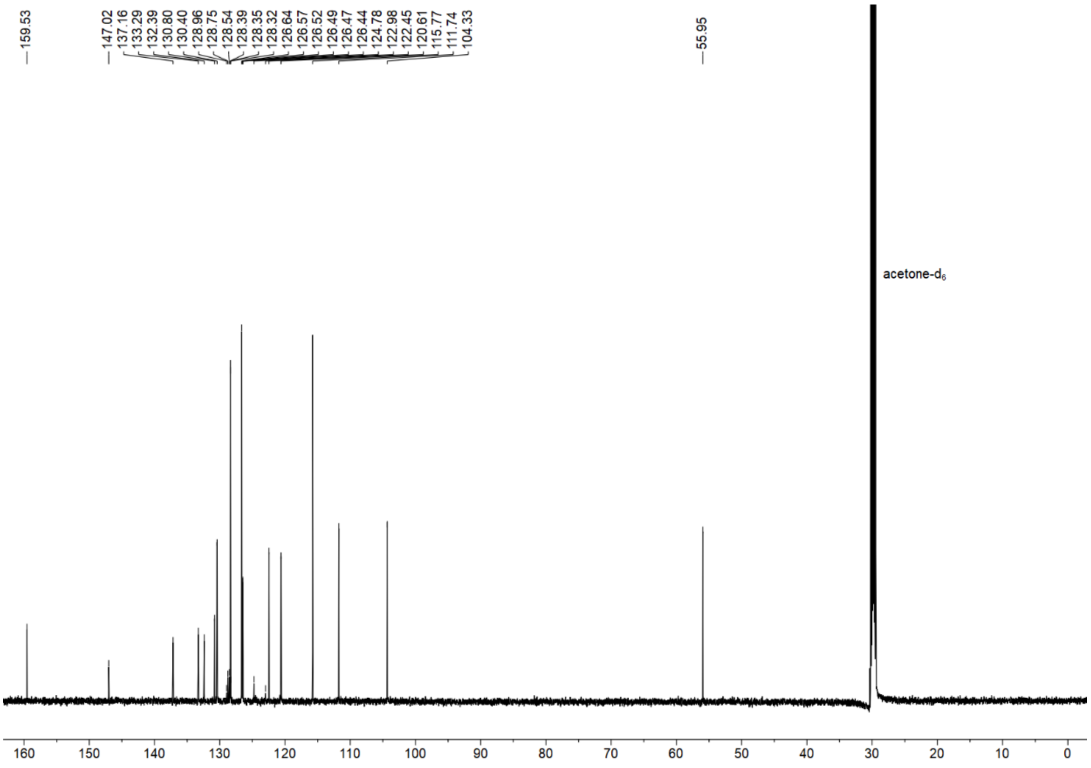

^{1}H NMR spectrum of 1-Phenyl-5-(4-(trifluoromethyl)phenyl)-1*H*-indole (8i) (acetone-d_6, 600 MHz, 293 K)

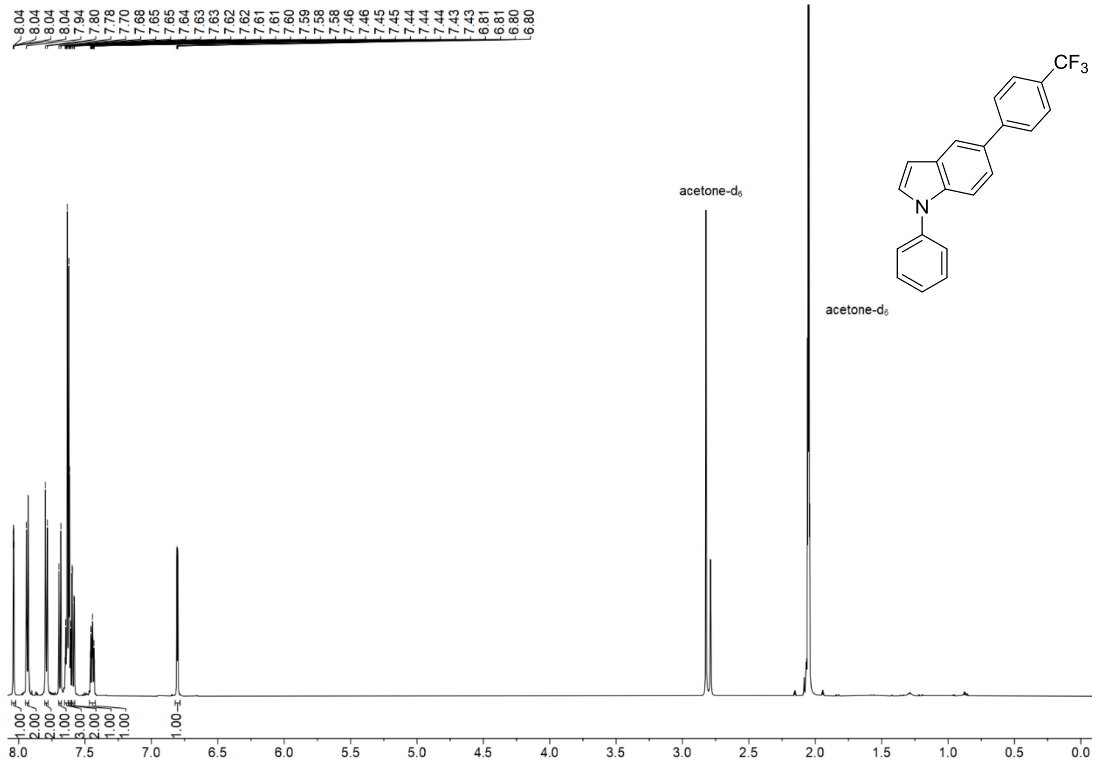

^{13}C NMR spectrum of 1-Phenyl-5-(4-(trifluoromethyl)phenyl)-1*H*-indole (8i) (acetone-d_6, 150 MHz, 293 K)

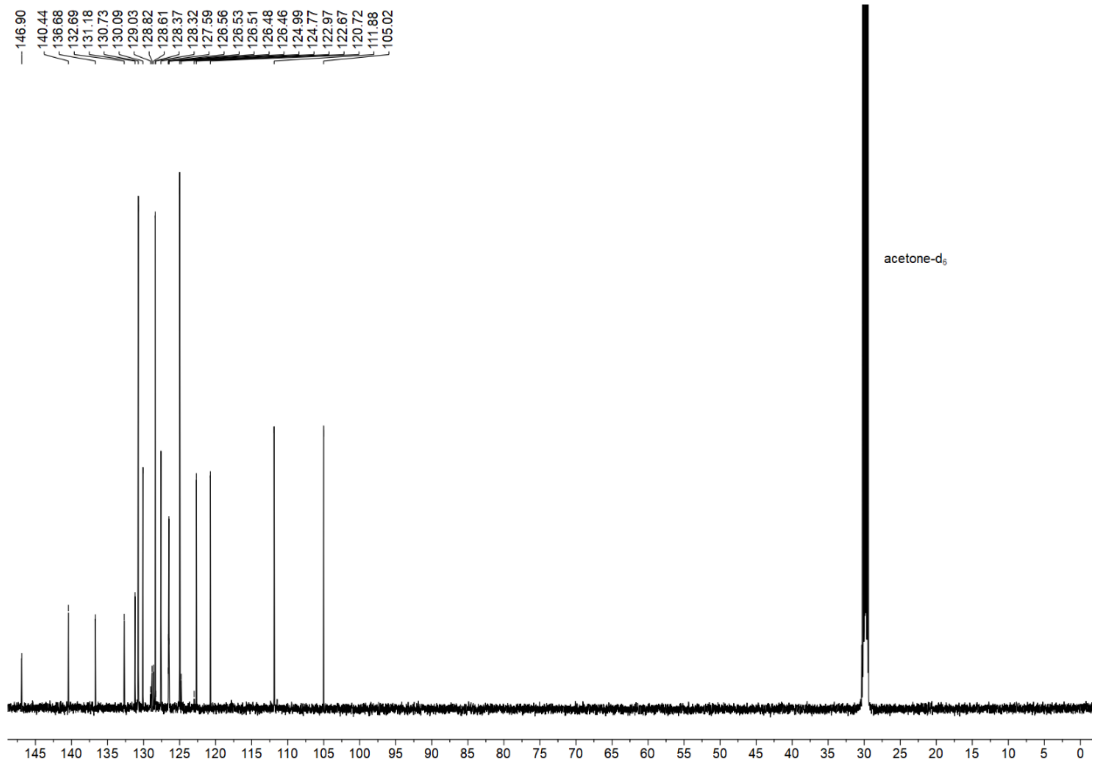

^{1}H NMR spectrum of 1,5-Bis(4-(trifluoromethyl)phenyl)-1*H*-indole (8j) (acetone-d_6, 300 MHz, 293 K)

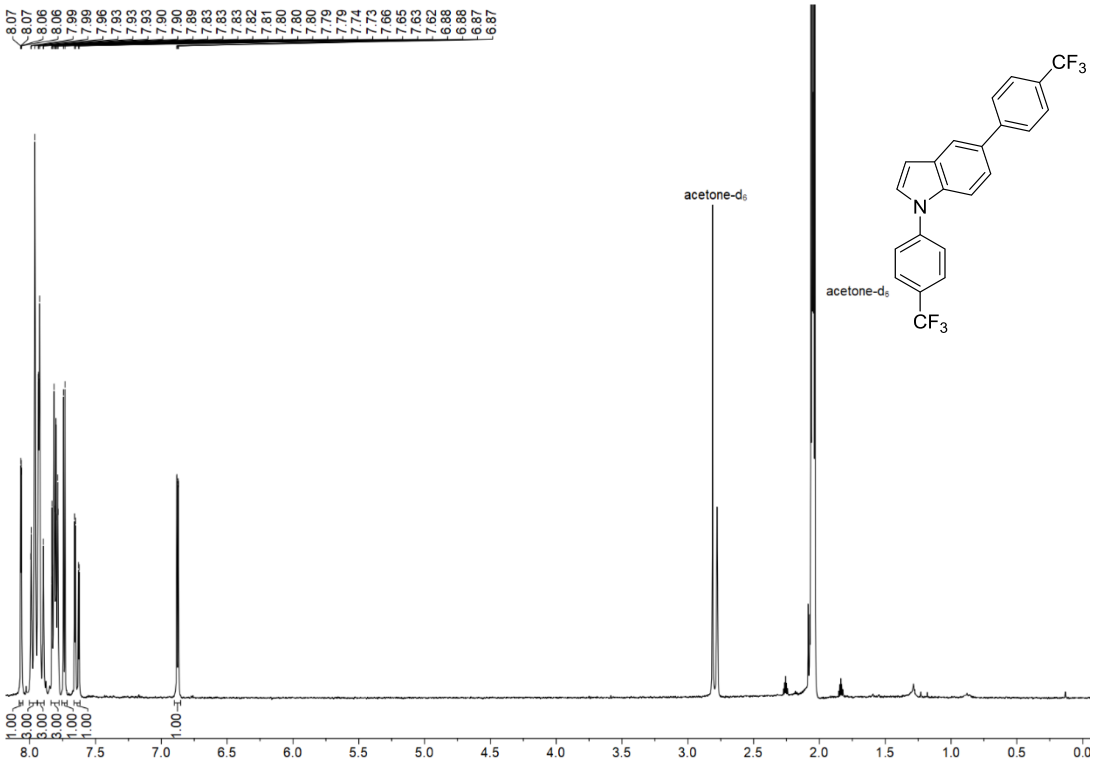

^{13}C NMR spectrum of 1,5-Bis(4-(trifluoromethyl)phenyl)-1*H*-indole (8j) (acetone-d_6, 150 MHz, 293 K)

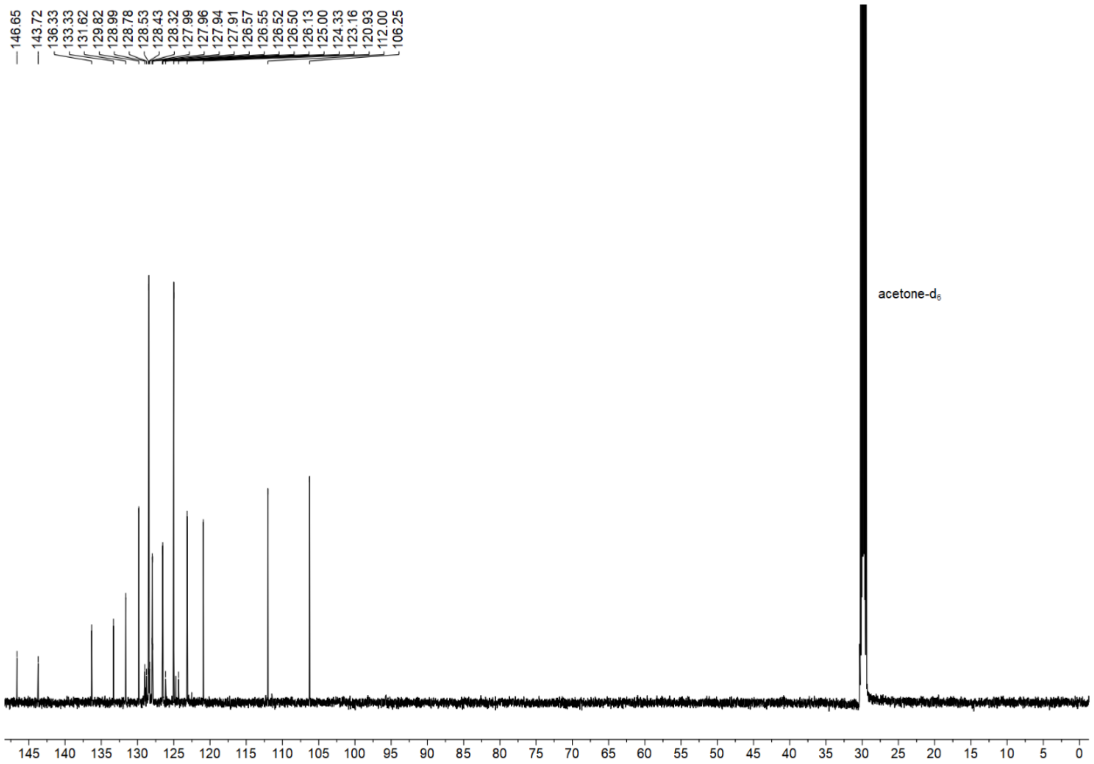

^{1}H NMR spectrum of 1-(4-Methoxyphenyl)-5-(thiophen-2-yl)-1*H*-indole (8k) (acetone-d_6, 600 MHz, 293 K)

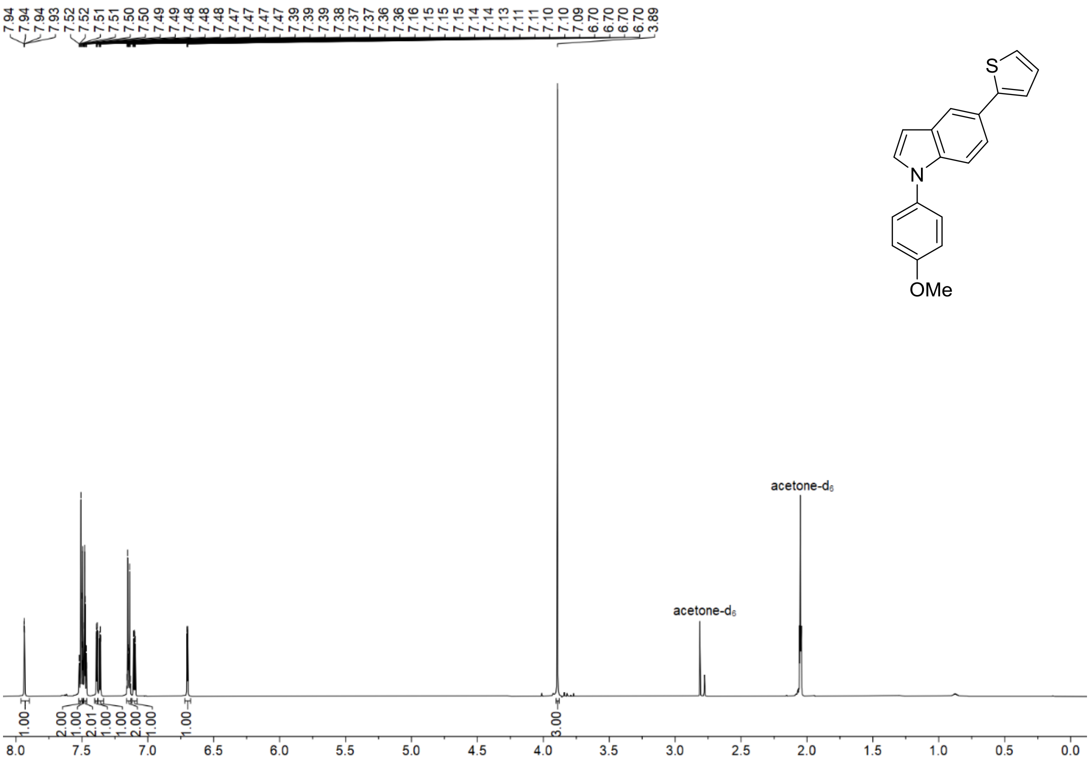

^{13}C NMR spectrum of 1-(4-Methoxyphenyl)-5-(thiophen-2-yl)-1*H*-indole (8k) (acetone-d_6, 150 MHz, 293 K)

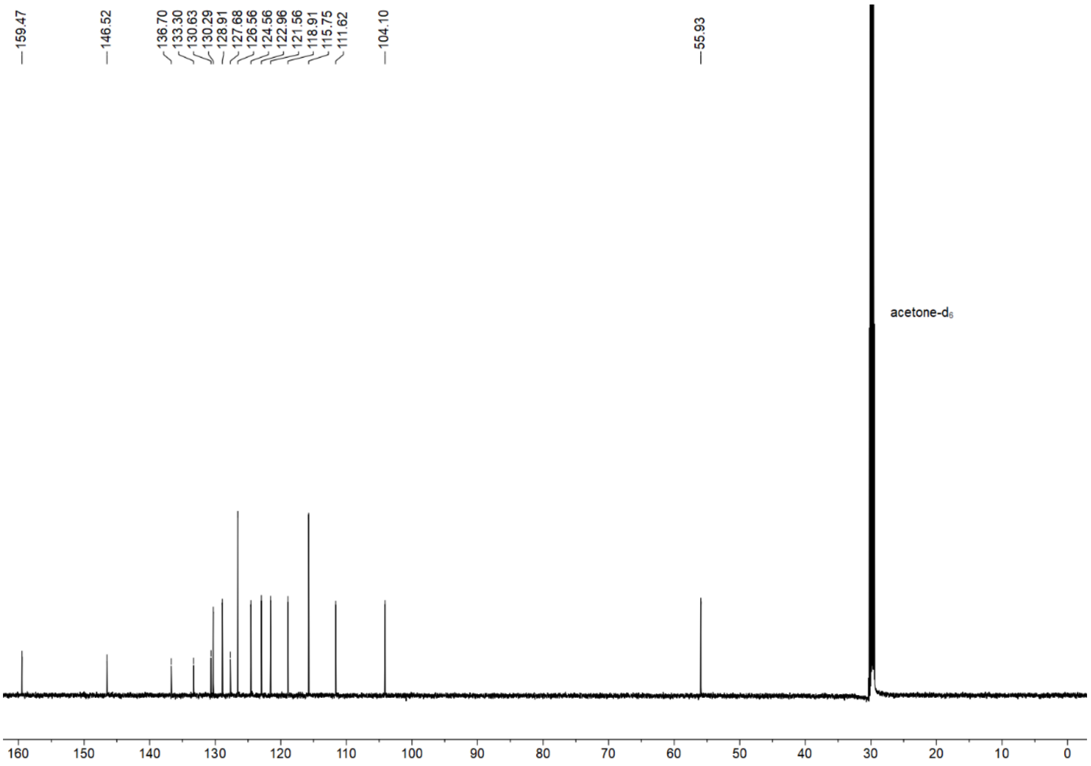

^{1}H NMR spectrum of 10-Hexyl-3-(5-(4-methoxyphenyl)-1*H*-indol-1-yl)-10*H*-phenothiazine (8l) (acetone-d_6, 600 MHz, 293 K)

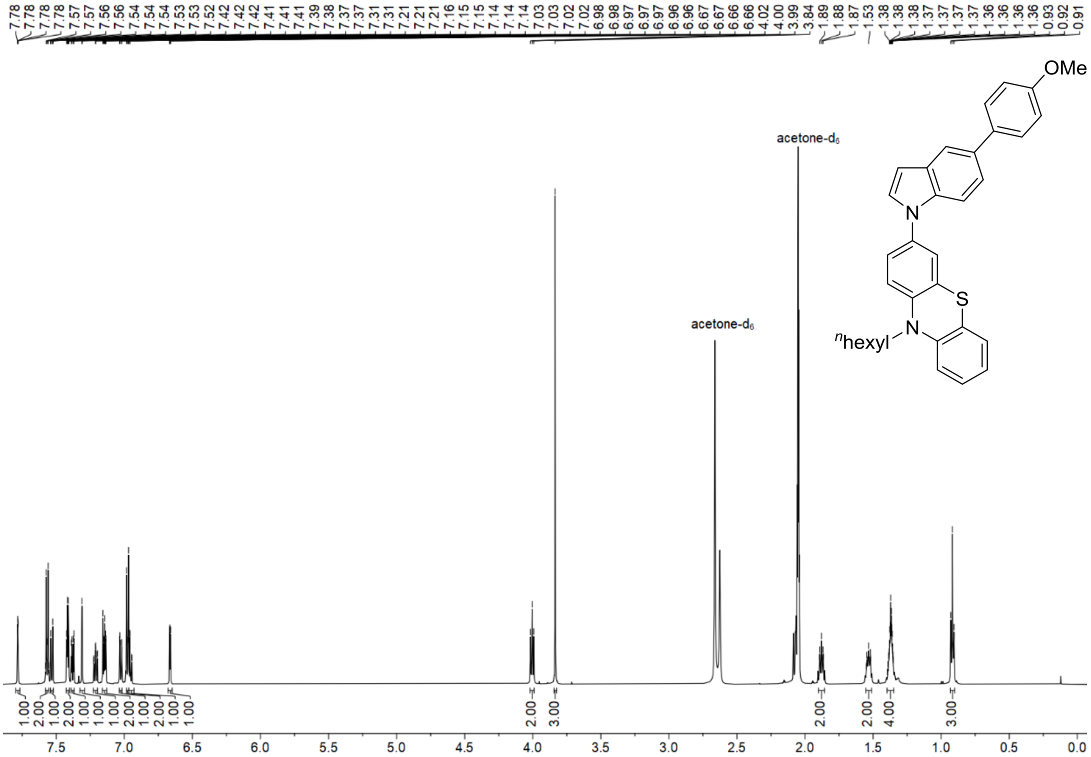

^{13}C NMR spectrum of 10-Hexyl-3-(5-(4-methoxyphenyl)-1*H*-indol-1-yl)-10*H*-phenothiazine (8l) (acetone-d_6, 150 MHz, 293 K)

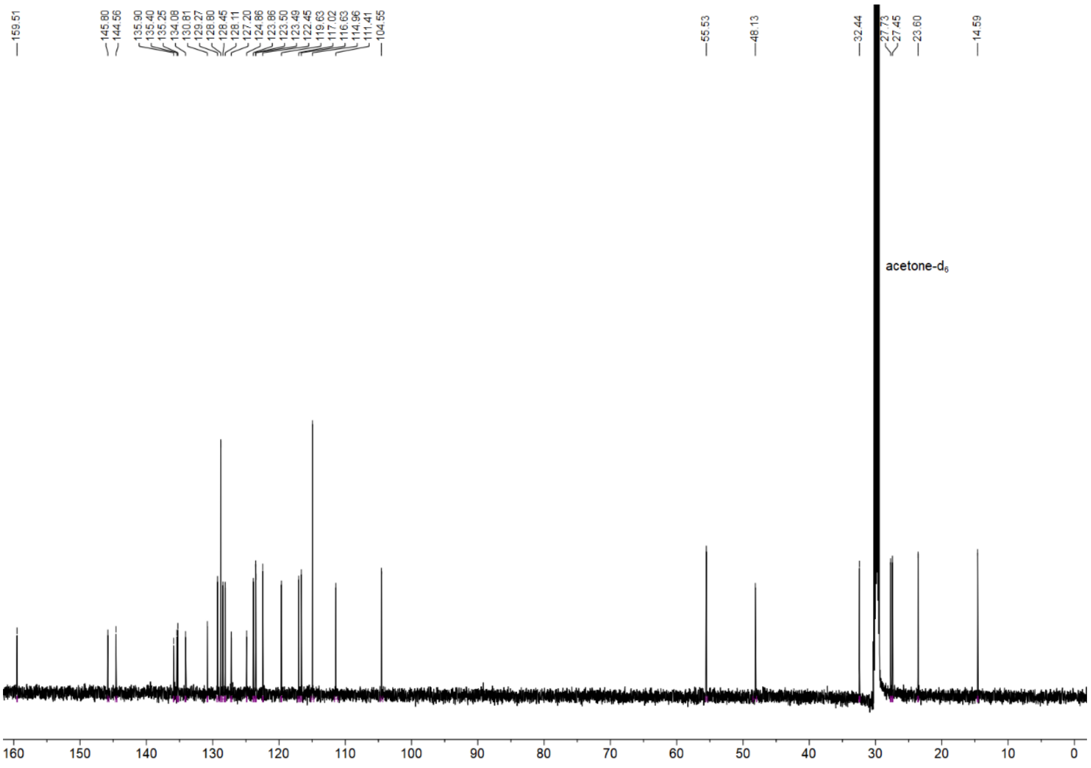

^{1}H NMR spectrum of 1-(4-methoxyphenyl)-5-(naphthalen-1-yl)-1*H*-indole (8m) (acetone-d_6, 300 MHz, 293 K)

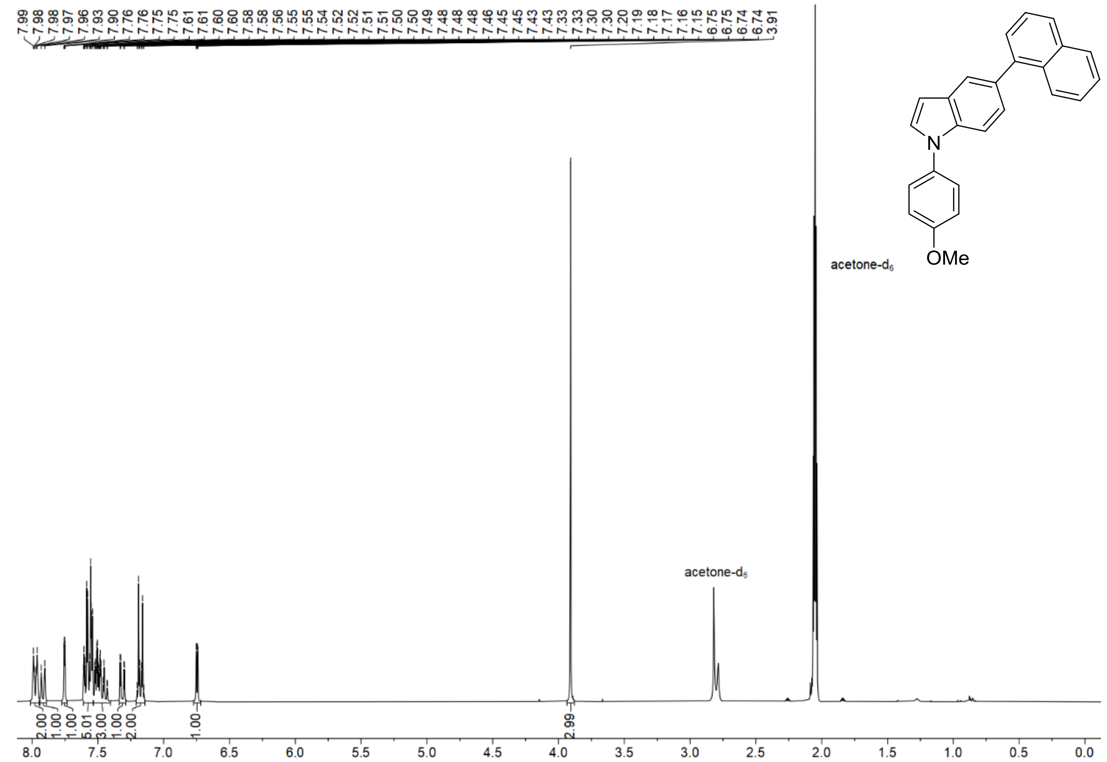

^{13}C NMR spectrum of 1-(4-methoxyphenyl)-5-(naphthalen-1-yl)-1*H*-indole (8m) (acetone-d_6, 75 MHz, 293 K)

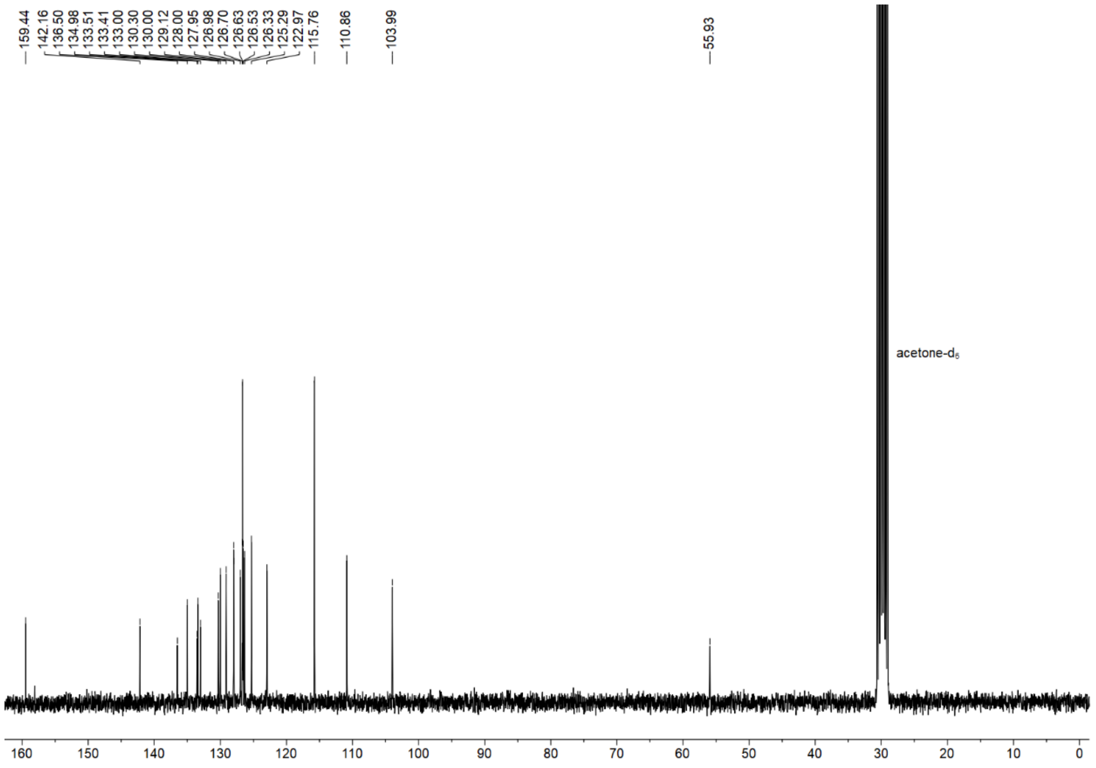

^{1}H NMR spectrum of 5-(4-methoxyphenyl)-1-(naphthalen-1-yl)-1*H*-indole (8n) (acetone-d$_6$, 600 MHz, 293 K)

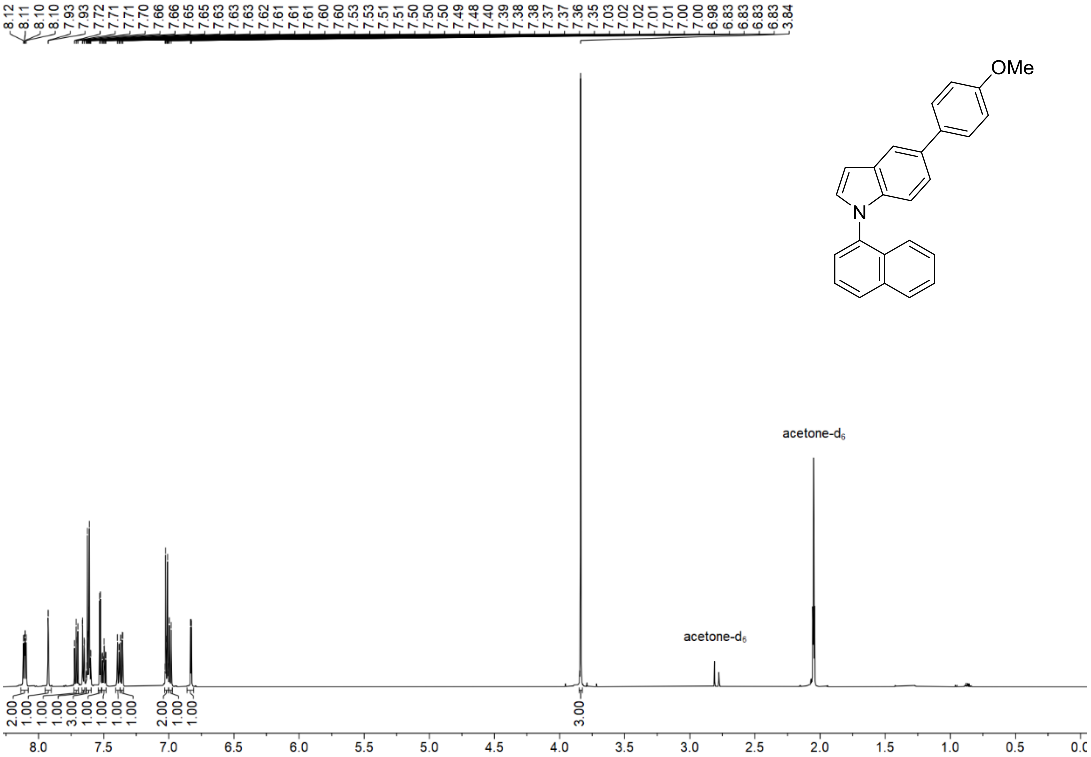

^{13}C NMR spectrum of 5-(4-methoxyphenyl)-1-(naphthalen-1-yl)-1*H*-indole (8n) (acetone-d$_6$, 150 MHz, 293 K)

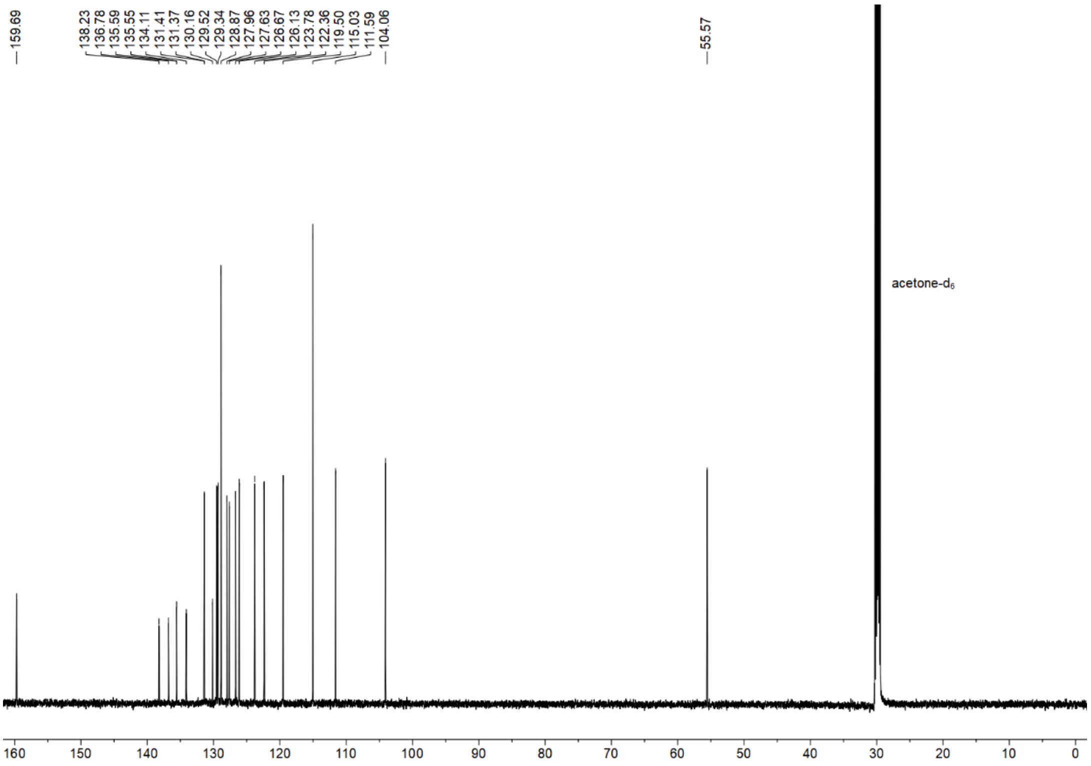

5. HPLC of 10-(4-Methoxyphenyl)-3-(*o*-tolyl)-10*H*-phenothiazine (4n)

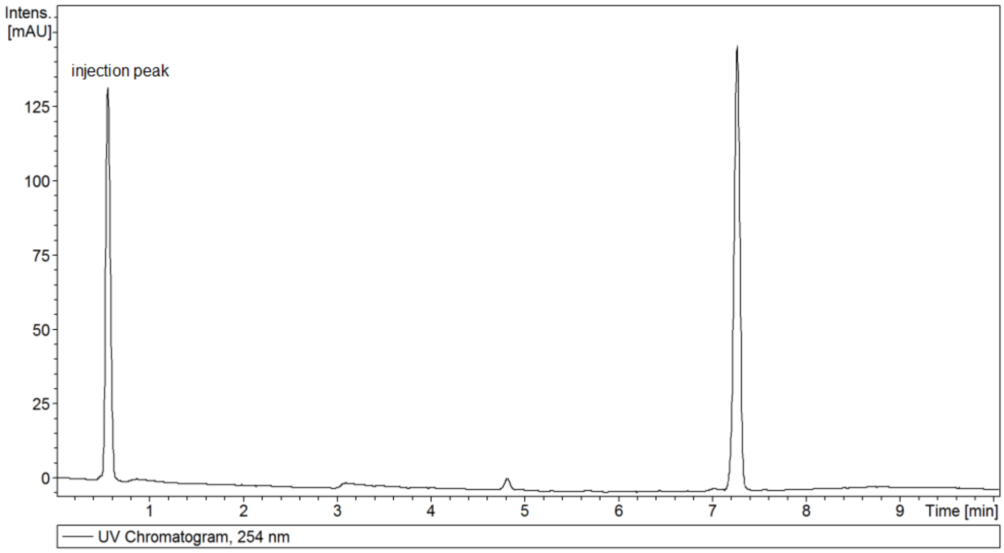

Literature

[1] J. Cymerman-Craig, W. P. Rogers, G. P. Warwick, *Aust. J. Chem.* **1955**, *8*, 252-257.

[2] X. Chen, J. Mihalic, P. Fan, L. Liang, M. Lindstrom, S. Wong, Q. Ye, Y. Fu, J. Jaen, J.-L. Chen, K. Dai, L. Li, *Bioorg. Med. Chem. Lett.* **2012**, *22*, 363-366.

[3] G. Trippé-Allard, J.-C. Lacroix, *Tetrahedron* **2013**, *69*, 861-866.

[4] S. Seifert, D. Schmidt, K. Shoyama, F. Würthner, *Angew. Chem.* **2017**, *129*, 7703-7708; *Angew. Chem. Int. Ed.* **2017**, *56*, 7595-7600;

[5] M. Sailer, A. W. Franz, T. J. J. Müller, *Chem. Eur. J.* **2008**, *14*, 2602-2614.

[6] a) W.-J. Yoo, T. Tsukamoto, S. Kobayashi, *Org. Lett.* **2015**, *17*, 3640-3642; b) S. Maddala, C.-L. Chung, S.-Y. Wang, K. Kollimalayan, H.-L. Hsu, P. Venkatakrishnan, C.-P. Chen, Y. J. Chang, *Chem. Mater.* **2020**, *32*, 127-138.

[7] P. Xu, E.-U. Würthwein, C. G. Daniliuc, A. Studer, *Angew. Chem.* **2017**, *129*, 14060-14063; *Angew. Chem. Int. Ed.* **2017**, *56*, 13872-13875;
